# Divarasib plus cetuximab in *KRAS G12C*-positive colorectal cancer: a phase 1b trial

**DOI:** 10.1038/s41591-023-02696-8

**Published:** 2023-12-05

**Authors:** Jayesh Desai, Guzman Alonso, Se Hyun Kim, Andres Cervantes, Thomas Karasic, Laura Medina, Einat Shacham-Shmueli, Rasha Cosman, Alejandro Falcon, Eelke Gort, Tormod Guren, Erminia Massarelli, Wilson H. Miller, Luis Paz-Ares, Hans Prenen, Alessio Amatu, Chiara Cremolini, Tae Won Kim, Victor Moreno, Sai-Hong I. Ou, Alessandro Passardi, Adrian Sacher, Armando Santoro, Rafal Stec, Susanna Ulahannan, Kathryn Arbour, Patricia Lorusso, Jia Luo, Manish R. Patel, Yoonha Choi, Zhen Shi, Sandhya Mandlekar, Mark T. Lin, Stephanie Royer-Joo, Julie Chang, Tomi Jun, Neekesh V. Dharia, Jennifer L. Schutzman, GO42144 Investigator and Study Group, Sae-Won Han

**Affiliations:** 1 Peter MacCallum Cancer Centre and Sir Peter MacCallum Department of Oncology, The University of Melbourne, Melbourne, Victoria Australia; 2 Vall d’Hebron Institute of Oncology (VHIO), Vall d’Hebron University Hospital, Barcelona, Spain; 3 Seoul National University Bundang Hospital, Seongnam, South Korea; 4 Hospital Clinico Universitario De Valencia, Valencia, Spain; 5 Abramson Cancer Center, University Of Pennsylvania, Philadelphia, PA USA; 6 Medical Oncology Intercenter Unit, Regional and Virgen de la Victoria University Hospitals, IBIMA, Málaga, Spain; 7 Sheba Medical Center, Sackler School of Medicineó, Tel Aviv University, Tel Aviv, Israel; 8 The Kinghorn Cancer Centre, St. Vincent’s Hospital and School of Medicine, University of New South Wales, Sydney, Australia; 9 Hospital Universitario Virgen del Rocio, Sevilla, Spain; 10 Universitair Medisch Centrum Utrecht, Utrecht, Netherlands; 11 Oslo University Hospital Radiumhospitalet, Oslo, Norway; 12 City of Hope - Comprehensive Cancer Center, Duarte, CA USA; 13 Lady Davis Institute and Segal Cancer Center, Jewish General Hospital, McGill University, Montreal, Quebec Canada; 14 Hospital Universitario 12 de Octubre, H120-CNIO Lung Cancer Unit, Universidad Complutense and Ciberonc, Madrid, Spain; 15 University Hospital Antwerp, Edegem, Belgium; 16 Haematology and Oncology Division, Grande Ospedale Metropolitano Niguarda, Milan, Italy; 17 University of Pisa, Pisa, Italy; 18 Department of Oncology, Asan Medical Center, University of Ulsan, Seoul, South Korea; 19 START MADRID-FJD, Hospital Universitario Fundacion Jimenez Diaz, Madrid, Spain; 20 University of California Irvine School of Medicine, Chao Family Comprehensive Cancer Center, Orange, CA USA; 21 Department of Medical Oncology, IRCCS Istituto Romagnolo per lo Studio dei Tumori (IRST) ‘Dino Amadori’, Meldola, Italy; 22 Princess Margaret Cancer Centre, University Health Network, Toronto, Canada, Department of Medicine & Department of Immunology, University of Toronto, Toronto, Ontario Canada; 23 Humanitas University and IRCCS Humanitas Research Hospital-Humanitas Cancer Center, Milan, Italy; 24 Biokinetica, Przychodnia Jozefow, Józefów, Poland; 25 Warsaw Medical University, Warsaw, Poland; 26 Stephenson Cancer Center, Oklahoma City, OK USA; 27 Sarah Cannon Research Institute, Nashville, TN USA; 28 Memorial Sloan Kettering Cancer Center, New York City, NY USA; 29 Yale Cancer Center, Yale University, New Haven, CT USA; 30 Dana-Farber Cancer Institute, Harvard Medical School, Boston, MA USA; 31 Florida Cancer Specialists/Sarah Cannon Research Institute, Sarasota, FL USA; 32 Genentech, South San Francisco, CA USA; 33 Seoul National University Hospital and Seoul National University Cancer Research Institute, Seoul, South Korea

**Keywords:** Drug development, Colorectal cancer

## Abstract

*KRAS G12C* mutation is prevalent in ~4% of colorectal cancer (CRC) and is associated with poor prognosis. Divarasib, a KRAS G12C inhibitor, has shown modest activity as a single agent in *KRAS G12C*-positive CRC at 400 mg. Epidermal growth factor receptor has been recognized as a major upstream activator of RAS–MAPK signaling, a proposed key mechanism of resistance to KRAS G12C inhibition in CRC. Here, we report on divarasib plus cetuximab (epidermal growth factor receptor inhibitor) in patients with *KRAS G12C*-positive CRC (*n* = 29) from arm C of an ongoing phase 1b trial. The primary objective was to evaluate safety. Secondary objectives included preliminary antitumor activity. The safety profile of this combination was consistent with those of single-agent divarasib and cetuximab. Treatment-related adverse events led to divarasib dose reductions in four patients (13.8%); there were no treatment withdrawals. The objective response rate was 62.5% (95% confidence interval: 40.6%, 81.2%) in KRAS G12C inhibitor-naive patients (*n* = 24). The median duration of response was 6.9 months. The median progression-free survival was 8.1 months (95% confidence interval: 5.5, 12.3). As an exploratory objective, we observed a decline in *KRAS G12C* variant allele frequency associated with response and identified acquired genomic alterations at disease progression that may be associated with resistance. The manageable safety profile and encouraging antitumor activity of divarasib plus cetuximab support the further investigation of this combination in *KRAS G12C*-positive CRC.

ClinicalTrials.gov identifier: NCT04449874

## Abstract (web summary)

In an arm of a phase 1b trial, the combination of divarasib, a KRAS G12C inhibitor, and cetuximab, an epidermal growth factor receptor inhibitor, was well tolerated with an encouraging overall response rate of 62.5% in patients with *KRAS G12C*-positive colorectal cancer.

## Main

The Kirsten rat sarcoma virus oncogene homologue (KRAS) protein is a guanosine triphosphatase (GTPase) that cycles between the active GTP-bound and inactive GDP-bound states to regulate cell proliferation, migration and survival^1,2^. The glycine-to-cysteine mutation at position 12 (p.Gly12Cys) of the KRAS protein favors the active GTP-bound state, increasing downstream oncogenic signaling and uncontrolled cell growth. Oncogenic *KRAS G12C* mutations occur in ~4% of patients with CRC, and are associated with poor prognosis^3–7^. Patients with CRC who have tumors harboring a *KRAS* mutation (including *KRAS G12C*) do not benefit and thus are not eligible for anti-epidermal growth factor receptor-based therapies^8,9^.

Divarasib (GDC-6036) is an orally bioavailable, covalent KRAS G12C inhibitor that turns off its oncogenic signaling by irreversibly locking the protein in an inactive state. In vitro studies have also shown that divarasib is 5 to 20 times as potent and up to 50 times as selective as compared to the KRAS G12C inhibitors sotorasib and adagrasib^10^. Single-agent divarasib treatment at 400 mg achieved a confirmed objective response rate (ORR) of 56.4% in patients with non-small cell lung cancer and 35.9% in patients with CRC, with a median progression-free survival (PFS) of 13.1 and 6.9 months, respectively^11^.

Despite the encouraging antitumor activity with single-agent divarasib in patients with CRC, adaptive feedback reactivation of RAS–MAPK signaling, a proposed key mechanism of resistance to KRAS G12C inhibition in CRC, occurs frequently and ultimately limits efficacy^12–14^. Preclinical studies have identified EGFR as a major mediator of adaptive feedback, and concomitant blockade of EGFR has been shown to enhance the antitumor activity of KRAS G12C inhibition^12,15^. Therefore, the combination of an EGFR inhibitor with a KRAS G12C inhibitor may more effectively inhibit the KRAS–MAPK pathway, prevent adaptive feedback and lead to more robust clinical responses. Clinical studies of other KRAS G12C inhibitors, such as sotorasib and adagrasib, support this hypothesis, with higher response rates observed when used in combination with an EGFR inhibitor compared to when used as single-agent treatment in patients with CRC^16–20^.

Divarasib in combination with other anticancer therapies is currently being evaluated in an ongoing phase 1b study in patients with advanced or metastatic solid tumors harboring a *KRAS G12C* mutation. Here, we report results from arm C patients with CRC who received divarasib in combination with cetuximab, an EGFR inhibitor. The primary objective of this study was to evaluate safety; secondary objectives included characterization of preliminary antitumor activity and the pharmacokinetic profile, and exploratory objectives included the characterization of biomarkers of response and resistance.

## Results

### Patient disposition and baseline demographics

**Fig. 1:**
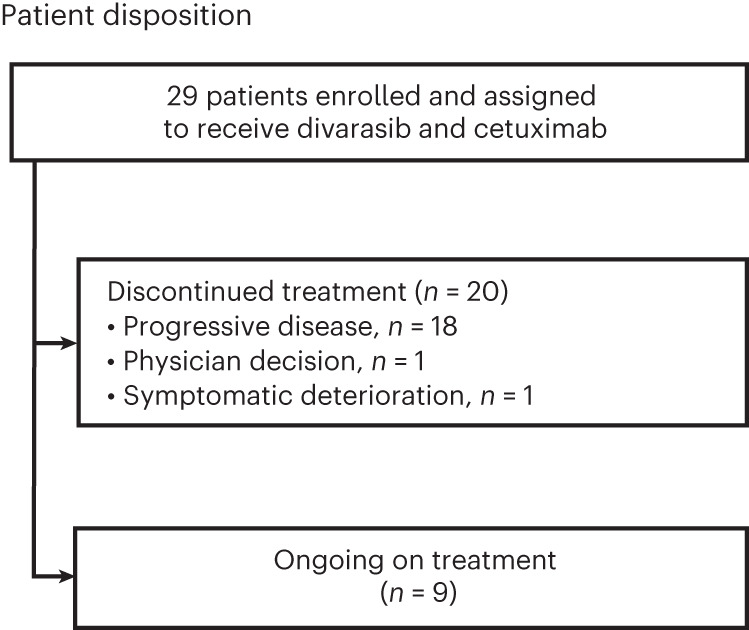
Patient disposition. Patient disposition for all 29 patients that were enrolled and received divarasib plus cetuximab.

A total of 29 patients were enrolled into arm C from 17 sites in 10 countries between 28 July 2021 and 07 October 2022 (Fig. 1). Key inclusion criteria were CRC with documentation of the *KRAS G12C* mutation from either central testing of blood samples (FoundationOne Liquid CDx/F1LCDx) or local testing of tumor tissue or blood samples (using a validated molecular testing method) and evaluable or measurable disease per Response Evaluation Criteria in Solid Tumors (RECIST) v1.1. Patients with prior KRAS G12C inhibitor treatment must not have discontinued due to toxicity related to the prior KRAS G12C inhibitor. Key exclusion criteria were active, untreated central nervous system (CNS) metastases (previously treated CNS metastases were allowed); treatment with another anticancer therapy within 3 weeks or five half-lives before initiation of study treatment, whichever is shorter; and radiation therapy as cancer therapy within 4 weeks before initiation of divarasib.

Patients received oral divarasib at 200 mg (*n* = 3) or 400 mg (*n* = 26) once daily, plus intravenous cetuximab (400 mg/m^2^ on cycle 1 day 1 (C1D1) only, then 250 mg/m^2^ once weekly) in 21-d cycles. The selected expansion dose for the divarasib combination with cetuximab was 400 mg and ongoing dose investigations are still occurring in other combinations. The data cutoff date was 01 April 2023 with an enrollment cutoff of 07 October 2022. This analysis included patients who received at least one dose of divarasib and cetuximab. The median time on divarasib treatment was 7.1 months (range: 2.6–12.6), and on cetuximab treatment was 6.7 months (range: 0.5–12.2). Study treatment was discontinued in 20 patients (69.0%), with the reasons being progressive disease according to RECIST (*n* = 18, 62.1%), clinical progression (*n* = 1, 3.4%) or physician decision (*n* = 1, 3.4%).

Baseline demographics are summarized in Table 1. The median age was 59 years (range: 33–87) and patients received a median of 2 (range: 1–8) prior systemic therapies. All 29 (100.0%) patients received prior 5-fluorouracil (5-FU) or capecitabine, 27 (93.1%) received oxaliplatin, 24 (82.8%) received irinotecan, 24 (82.8%) received bevacizumab and 5 (17.2%) received a prior KRAS G12C inhibitor as a single agent or as a combination therapy (2 patients had single-agent divarasib, 1 had single-agent sotorasib, 1 had sotorasib with bevacizumab, and 1 had adagrasib with an inhibitor of Src homology region 2–containing protein tyrosine phosphatase-2 (SHP2)).

**Table 1:** Baseline demographics and disease characteristics

	All patients (*N* = 29)
Median age (range), years	59 (33–87)
Female, *n* (%)	15 (51.7)
Race, *n* (%)	
White	20 (69.0)
Asian	6 (20.7)
Multiple	1 (3.4)
Unknown	2 (6.9)
ECOG, *n* (%)	
0	16 (55.2)
1	13 (44.8)
Location of tumor, *n* (%)	
Left side	18 (62.1)
Right side^a^	9 (31.0)
Unknown	2 (6.9)
Prior systemic therapies, *n* (%)	
1	5 (17.2)
2	10 (34.5)
3	4 (13.8)
≥4	10 (34.5)
Prior 5-FU/capecitabine, *n* (%)	29 (100.0)
Prior oxaliplatin, *n* (%)	27 (93.1)
Prior irinotecan, *n* (%)	24 (82.8)
Prior bevacizumab, *n* (%)	24 (82.8)
Prior KRAS G12C inhibitor, *n* (%)	5 (17.2)

^a^‘Right side’ includes tumor location reported as both right side and midline. ECOG, Eastern Cooperative Oncology Group.

### Safety

The primary objective of this study was to evaluate the safety of divarasib plus cetuximab. No dose-limiting toxicities were reported. All (100.0%) patients experienced at least one treatment-related adverse event (TRAE), with the majority of events being grade 1–2 (91.6%). The most common TRAEs included rash (96.6%; grouped term including dermatitis acneiform, rash, rash pustular, rash follicular and rash maculo-papular), diarrhea (82.8%), nausea (72.4%) and vomiting (48.3%). TRAEs occurring in ≥10% of patients are summarized in Table 2 and fully described in Extended Data Table 1. Grade 3 TRAEs occurred in 11 patients (37.9%) and included diarrhea (*n* = 3), increased lipase (*n* = 2), rash (*n* = 2) and abdominal pain, upper abdominal pain, dry skin, headache, hypomagnesemia, infusion-related reaction, insomnia, paronychia, pruritus and white blood cell count decrease (*n* = 1 for each). Grade 4 TRAEs occurred in 2 patients (6.9%) and included hypomagnesemia (*n* = 1) and neutropenia (*n* = 1). No grade 5 TRAEs occurred. Serious adverse events (AEs) occurred in 7 patients (24.1%), with none related to study treatment. Three deaths due to CRC progression occurred during safety follow-up.

**Table 2:** TRAEs occurring in ≥10% of patients

	TRAEs	Grade 3–4^b^ TRAEs
Total number of patients with at least one AE, *n* (%)	29 (100)	13 (44.8)
Rash^a^	28 (96.6)	2 (6.9)
Diarrhea	24 (82.8)	3 (10.3)
Nausea	21 (72.4)	0
Vomiting	14 (48.3)	0
Dry skin	10 (34.5)	1 (3.4)
Paronychia	6 (20.7)	1 (3.4)
Hypomagnesemia	4 (13.8)	2 (6.9)
Pruritus	4 (13.8)	1 (3.4)
Infusion-related reaction	4 (13.8)	1 (3.4)
Asthenia	4 (13.8)	0
Fatigue	4 (13.8)	0
Abdominal pain	3 (10.3)	1 (3.4)
Amylase increased	3 (10.3)	0
Pyrexia	3 (10.3)	0
Dysgeusia	3 (10.3)	0

^a^Rash grouped terms: dermatitis acneiform, rash, rash pustular, rash follicular and rash maculo-papular.

^b^No grade 5 TRAEs reported.

TRAEs led to divarasib dose modifications (interruption, reduction or withdrawal) in 10 patients (34.5%), with divarasib dose interruptions in 7 patients (24.1%), divarasib dose reductions in 4 patients (13.8%) and no divarasib treatment withdrawals. TRAEs that led to divarasib dose reduction included diarrhea in 3 patients (10.3%, all grade 3) and vomiting in 1 patient (3.4%, grade 1). TRAEs led to cetuximab dose modifications (interruption, reduction or withdrawal) in 11 patients (37.9%), with cetuximab dose interruptions in 9 patients (31.0%), cetuximab dose reductions in 4 patients (13.8%) and cetuximab withdrawal in 1 patient (3.4%). TRAEs that led to cetuximab dose reduction include infusion-related reactions in 2 patients (6.9%, one grade 2 and one grade 1), and dermatitis acneiform, paronychia and pustular rash in 1 patient each (3.4%, grade 2 pustular rash, grade 2 paronychia and grade 3 dermatitis acneiform). Treatment-related grade 3 rash led to cetuximab withdrawal in 1 patient who continued to receive divarasib.

### Antitumor activity

**Fig. 2:**
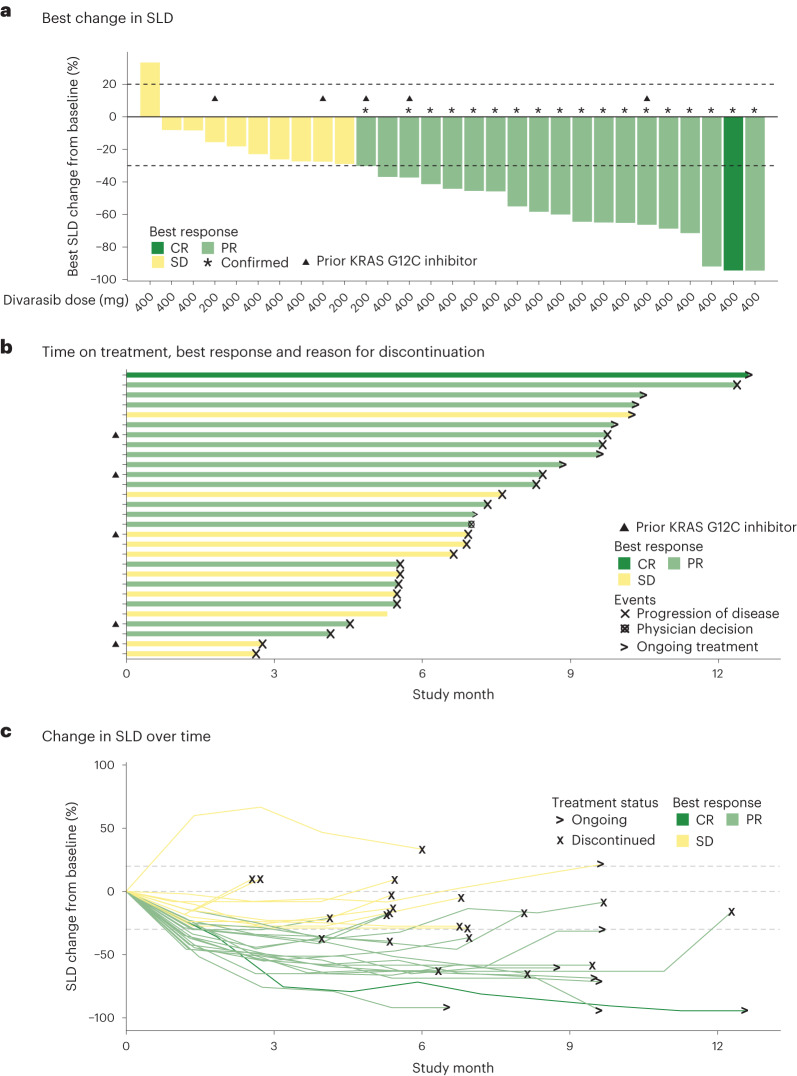
Antitumor activity for all patients. **a**, Waterfall plot showing the best percentage decrease from baseline in the tumor burden (defined as the sum of the longest diameters of all target lesions) in all 29 patients. **b**, Swimmer plot showing the time on study treatment, best response, and reason for treatment discontinuation for all 29 patients. **c**, Spider plot of the percentage changes from baseline in sum of tumor diameters over time in all 29 patients.

**Fig. 3:**
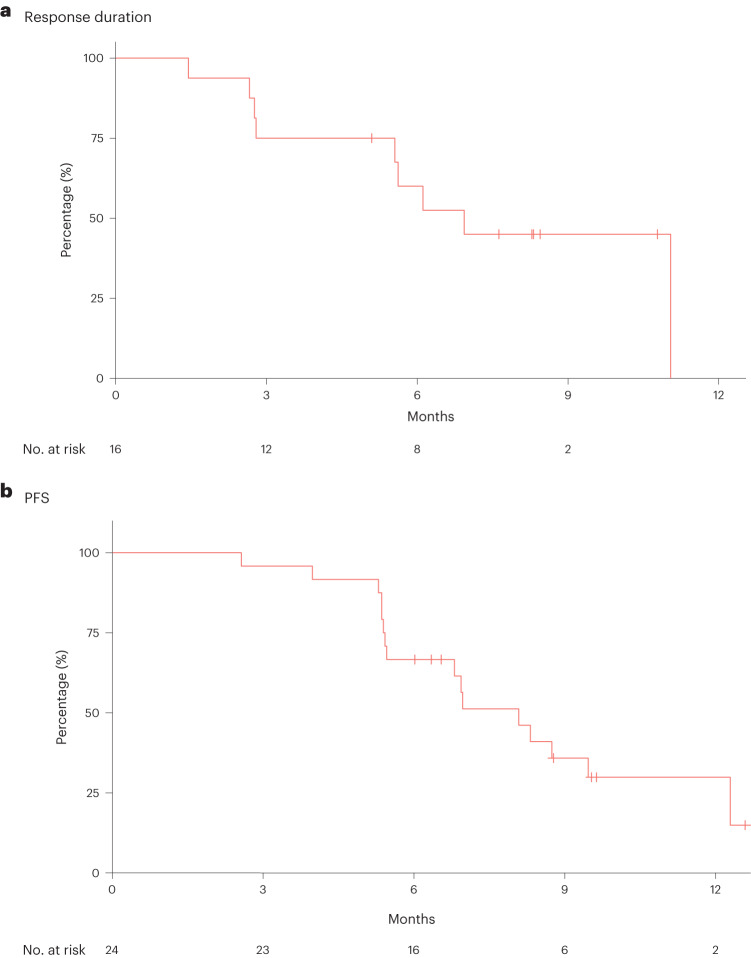
Duration of response and PFS for KRAS G12C inhibitor-naive patients. **a**, Kaplan–Meier plot for duration of response for the 16 KRAS G12C inhibitor-naive patients who experienced a CR or PR. **b**, Kaplan–Meier plot for median PFS for all 24 KRAS G12C inhibitor-naive patients.

A secondary objective of this study was to assess the preliminary antitumor activity of divarasib plus cetuximab. Antitumor activity was determined by the investigator according to RECIST v1.1 (see the study end points in the Methods for further definitions), and all 29 patients had measurable disease at baseline (Fig. 2). Of the 24 patients who had not previously received a KRAS G12C inhibitor before enrollment, 1 patient received divarasib at 200 mg in combination with cetuximab and 23 patients received divarasib at 400 mg in combination with cetuximab. Among the 24 patients, 1 patient (4.2%; 1/1 confirmed) had a complete response (CR), 15 (62.5%; 14/15 confirmed) had a partial response (PR), and 8 (33.3%) had stable disease (SD) as their best response, for a confirmed ORR of 62.5% (95% confidence interval (CI): 40.6%, 81.2%). As an ad hoc end point, the median time to response was 1.4 months (range: 1.2–9.8). The median duration of response was 6.9 months (95% CI: 5.6, not estimable; Fig. 3). The median PFS was 8.1 months (95% CI: 5.5, 12.3). One patient experienced a 33.3% increase from baseline in the target lesion sum of diameters, but was assessed with a best response of SD based on the investigator’s interpretation of RECIST v1.1 in light of the response observed in the non-target lesions. Among the 5 patients who had received KRAS G12C inhibitors before enrollment, 3 (60.0%; 3/3 confirmed) had a PR and 2 (40.0%) had SD as their best response.

### Pharmacokinetics

**Extended Data Fig. 1:**
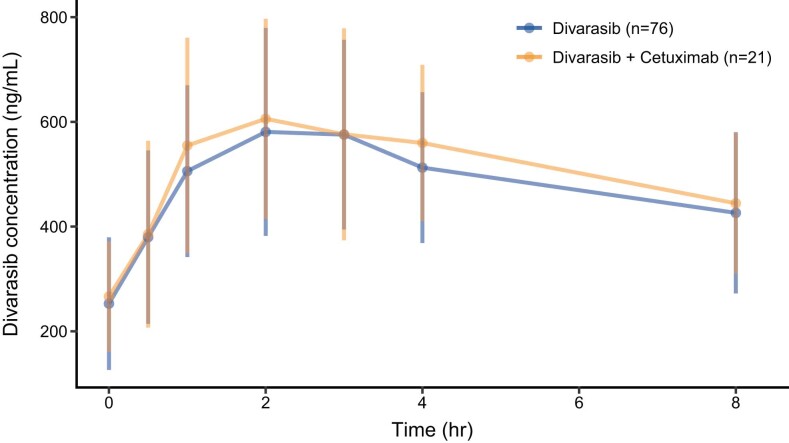
Mean (SD) Plasma Concentration-Time Profiles of Divarasib as a Single Agent and in Combination with Cetuximab Following Multiple Doses. Multiple-dose pharmacokinetic profile of divarasib was obtained on either C1D8 or C2D1 from blood samples collected over an 8 hour period following administration of divarasib 400 mg QD (n = 76 patients)^1^ or divarasib 400 mg QD plus 250 mg/m^2^ of cetuximab QW (n = 21 patients). Time (hr) is shown as nominal time and the error bars represent standard deviation around the mean divarasib plasma concentration (ng/mL) measured at each time point during the 8 hour post-dose interval.

Another secondary objective in this study was to characterize the pharmacokinetic profile of divarasib plus cetuximab. We previously reported that the mean half-life of a single dose of divarasib 400 mg administered as a single agent was 17.6 ± 2.7 h and the corresponding accumulation index after daily dosing to steady state (area under the concentration–time curve from 0 to 24 h (R_AUC0–24_)) was 1.4 ± 0.4 (ref. ^11^). The steady-state pharmacokinetic profile and R_AUC0–24_ of divarasib (400 mg once daily) in combination with cetuximab were similar to single-agent divarasib (Extended Data Table 2 and Extended Data Fig. 1).

### Biomarker analysis

Circulating tumor DNA (ctDNA) is an emerging biomarker for monitoring treatment effect, tracking tumor evolution and identifying potential mechanisms of resistance to treatment. As an exploratory objective in this study, retrospective ctDNA profiling was conducted with longitudinal plasma samples collected at baseline (C1D1), on-treatment (multiple time points) and end-of-treatment (EoT) visits.

**Fig. 4:**
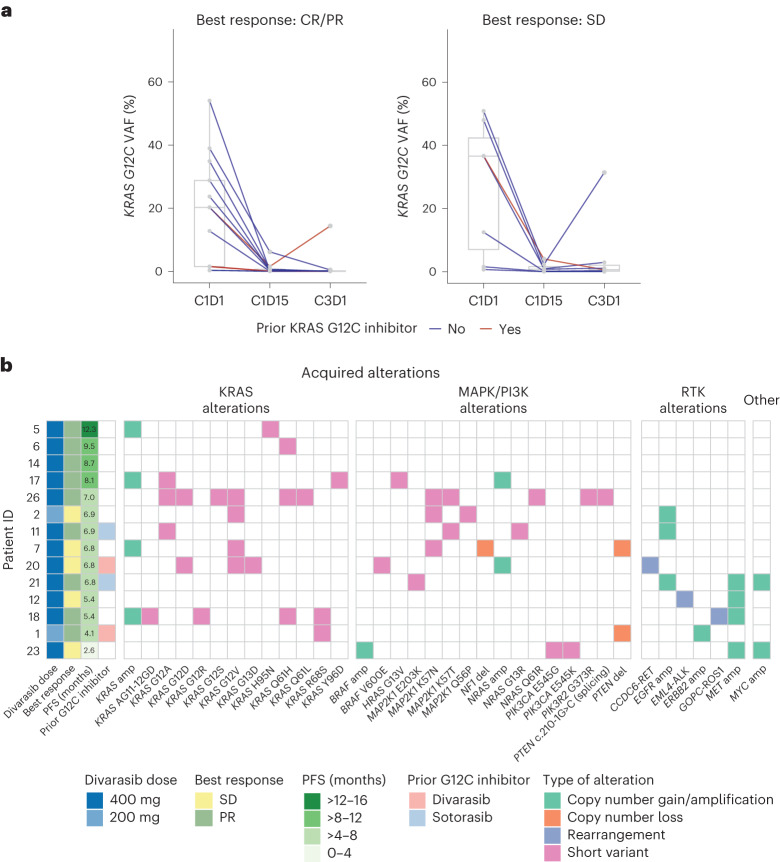
Biomarker analysis of ctDNA. **a**, Shown is the *KRAS G12C* VAF at baseline (C1D1) and early treatment time points (C1D15, C3D1) among patients with a detectable *KRAS G12C* mutation from cfDNA at C1D1 (*n* = 20 shown, each line represents one patient). Two patients with missing C1D15 plasma samples are not shown in the plot. Box-and-whisker plots at each time point in each indication show the median (center line) with the minima and maxima box boundaries representing the 25th and 75th percentiles; whiskers represent the minimum and maximum values of the data that are within 1.5 times the interquartile range under the 25th and over the 75th percentiles. **b**, Shown are putative genetic mechanisms of acquired resistance to divarasib plus cetuximab combination treatment among 14 patients who had an EoT visit before 01 May 2023. Each row represents one patient with the first four columns describing assigned divarasib dose, best response, PFS, prior KRAS G12C inhibitor use and subsequent columns indicating acquired genomic alterations at EoT.

*KRAS G12C* variant allele frequency (VAF) from circulating cell-free DNA (cfDNA) was monitored at baseline (C1D1) and at early on-treatment time points (cycle 1 day 15 (C1D15) and cycle 3 day 1 (C3D1)). At C1D1, the *KRAS G12C* mutation was detected from cfDNA in 22 of 25 patients evaluated. Decline in *KRAS G12C* VAF as early as C1D15 was observed in patients who had a response or SD. At C3D1, the VAF of *KRAS G12C* was <0.5% in 17 of these 22 patients (77.3%) including patients with a response or SD (Fig. 4a).

To explore the mechanisms of potential acquired resistance, we conducted ctDNA profiling from paired baseline and disease progression/EoT plasma samples. Of the 14 patients profiled, 13 patients (92.9%) had at least one acquired genomic alteration that may be associated with treatment resistance at EoT. Ten patients had at least one alteration in the *KRAS* gene (excluding the *G12C* mutation), including copy number gain/amplification, non-*G12C* pathogenic mutations that can result in oncogenic activation of KRAS, and secondary mutations that may diminish the binding of divarasib to the KRAS G12C protein. Other acquired genomic alterations observed at EoT include alterations in *NRAS*/*HRAS* (observed in 4/14 patients), genes in the MAPK pathway (8/14 patients), PI3K pathway (4/14 patients), RTK pathway (8/14 patients) and others (for example, *MYC* copy number gain; 2/14 patients; Fig. 4b).

## Discussion

The *KRAS G12C* mutation is associated with worse overall survival in patients with CRC and is readily identified through standard-of-care testing, thus highlighting an area of need for more effective and targeted therapeutic approaches^4^. Current first-line standard of care for *KRAS G12C*-positive CRC includes 5-FU-based chemotherapy with irinotecan, oxaliplatin and/or capecitabine, but is limited by low tumor-specific selectivity and systemic toxicity^21^. Several KRAS G12C inhibitors, including divarasib, sotorasib and adagrasib, are currently being investigated as a single agent and in combination with EGFR inhibitors to target this unmet need, although they vary in their safety, antitumor activity and clinical benefit profiles.

The combination of divarasib and cetuximab appeared to be well tolerated, and AEs were manageable with supportive medications and dose modifications. The most common AEs observed in this study were low-grade gastrointestinal AEs and rash, which were consistent with single-agent divarasib and cetuximab safety profiles, respectively^11,22^. No patients withdrew divarasib treatment due to TRAEs and only 1 patient (3.4%) withdrew from cetuximab treatment due to a TRAE. Furthermore, there was a low rate of divarasib dose reductions (13.8%), with interruptions in 24.1% of patients. Among patients treated with adagrasib plus cetuximab in KRYSTAL-1 (*n* = 32), no patients discontinued adagrasib and 5 patients (16%) discontinued cetuximab due to TRAEs, with adagrasib dose reductions reported in 10 patients (31%)^20^. In the CodeBreaK300 study, TRAEs led to treatment discontinuation in 3.8% of patients in the 960 mg sotorasib plus panitumumab arm and 1.9% in the 240-mg sotorasib plus panitumumab arm^17^.

The improved antitumor activity observed with divarasib plus cetuximab in this study compared with single-agent divarasib is consistent with the hypothesis of EGFR-mediated adaptive feedback reactivation of the RAS–MAPK pathway following KRAS G12C inhibition. In patients with CRC who had not received prior KRAS G12C inhibitor treatment, single-agent divarasib achieved a response rate of 35.9%^11^, while the combination of divarasib and cetuximab achieved a response rate of 62.5%. Although cross-trial comparisons are difficult due to differences in patient populations, the ORR reported in this trial is numerically higher compared to that reported with 960 mg sotorasib plus panitumumab (26.4%) and adagrasib plus cetuximab (46%), as well as single-agent treatment of patients with CRC (35.9% for divarasib, 9.7% for sotorasib and 19% for adagrasib)^16,17,20^. Similarly, the median PFS of 8.1 months with divarasib observed in this trial is the longest reported for a KRAS G12C inhibitor combined with an EGFR inhibitor in patients with CRC (5.6 months (95% CI: 4.2, 6.3) with sotorasib plus panitumumab and 6.9 months (95% CI: 5.4, 8.1) for adagrasib plus cetuximab)^17,20^. Although preliminary evidence with the divarasib plus cetuximab combination is encouraging, this study was limited by the small number of patients enrolled into this arm and the lack of comparison of this divarasib and cetuximab combination to a standard-of-care treatment in *KRAS G12C*-positive CRC.

Divarasib has been shown to be 5 to 20 times more potent in vitro as compared to sotorasib and adagrasib^10^. This difference in potency may explain the numerically higher ORRs and longer PFS with divarasib compared to sotorasib and adagrasib, as a single-agent and in combination with an EGFR inhibitor in patients with CRC. This is further supported by a numerically higher ORR seen with divarasib in patients with non-small cell lung cancer (56.4%) compared with sotorasib (37% in a phase 2 trial and 28% in a phase 3 trial) and adagrasib (43% in a phase 2 trial)^11,23–25^. This current trial also included 5 patients who previously received a KRAS G12C inhibitor, with a PR observed in 3 of the 5 patients (60.0%) when treated with divarasib plus cetuximab. In contrast, patients who previously received single-agent adagrasib therapy showed no additional responses when subsequently treated with adagrasib plus cetuximab combination therapy^20^.

Divarasib plus cetuximab treatment led to a decrease in *KRAS G12C* ctDNA levels. Of note, divarasib plus cetuximab combination therapy showed a larger decline in *KRAS G12C* ctDNA levels compared to single-agent divarasib (77.3% versus 45.7% of patients with detectable *KRAS G12C* ctDNA at C1D1 had *KRAS G12C* VAF decline to below 0.5% at C3D1)^11^. Upon disease progression with divarasib plus cetuximab, the majority (92.9%) of patients profiled had at least one genomic alteration that may be associated with treatment resistance similarly to two previous reports^26,27^, which is higher than that of patients treated with divarasib (30.2%) or other KRAS G12C inhibitors as single agent^11,28,29^. This observation may be due to a more potent treatment effect with the combination of divarasib and cetuximab imposing a stronger selective pressure and/or potentially increasing the adaptive mutability of the residual cancer cells^30^.

In conclusion, divarasib in combination with cetuximab demonstrated a manageable safety profile and promising clinical activity. The further improvements in antitumor activity demonstrated with the addition of cetuximab to divarasib may represent an effective strategy for overcoming resistance to KRAS G12C inhibitors. Divarasib is continuing to be investigated with other anticancer therapies for patients with CRC in this study, including bevacizumab, GDC-1971 (a SHP2 inhibitor) and inavolisib (a PI3Kα inhibitor). Additionally, a study is ongoing to explore the effects of the combination of divarasib plus cetuximab with or without chemotherapy (FOLFOX: 5-FU, leucovorin and oxaliplatin; or FOLFIRI: leucovorin, 5-FU and irinotecan; ClinicalTrials.gov: NCT04929223) in patients with *KRAS G12C*-positive CRC.

## Methods

### Study design

Divarasib plus cetuximab combination data are reported from arm C of an ongoing phase 1b, open-label, multicenter, dose-escalation and dose-expansion study of divarasib in combination with other anticancer therapies in patients with advanced or metastatic solid tumors that harbor a *KRAS G12C* mutation (NCT04449874).

Patients received oral divarasib 200 or 400 mg once daily and intravenous cetuximab (400 mg/m^2^ on C1D1, then 250 mg/m^2^ once weekly) in 21-d cycles until intolerable toxicity, disease progression or patient withdrawal. Patients were enrolled in a 3 + 3 dose-escalation design at 200 mg and 400 mg divarasib, then enrolled into dose-expansion cohorts of 400 mg divarasib. As described in the protocol, an Internal Monitoring Committee was involved to ensure that appropriate patient safety oversight is maintained throughout the conduct of this study. The redacted protocol is available in the Supplementary Information.

This study was conducted in full conformance with the ICH E6 guideline for Good Clinical Practice and the Declaration of Helsinki, or the applicable laws and regulations of the country in which the research is conducted, whichever affords the greater protection to the individual. The protocol was approved by the institutional review boards at City of Hope Comprehensive Cancer Center, Princess Margaret Cancer Center, Peter MacCallum Cancer Center, Jewish General Hospital, Universitair Medisch Centrum Utrecht, Biokinetica Przychodnia Jozefow, Istituto Scientifico Romagnolo per lo Studio y la Cura dei Tumori, Asst Grande Ospedale Metropolitano Niguarda, UZ Antwerpen, Hospital Universitario Virgen Del Rocio, Hospital Universitari Vall D’hebron, Seoul National University Hospital, Seoul National University Bundang Hospital, Hospital Clinico Universitario De Valencia, Hospital Universitario 12 De Octubre, Sheba Medical Center and Abramson Cancer Center. Patients provided signed informed consent before enrollment.

### Patients

Patients 18 years or older with locally advanced, recurrent or metastatic incurable adenocarcinoma of the colon or rectum harboring a *KRAS G12C* mutation documented by either central testing of blood samples (FoundationOne Liquid CDx/F1LCDx) or local testing of tumor tissue or blood samples (using a validated molecular testing method) were enrolled from 17 sites in 10 countries.

Patients were included based on the following inclusion criteria: disease that had progressed after at least one available standard therapy, or for which standard therapy has shown to be ineffective or intolerable, or for which a clinical trial of an investigational agent is a recognized standard of care; evaluable or measurable disease per RECIST v1.1; Eastern Cooperative Oncology Group performance status of 1 or lower (a five-point scale where higher numbers reflect greater disability); life expectancy ≥12 weeks; adequate hematologic and organ function within 14 d before initiation of study treatment defined as absolute neutrophil count ≥1,200/µl, hemoglobin ≥9 g dl^−1^, platelet count ≥100,000/µl, total bilirubin ≤1.5 times the upper limit of normal (ULN), serum albumin ≥2.5 g dl^−1^, aspartate transaminase (AST) and alanine transaminase (ALT) ≤ 2.5 times ULN (patients with documented liver metastases may have AST and ALT ≤ 5.0 times the ULN), serum creatinine ≤1.5 times the ULN or creatinine clearance ≥50 ml min^−1^ based on the Cockcroft–Gault estimation, and an international normalized ratio and activated partial thromboplastin time < 1.5 times the ULN (for those not receiving therapeutic anticoagulation); for women of childbearing potential to be abstinent or use contraception, and refrain from donating eggs/sperm for at least 6 months after divarasib and 2 months after cetuximab treatment; for nonsurgically sterile men to be abstinent or use contraception, and refrain from donating sperm for at least 4 months after divarasib and 2 months after cetuximab treatment; not have a known concomitant second oncogenic driver (for example, *BRAF*^V600E^ mutation, *ERBB2* amplification) as determined by Foundation Medicine; NGS assay or local testing of tumor tissue or blood samples (using a validated molecular testing method); and for patients required to provide pre-treatment and on-treatment biopsy samples, accessible lesion(s) that permit a total of at least two biopsy samples (pre-treatment and on-treatment) without unacceptable risk of a procedural complication. Patients with prior KRAS G12C inhibitor treatment must not have discontinued due to toxicity related to the prior KRAS G12C inhibitor and were required to provide tumor tissue specimens collected before and after treatment with the prior KRAS G12C inhibitor.

Patients were excluded based on the following criteria: inability/unwillingness to swallow pills; inability/unwillingness to comply with study or follow-up procedures; malabsorption syndrome or other condition that would interfere with enteral absorption; active, untreated CNS metastases (previously treated CNS metastases were allowed if they had measurable or evaluable disease outside the CNS, no history of intracranial or spinal cord hemorrhage, no ongoing requirement for corticosteroids for CNS metastases (with corticosteroids discontinued for ≥2 weeks before enrollment and no ongoing symptoms attributed to CNS metastases), no stereotactic radiation within 7 d or whole-brain radiation within 14 d before C1D1, and no evidence of interim progression between completion of CNS-directed therapy and screening radiographic study); leptomeningeal disease or carcinomatous meningitis; uncontrolled pleural effusion, pericardial effusion or ascites requiring recurrent drainage procedures biweekly or more frequently; any active infection that could impact patient safety or serious infection requiring intravenous antibiotics within 7 d before C1D1; clinical history of liver disease, including viral or other hepatitis, current alcohol abuse or cirrhosis; known HIV infection; any other diseases, active or uncontrolled pulmonary dysfunction, metabolic dysfunction, physical examination finding or clinical laboratory finding giving reasonable suspicion of a disease or condition that contraindicates the use of an investigational drug, that may affect the interpretation of the results, or renders the patients at high risk from treatment complications; uncontrolled hypercalcemia (>1.5 mmol^−1^ ionized calcium or 12 mg dl^−1^ calcium or ≥ corrected serum calcium ULN) or symptomatic hypercalcemia requiring continued use of bisphosphonate therapy or denosumab; traumatic injury or major surgical procedure within 4 weeks before C1D1; patients with chronic diarrhea, short bowel syndrome or upper gastrointestinal surgery including gastric resection, a history of inflammatory bowel disease or any active bowel inflammation (including diverticulitis); treatment with another anticancer therapy within 3 weeks or five half-lives before initiation of study treatment, whichever is shorter, or endocrine therapy within 2 weeks before initiation of study treatment, except for hormonal therapy with gonadotropin-releasing hormone agonists or antagonists for endocrine-sensitive cancers (approved kinase inhibitors may be used up to 2 weeks before initiation of study treatment, provided any drug-related toxicity has completely resolved); radiation therapy as cancer therapy within 4 weeks before initiation of study treatment; palliative radiation to bony metastases within 2 weeks before initiation of divarasib; AEs from prior anticancer therapy that have not resolved to grade 1 except for alopecia, vitiligo, endocrinopathy managed with replacement therapy or grade 2 peripheral neuropathy; history of other malignancy within 5 years before screening, with the exception of patients with a negligible risk of metastasis or death and/or treated with expected curative outcome; history of or active cardiovascular dysfunction, including history of stroke or transient ischemic attack within 6 months before first dose of study treatment, history of myocardial infarction within 6 months before first dose of study treatment, New York Heart Association Class III or IV cardiac disease or congestive heart failure requiring medication, uncontrolled arrhythmias, history of or active ventricular arrhythmia requiring medication, coronary heart disease that is symptomatic or unstable angina, congenital long QT syndrome or QT interval corrected through use of Fridericia’s formula >470 ms demonstrated by at least two electrocardiograms 30 min apart, or family history of sudden unexplained death or long QT syndrome, current treatment with medications that are well known to prolong the QT interval; pregnant or breastfeeding, or intending to become pregnant during the study or within 6 months after the final dose of divarasib (women of childbearing potential (including those who have had a tubal ligation) must have a negative serum pregnancy test result within 14 d before initiation of study drug); presence of appendiceal tumor; known hypersensitivity to study treatments; and history of idiopathic pulmonary fibrosis, organizing pneumonia (for example, bronchiolitis obliterans), drug-induced pneumonitis, or idiopathic pneumonitis, or evidence of active pneumonitis on screening chest computed tomography scan (history of radiation pneumonitis in the radiation field (fibrosis) was permitted).

### Study assessments

Safety was assessed through the evaluation of AEs (NCI CTCAE v5.0), changes in laboratory test results and changes in vital signs and electrocardiograms, and included all patients who received at least one dose of both study drugs. Attribution of AEs to study drug was determined by the investigator. For each AE requiring a dose modification, only one action taken with study drug was selected according to the following hierarchy: withdrawal, reduction and interruption. Preliminary antitumor activity was determined by the investigator according to RECIST v1.1. Confirmed ORR was defined as the proportion of patients with measurable disease at baseline with CR or PR on two consecutive tumor assessments at least 4 weeks apart, while the best response did not require a confirmatory assessment. PFS was defined as the time from first treatment to the first occurrence of disease progression or death from any cause during the study (whichever occurred first).

### Pharmacokinetics

Pharmacokinetic parameters were estimated using non-compartmental analysis of concentration–time data. Plasma samples were evaluated for divarasib using a validated liquid chromatography tandem mass spectrometry assay. The lower and upper limit of quantification for divarasib was 5 ng ml^−1^ and 5,000 ng ml^−1^, respectively. A stable labeled internal standard was used. Pharmacokinetic non-compartmental analysis was performed with nominal time using Phoenix WinNonlin (Certara USA, version 8.3). Graphical visualization was done using R (version 4.2.0; R Foundation for Statistical Computing). Pharmacokinetic samples at steady state were collected before dose, and at 0.5, 1, 2, 3, 4 and 8 h after dose on cycle 1 day 8 (C1D8) or on cycle 2 day 1 (C2D1). C1D8 or C2D1 AUC_0–24_ calculations included imputation of the 24-h time point based on pre-dose C1D8 or C2D1 plasma concentration of divarasib.

### Statistical analysis

The study planned to enroll approximately 29 patients in the cetuximab combination cohorts. This study was intended to obtain preliminary safety, pharmacokinetic and antitumor activity information and the sample sizes do not reflect any power and type I considerations. The data cutoff date was 01 April 2023 with an enrollment cutoff of 07 October 2022. This analysis included all patients who received at least one dose of divarasib and cetuximab. The response rate was reported for patients with measurable disease at baseline and summarized with 95% CIs calculated using the Clopper–Pearson method. The time-to-event end points, including time to response, duration of response and PFS, were reported descriptively and were summarized using the Kaplan–Meier method; median estimates were reported with 95% CIs.

### Reporting summary

Further information on research design is available in the Nature Portfolio Reporting Summary linked to this article.

## Online content

Any methods, additional references, Nature Portfolio reporting summaries, source data, extended data, supplementary information, acknowledgements, peer review information; details of author contributions and competing interests; and statements of data and code availability are available at 10.1038/s41591-023-02696-8.

### Supplementary information

Supplementary Information GO42144 investigators and study group, Supplementary Table 1 and clinical trial protocol

Reporting Summary

## Figures and Tables

**Extended Data Table 1:**
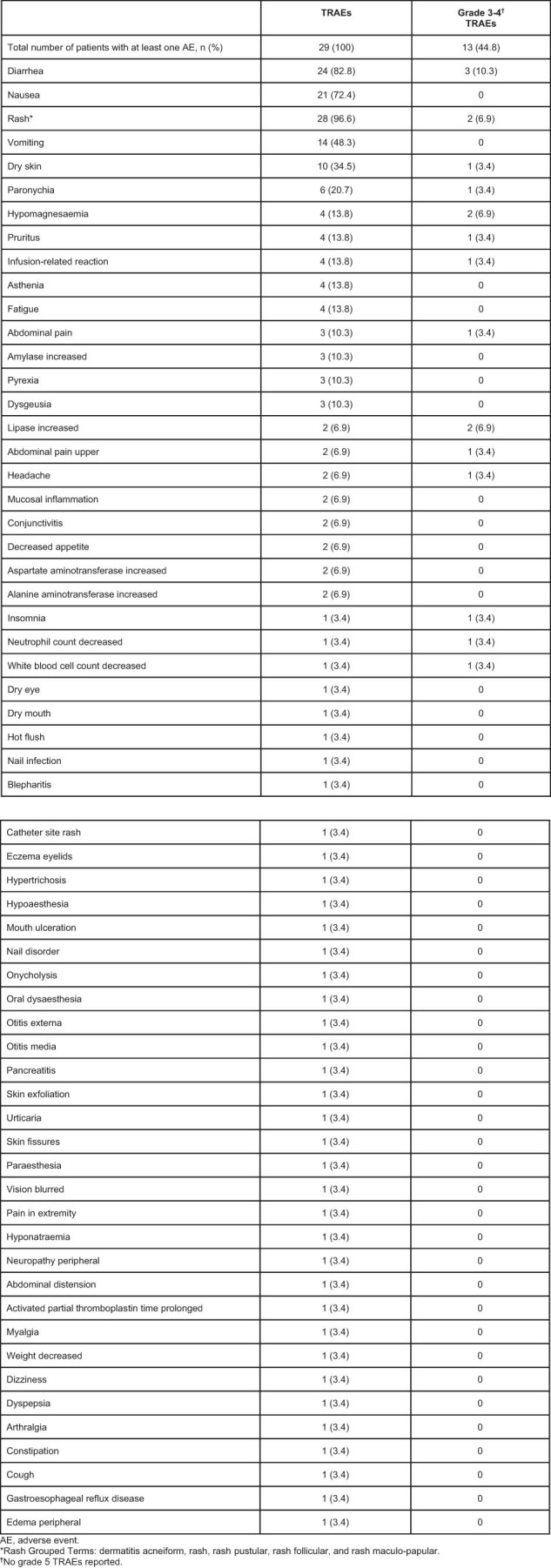
All TRAEs

**Extended Data Table 2:**
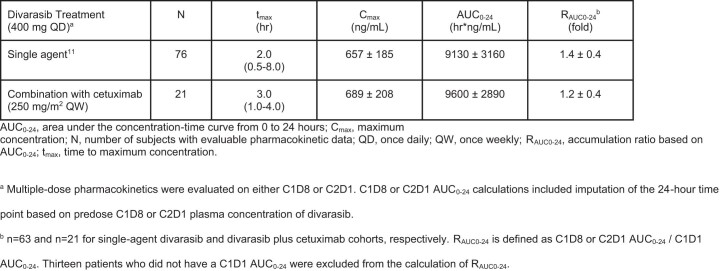
Pharmacokinetic parameters of divarasib in combination with cetuximab following multiple doses

Pharmacokinetic data from capsule and tablet formulations were pooled for this analysis. Analysis was performed with nominal time. Tmax is presented as median (range). All other statistics are presented as the mean ± s.d. Concentration values below the lower limit of quantification (BLLQ) before administration of the first dose were set to 0 and concentration values (BLLQ) after administration of the first dose administration were set to half the lower limit of quantification. Participants with dose reductions/interruptions were excluded from steady-state pharmacokinetic parameter analyses.

## Data Availability

*KRAS G12C* VAF data are available in the Supplementary Information. For eligible studies, qualified researchers may request access to individual patient-level clinical data through a data request platform. At the time of writing, this request platform is Vivli (https://vivli.org/ourmember/roche/). As this study is ongoing, access to patient-level data from this trial will not be available until at least 18 months after the last patient visit and a clinical study report has been completed. After that time, requests for data will be assessed by an independent review panel, which decides whether the data will be provided. On average, it takes a few months to access data in the Vivli platform, but the timeline will vary depending on the number of data contributors, the number of studies and your availability to respond to comments. Once approved, the data are available for up to 24 months. For up-to-date details on Roche’s Global Policy on the Sharing of Clinical Information and how to request access to related clinical study documents, see https://www.roche.com/innovation/process/clinical-trials/data-sharing/. Anonymized records for individual patients across more than one data source external to Roche cannot, and should not, be linked due to a potential increase in risk of patient reidentification.
