# Supplementary material for: Divarasib plus cetuximab in KRAS G12C-positive colorectal cancer: a phase 1b trial
Source: Nat Med. 2023 Dec 5;30(1):271–8. doi: 10.1038/s41591-023-02696-8 (PMC10803265; doi:10.1038/s41591-023-02696-8)
Supplement: Supplementary file 1 — GO42144 investigators and study group, Supplementary Table 1 and clinical trial protocol [file 41591_2023_2696_MOESM1_ESM.pdf]

nature medicine

Article https://doi.org/10.1038/s41591-023-02696-8

Divarasib plus cetuximab in *KRAS G12C*-positive colorectal cancer: a phase 1b trial

In the format provided by the authors and unedited

SUPPLEMENTARY INFORMATION

GO42144 Investigators and Study Group

COUNTRY	INVESTIGATOR	SITE
Australia	Samantha Bowyer	Linear Clinical Research Limited
Australia	Rasha Cosman	St Vincent's Hospital Sydney
Australia	Jayesh Desai	Peter MacCallum Cancer Centre
Australia	Ben Markman	Alfred Health
Belgium	Pierre Freres	CHU de Liège
Belgium	Marc Lambrechts	AZ Sint-Maarten
Belgium	Hans Prenen	UZ Antwerpen
Brazil	Carlos Barrios	Hospital Sao Lucas Da Pontificia Universidade Catolica Do Rio Grande Do Sul (PUCRS)
Brazil	Joao Daniel Cardoso Guedes	Fundacao Faculdade Regional de Medicina de Sao Jose Do Rio Preto Hospital de Base
Brazil	Sergio De Azevedo	Hospital de Clinicas de Porto Alegre (HCPA)
Brazil	Rita De Cassia Costamilan	Universidade de Caxias do Sul
Brazil	Josiane Dias	Hospital de Cancer de Barretos
Brazil	Carolina Gomes Jacobina Silva	Santa Casa de Misericórdia de Belo Horizonte
Brazil	Tabatha Nakakogue Dallagnol	Hospital Erasto Gaertner
Brazil	Victor Santos	Instituto Nacional de Câncer
Canada	Scott Laurie	Ottawa Hospital
Canada	Wilson Miller	Jewish General Hospital
Canada	Adrian Sacher	Princess Margaret Cancer Centre
Hungary	István Láng	Clinexpert Kft. - Gyöngyös
Hungary	Istvan Takacs	Semmelweis Egyetem
Israel	Ravit Geva	Tel Aviv Sourasky Medical Center
Israel	Ruth Perets	Rambam Medical Center
Israel	Einat Shacham-Shmueli	Sheba Medical Center

Italy	Chiara Cremolini	Azienda Ospedaliero Universitaria Pisana
Italy	Gianluca Del Conte	Ospedale San Raffaele S.r.l.
Italy	Alessandro Passardi	Istituto Romagnolo per lo Studio dei Tumori "Dino Amadori"
Italy	Armando Santoro	Istituto Clinico Humanitas
Italy	Salvatore Siena	ASST Grande Ospedale Metropolitano Niguarda - Presidio Ospedaliero Ospedale Niguarda
Kenya	Mansoor Saleh	Aga Khan University Hospital
Korea, Republic of	Myung-Ju Ahn	Samsung Medical Center
Korea, Republic of	Sae-Won Han	Seoul National University Hospital
Korea, Republic of	Tae Won Kim	Asan Medical Center
Korea, Republic of	Se-Hyun Kim	Seoul National University Bundang Hospital
Netherlands	Hans Gelderblom	Leids Universitair Medisch Centrum
Netherlands	Eelke Gort	Universitair Medisch Centrum Utrecht Cancer Center
Netherlands	Loes Latten-Jansen	Maastricht University Medical Center
Netherlands	Marloes Van Dongen	Het Nederlands Kanker Instituut Antoni Van Leeuwenhoek Ziekenhuis
New Zealand	Sanjeev Deva	Auckland City Hospital
New Zealand	Chris Wynne	New Zealand Clinical Research - Christchurch
Norway	Øystein Fløtten	Haukeland University Hospital
Norway	Tormod Guren	Oslo university hospital Radiumhospitalet
Poland	Rafal Dziadziuszko	Uniwersyteckie Centrum Kliniczne, Osrodek Badan Klinicznych Wczesnych Faz
Poland	Jacek Mackiewicz	Szpital Kliniczny im. Heliodora Swiecickiego w Poznaniu
Poland	Rafal Stec	Biokinetica, Przychodnia Jozefow
Russian Federation	Rustem Safin	Republican Clinical Oncology Dispensary of Ministry of Healthcare of Tatarstan Republic
Spain	Guzman Alonso	Hospital Universitario Vall d'Hebron
Spain	Andres Cervantes Ruizperez	Hospital Clinico Universitario de Valencia

Spain	Maria De Miguel Luken	START Madrid Hospital Universitario HM Sanchinarro
Spain	Alejandro Falcon	Hospital Universitario Virgen del Rocio
Spain	Laura Medina	Hospital Universitario Virgen de la Victoria
Spain	Victor Moreno	START Madrid Hospital Universitario Fundacion Jimenez Diaz
Spain	Luis Paz-Ares Rodriguez	Hospital Universitario 12 de Octubre
Switzerland	Christian Britschgi	Universitätsspital Zürich
Switzerland	Eugenio Fernandez	Hôpitaux Universitaires de Genève
Switzerland	Simon Häfliger	Universitätsspital Bern - Inselspital
Switzerland	Heinz Läubli	Universitätsspital Basel
United Kingdom	Martin Forster	University College London Hospitals
United Kingdom	Robert Jones	Velindre Cancer Centre
United Kingdom	Matthew Krebs	The Christie NHS Foundation Trust
United Kingdom	Gary Middleton	Queen Elizabeth Hospital
United States	Kathryn Arbour	Memorial Sloan Kettering Cancer Center - David H. Koch Center for Cancer Care
United States	Lyudmila Bazhenova	UC San Diego Moores Cancer Center
United States	Timothy Burns	UPMC Hillman Cancer Center
United States	Thomas Karasic	Abramson Cancer Center of The University of Pennsylvania
United States	Patricia LoRusso	Yale University
United States	Jia Luo	Dana Farber Cancer Institute
United States	Erminia Massarelli	City of Hope - Comprehensive Cancer Center
United States	Pamela Munster	University of California San Francisco
United States	Sai-Hong Ou	UCI Health Chao Family Comprehensive Cancer Center
United States	Manish Patel	Florida Cancer Specialists
United States	Susanna Ulahannan	University of Oklahoma Peggy and Charles Stephenson Cancer Center

GO42144 STUDY GROUP		
United States	Junko Aimi<br>Julie Chang<br>Yoonha Choi<br>Neekesh V. Dharia<br>Xuefeng Hou<br>Tomi Jun<br>Mark T. Lin<br>Sandhya Mandlekar<br>Huy X. Ngo<br>Nina Qi<br>Stephanie Royer-Joo<br>Jennifer L. Schutzman<br>Zhen Shi<br>Julia Suchomel<br>Kenneth K. Yau	Genentech, Inc.

Supplementary Table 1. ***KRAS G12C*** **Variant Allele Frequency (VAF) for Figure 4A**

Patient ID	Best Overall Response	Prior KRAS G12C Inhibitor Experience	Time Point	KRAS G12C VAF
19	SD	No	C1D1	1.57
19	SD	No	C1D15	0.04
19	SD	No	C3D1	0.02
26	PR	No	C1D1	34.90
26	PR	No	C1D15	0.67
26	PR	No	C3D1	0.02
6	PR	No	C1D1	0.34
6	PR	No	C1D15	0.03
6	PR	No	C3D1	0.02
5	PR	No	C1D1	12.80
5	PR	No	C1D15	0.05
5	PR	No	C3D1	0.03
10	SD	No	C1D1	0.68
10	SD	No	C1D15	0.03
10	SD	No	C3D1	0.03
21	PR	Yes	C1D1	1.51
21	PR	Yes	C1D15	0.04
21	PR	Yes	C3D1	0.03
28	PR	No	C1D1	0.34
28	PR	No	C1D15	0.03
28	PR	No	C3D1	0.04
11	PR	Yes	C1D1	1.58
11	PR	Yes	C1D15	0.09
11	PR	Yes	C3D1	0.05
22	PR	No	C1D1	23.70
22	PR	No	C1D15	0.66
22	PR	No	C3D1	0.05
8	CR	No	C1D1	0.33
8	CR	No	C1D15	0.04
8	CR	No	C3D1	0.06
17	PR	No	C1D1	20.30
17	PR	No	C1D15	0.36
17	PR	No	C3D1	0.09
16	PR	No	C1D1	54.06
16	PR	No	C1D15	0.14
16	PR	No	C3D1	0.16
24	PR	No	C1D1	28.80
24	PR	No	C1D15	0.18
24	PR	No	C3D1	0.22
7	SD	No	C1D1	12.50

7	SD	No	C1D15	0.16
7	SD	No	C3D1	0.34
27	PR	No	C1D1	38.97
27	PR	No	C1D15	6.18
27	PR	No	C3D1	0.47
20	SD	Yes	C1D1	36.7
20	SD	Yes	C1D15	4.01
20	SD	Yes	C3D1	0.63
29	SD	No	C1D1	48
29	SD	No	C1D15	0.79
29	SD	No	C3D1	1.12
12	SD	No	C1D1	36.6
12	SD	No	C1D15	0.95
12	SD	No	C3D1	2.95
1	PR	Yes	C1D1	26.69
1	PR	Yes	C1D15	1.57
1	PR	Yes	C3D1	14.39
23	SD	No	C1D1	50.9
23	SD	No	C1D15	2.27
23	SD	No	C3D1	31.4

PROTOCOL AMENDMENT, VERSION 2 (CANADA): RATIONALE

Based on feedback from Health Canada, the following changes have been made to Protocol GO42144 (Canada only):

- [redacted]
- [redacted]

Substantive new information appears in italics. This amendment represents cumulative changes to the original protocol.

PROTOCOL AMENDMENT, VERSION 3: RATIONALE

Study GO42144 plans to evaluate the safety, pharmacokinetics, and preliminary activity of GDC-6036 in combination with other anti-cancer therapies. With this amendment, GDC-6036 will be tested in combination with [redacted] cetuximab, [redacted] in patients with advanced or metastatic *KRAS G12C*-positive solid tumors.

- [redacted]
- **Arm C (GDC-6036 and Cetuximab).** Arm C will inform the safety, tolerability, PK and PD effects, and preliminary anti-tumor activity of GDC-6036 in combination with cetuximab in patients with advanced or metastatic *KRAS G12C*-positive colorectal cancer (CRC) previously treated with at least one prior therapy in the metastatic setting. Approximately 12 pts in Stage I and 20 patients in Stage II will be enrolled in this arm.
- [redacted]

[redacted]

During Stage I (dose escalation), patients will be evaluated for dose-limiting toxicities (DLTs) at escalating GDC-6036 dose levels to determine the maximum tolerated dose (MTD) or maximum administered dose (MAD, if the MTD has not been identified) for GDC-6036 administered in combination with [redacted] cetuximab, [redacted]

The study objectives have been modified accordingly (Section 2), including the addition of immunogenicity objectives and endpoints for the biotherapeutic arms. New text regarding background on the combination treatments (Section 1.2.2); study design (Section 3.1); DLT criteria (Section 3.1.1.3); rationale for study design (Section 3.3); entry criteria (Section 4.1); study treatment information (Section 4.3); concomitant and

cautionary or prohibited therapies (Section 4.4); risks (Section 5.1); adverse event reporting period (Section 5.3.1); and adverse event management guidelines (Appendix 8, Appendix 9, Appendix 10, and Appendix 11) have been included. A schedule of activities has been included for the new treatment arms (Appendix 2) and the pharmacokinetic, biomarker, and ECG schedule for GDC-6036 single-agent arms has been included in a combined schedule along with the new combination arms, including immunogenicity assessments as applicable (Appendix 3). Supplementary appendices relevant to the combination treatments have been added as well (Appendix 7 and Appendix 12).

[redacted]

Furthermore, the following optional assessments have been included and will be performed for patients who consent to them:

- **Optional FDG-PET Imaging.** FDG-PET imaging may provide an early readout of response in patients receiving GDC-6036 monotherapy or combination therapy, [redacted]
- **Optional Photographs of the Skin and/or Mucous Membranes.** Ad-hoc photographs may be taken in the setting of toxicities of the skin or the mucous membranes (i.e., rash or mucositis) or in the event of skin lesions responding to study treatment (Section 4.5.12).

Moreover, based on feedback from Health Canada, the following changes have been implemented in this global protocol amendment. These changes are reflected in previous Protocol Version 2 [Canada].

- [redacted]
- [redacted]

[redacted]

Additional changes to the protocol are as follows:

- The inclusion criterion pertaining to INR and aPTT eligibility requirements for patients receiving therapeutic anticoagulation has been updated to align with recent standard practice (Section 4.1.1).
- The exclusion criterion pertaining to history of malignancy within 5 years prior to screening has been clarified to provide flexibility with regard to allowable prior low-risk disease (see Section 4.1.2).
- Cautionary language addressing risk and benefits of study treatments in the setting of the COVID-19 pandemic has been added (Section 1.3.3 and 5.1.2).
- [redacted]

Additional minor changes have been made to improve clarity and consistency. Substantive new information appears in italics. This amendment represents cumulative changes to the original protocol.

PROTOCOL AMENDMENT, VERSION 4 (United Kingdom): RATIONALE

Protocol GO42144 has been amended to update the inclusion criteria pertaining to the duration of contraceptive measures following treatment with cetuximab in the GDC-6036 in combination with cetuximab arm (Arm C), as well as to update the exclusion criteria related to the definition of the wash-out period from prior anti-cancer therapy.

The inclusion criteria pertaining to the duration of contraceptive measures following study treatment was updated to specify 2 months after the final dose of cetuximab (Section 4.1.1.1). This update provides clarity based on the United States Package Insert, since the Summary of Product Characteristics does not provide specific guidance.

In the exclusion criteria pertaining to prior anti-cancer therapy, the wash-out period from prior treatment has been updated to exclude patients treated with chemotherapy, immunotherapy, biologic therapy, or investigational therapy as anti-cancer therapy within 3 weeks or five half-lives prior to initiation of study treatment, whichever is shorter (Section 4.1.2.1). This update provides additional flexibility for enrollment of patients who received prior therapies with short half-lives.

Additional changes to the protocol are as follows:

- The description of the Cockcroft-Gault estimation was updated to remove “glomerular filtration rate,” since this was erroneous as the estimation pertains to the creatinine clearance (Section 4.1.1.1).
- The medical monitor has been updated to [redacted] from [redacted] (Section 5.4.1).

Additional minor changes have been made to improve clarity and consistency. Substantive new information appears in italics. This amendment represents cumulative changes to the original protocol.

PROTOCOL AMENDMENT, VERSION 4 (VHP): RATIONALE

Protocol GO42144, Version 4 (VHP), has been amended [redacted] to clarify an eligibility criteria regarding the requirement to offer alternate treatment options to patients, to implement an Internal Monitoring Committee (IMC), to include additional pregnancy testing following treatment discontinuation, and to update the cardiac assessment schedule.

Changes to the protocol, along with a rationale for each change, are summarized below:

- [redacted]
- [redacted]
- The starting dose of GDC-6036 in the combination arms has been updated to be based on the maximum tolerated dose (MTD) or maximum administered dose (MAD) identified in Stage I Arm A (GDC-6036 single-agent dose escalation) for clarity (Sections 3.1.1.2 and 3.1.1.5).
- The dose escalation in combination therapy arms (Stage I, Arms [redacted] C, [redacted]) section has been updated to include that the initiation of those arms may be subject to approval by appropriate regulatory agencies and/or Institutional Review Boards/Ethics Committees (Section 3.1.1.2).
- The dose-escalation sections pertaining to the single-agent arm (Section 3.1.1.4) and the combination arms (Section 3.1.1.5) have been updated to remove the potential investigation of alternate dosing schedules, which would require an amendment prior to implementation. The section pertaining to the combination arms has also been updated to clarify that alternate lower dose levels investigated would be based on the SmPC of each combination partner (Section 3.1.1.5).
- An IMC has been included in this study to ensure that appropriate patient safety oversight is maintained throughout the conduct of this study (Section 3.2).
- A benefit-risk assessment statement for each combination arm has been included for clarity and completeness (Section 3.4.3). In addition, a reference to Section 3.4.3 has been included under Section 1.3.2 (Study Rationale and Benefit−Risk Assessment) for clarity.
- The inclusion criteria pertaining to the definition of the patient populations eligible for each arm have been updated to reflect that the study doctor must offer additional approved treatment options and discuss the risks and benefits of those treatments

before informed consent to participate in this study is obtained (Sections 4.1.1.2, 4.1.1.4 , 4.1.1.5, 4.1.1.6, 4.1.1.7, and 4.1.1.8).

- [redacted]
- The maximum infusion rate for cetuximab has been updated to 5 mg/min for the initial administration and 10 mg/min for subsequent administrations to align with the cetuximab Summary of Product Characteristics (SmPC) (Section 4.3.2.3).
- [redacted]
- A clarification has been made to the QT interval criteria for study drug discontinuation (Section 5.1.6.7) for consistency with Section 4.5.7.
- The medical monitor has been updated to [redacted] from [redacted] (Section 5.4.1).
- Two tables presenting the two-sided confidence intervals for observed adverse events and for observed responses in 20 patients have been added to provide additional justification of the sample size of 20 patients for the expansion cohorts (Section 6.1.2).
- Optional interim analyses have been removed from the statistical plan because no interim analysis is planned for this Phase I study (previously Section 6.9).
- The pregnancy testing schedule has been updated to include an additional pregnancy test at Cycle 1 Day 1 and monthly pregnancy testing following study treatment discontinuation in order to address the potential risk of pregnancy for women of childbearing potential. The duration of the pregnancy testing follow-up is based on the last dose of each study drug: 1 month for GDC-6036, [redacted] and cetuximab, [redacted] (Sections 3.1 and 4.5.6, Appendix 1 and Appendix 2).
- [redacted]
- [redacted]
- The schedule for ECG assessments has been updated to include single predose 12-lead ECG collection on Day 1 of each cycle starting at Cycle 3 in order to ensure appropriate cardiac safety monitoring throughout the study (Appendix 3).
- The guidelines for the use of corticosteroids as part of premedication for cetuximab administration have been updated to reflect the most recent U.S. Package Insert under the cetuximab safety management guidelines (Appendix 9).

Additional minor changes have been made to improve clarity and consistency. Substantive new information appears in italics. This amendment represents cumulative changes to the original protocol.

PROTOCOL AMENDMENT, VERSION 5: RATIONALE

Protocol GO42144, Version 5, includes changes to the global protocol (previously Version 3) specific to this amendment along with cumulative changes to the protocol from country-specific Version 4 (United Kingdom).

Study GO42144, Version 5, plans to evaluate the safety, pharmacokinetics, and preliminary activity of GDC-6036 alone and in combination with other anti-cancer therapies. [redacted]

- [redacted]
- [redacted]
- [redacted]

An Internal Monitoring Committee (IMC) has been included in this study to ensure that appropriate patient safety oversight is maintained throughout the conduct of this study (Section 3.2).

Additional changes to the protocol, along with a rationale for each change, are summarized below:

- [redacted]

- [redacted]
- [redacted]
- The pregnancy testing schedule has been updated to include an additional pregnancy test at Day 1 of Cycle 1 and monthly pregnancy testing following study treatment discontinuation to address the potential risk of pregnancy for women of childbearing potential. The duration of the pregnancy testing follow-up is based on the final dose of each study drug: 1 month for GDC-6036, [redacted] and cetuximab; [redacted] (Sections 3.1 and 4.5.6; Appendix 1 and Appendix 2).
- The starting dose of GDC-6036 in the combination arms has been updated to be based on the maximum tolerated dose (MTD) or maximum administered dose (MAD) identified in Stage I Arm A (GDC-6036 single-agent dose escalation) for clarity (Sections 3.1.1.2 and 3.1.1.5).
- The dose escalation in combination therapy arms section has been updated to include that the initiation of those arms may be subject to approval by appropriate regulatory agencies and/or Institutional Review Boards/Ethics Committees (Section 3.1.1.2).
- The dose-escalation sections pertaining to the single-agent arm (Section 3.1.1.4) and the combination arms (Section 3.1.1.5) have been updated to remove the potential investigation of alternate dosing schedules, which would require an amendment prior to implementation. The section pertaining to the combination arms has also been updated to clarify that alternate lower dose levels investigated would be based on the E.U. Summary of Product Characteristics (SmPC) of each combination partner (Stage I, Arms [redacted] C, [redacted]) (Section 3.1.1.5).
- A benefit-risk assessment statement for each combination arm has been included for clarity and completeness (Section 3.4.3). In addition, a reference to Section 3.4.3 has been included under Section 1.3.2 (Study Rationale and Benefit–Risk Assessment) for clarity.
- The biomarker eligibility criteria for patients with [redacted] and adenocarcinoma of the colon or rectum, and the criteria for patients requiring accessible lesions were moved to the general inclusion criteria section, as it was applicable to multiple study cohorts. This information was already previously under Section 4.1.1.2 (Section 4.1.1).
- The inclusion criteria pertaining to the definition of the patient populations eligible for each arm have been updated to reflect that the study doctor must offer additional approved treatment options, and discuss the risks and benefits of those treatments before informed consent to participate in this study is obtained (Sections 4.1.1.2, 4.1.1.4 , 4.1.1.5, 4.1.1.6, 4.1.1.7, and 4.1.1.8).
- [redacted]

- The maximum infusion rate for cetuximab has been updated to 5 mg/min for the initial administration and 10 mg/min for subsequent administrations to align with the cetuximab E.U. SmPC (Section 4.3.2.3).
- Language has been added to indicate that study sites can confirm that appropriate temperature conditions have been maintained during IMP transit either by time monitoring (shipment arrival date and time) or temperature monitoring (Section 4.3.3).
- Medications given with precaution (Section 4.4.2.1) and potential drug-drug interactions (Section 5.1.1.4) have been updated to include co-administered P-glycoprotein (P-gp) and breast cancer resistance protein (BCRP) substrates given nonclinical data.
- [redacted]
- [redacted]
- [redacted]
- [redacted]
- [redacted]
- [redacted]
- A clarification has been made to the QT interval criteria for study drug discontinuation (Section 5.1.6.7) for consistency with Section 4.5.7.
- [redacted]
- Language has been added to clarify that adverse events associated with a special situation that also qualify as adverse events of special interest should be reported within 24 hours (Section 5.3.5.11).
- The Medical Monitor has been updated to [redacted] from [redacted] (Section 5.4.1).

- Language has been added to indicate that the Informed Consent Form will instruct female patients to inform the investigator if they become pregnant (Section 5.4.3.1).
- Language regarding investigator reporting of pregnancies has been clarified (Section 5.4.3.2).
- Two tables presenting the two-sided confidence intervals for observed adverse events and for observed responses in 20 patients have been added to provide additional justification of the sample size of 20 patients for the expansion cohorts (Section 6.1.2).
- The Determination of Sample Size section for Stage II has been updated for completeness to include the statistical methodology that was used (Section 6.1.2).
- The Activity Analyses section has been updated for completeness to include the statistical methodologies that will be used for the assessments of objective response rate, duration of response (DOR), progression-free survival (PFS), 12-month DOR rate, and 12-month PFS rate (when applicable), as well as the definition of PFS (Section 6.7).
- Optional interim analyses have been removed from the statistical plan because no interim analysis is planned for this Phase I study (previously Section 6.9).
- The name of a Roche policy on data sharing has been corrected (Section 9.5).
- [redacted]
- The schedule for ECG assessments has been updated to include single predose 12-lead ECG collection on Day 1 of each cycle starting at Cycle 3 in order to ensure appropriate cardiac safety monitoring throughout the study (Appendix 3).
- [redacted]
- The guidelines for the use of corticosteroids as part of premedication for cetuximab administration have been updated to reflect the most recent U.S. Package Insert under the cetuximab safety management guidelines (Appendix 9).

Protocol GO42144, Version 4 (United Kingdom) was amended based on feedback from the Medicines and Healthcare Products Regulatory Agency (MHRA).

With this amendment, the inclusion criterion was updated pertaining to the duration of contraceptive measures following treatment with cetuximab in the GDC-6036 in combination with cetuximab arm (Arm C), as well as the exclusion criteria related to the definition of the wash-out period from prior anti-cancer therapy.

The inclusion criterion pertaining to the duration of contraceptive measures following study treatment was updated to specify 2 months after the final dose of cetuximab (Section 4.1.1.1). This update provides clarity based on the U.S. Package Insert, since the E.U. SmPC does not provide specific guidance.

In the exclusion criteria pertaining to prior anti-cancer therapy, the wash-out period from prior treatment was updated to exclude patients treated with chemotherapy, immunotherapy, biologic therapy, or investigational therapy as anti-cancer therapy within 3 weeks or five half-lives prior to initiation of study treatment, whichever is shorter (Section 4.1.2.1). This update provides additional flexibility for enrollment of patients who received prior therapies with short half-lives.

Additional changes to the protocol were as follows:

- The description of the Cockcroft-Gault estimation was updated to remove "glomerular filtration rate," since this was erroneous as the estimation pertains to the creatinine clearance (Section 4.1.1.1).

Additional minor changes have been made to improve clarity and consistency. Substantive new information appears in italics. This amendment represents cumulative changes to the original protocol.

PROTOCOL AMENDMENT, VERSION 5 (VHP): RATIONALE

Protocol GO42144, Version 5 (VHP), has been amended [redacted] and to clarify the statistical methodologies used for the sample size determination and activity analyses.

Changes to the protocol, along with a rationale for each change, are summarized below:

- [redacted]
- The Determination of Sample Size section for Stage II has been updated for completeness to include the statistical methodology that was used (Section 6.1.2)
- The Activity Analyses section has been updated for completeness to include the statistical methodologies that will be used for the assessments of objective response rate, duration of response (DOR), progression-free survival (PFS), and 12-month DOR rate, and 12-month PFS rate (when applicable), as well as the definition of PFS (Section 6.7).

[redacted]

Changes to the protocol, along with a rationale for each change, are summarized below:

- [redacted]
- [redacted]

- The starting dose of GDC-6036 in the combination arms has been updated to be based on the maximum tolerated dose (MTD) or maximum administered dose (MAD) identified in Stage I Arm A (GDC-6036 single-agent dose escalation) for clarity (Sections 3.1.1.2 and 3.1.1.5).
- The dose escalation in combination therapy arms (Stage I, Arms [redacted] C, [redacted]) section has been updated to include that the initiation of those arms may be subject to approval by appropriate regulatory agencies and/or Institutional Review Boards/Ethics Committees (Section 3.1.1.2).
- The dose-escalation sections pertaining to the [redacted] and the combination arms (Section 3.1.1.5) have been updated to remove the potential investigation of alternate dosing schedules, which would require an amendment prior to implementation. The section pertaining to the combination arms has also been updated to clarify that alternate lower dose levels investigated would be based on the SmPC of each combination partner (Section 3.1.1.5).
- An IMC has been included in this study to ensure that appropriate patient safety oversight is maintained throughout the conduct of this study (Section 3.2).
- A benefit-risk assessment statement for each combination arm has been included for clarity and completeness (Section 3.4.3). In addition, a reference to Section 3.4.3 has been included under Section 1.3.2 (Study Rationale and Benefit–Risk Assessment) for clarity.
- The inclusion criteria pertaining to the definition of the patient populations eligible for each arm have been updated to reflect that the study doctor must offer additional approved treatment options and discuss the risks and benefits of those treatments before informed consent to participate in this study is obtained (Sections 4.1.1.2, 4.1.1.4 , 4.1.1.5, 4.1.1.6, 4.1.1.7, and 4.1.1.8).
- The maximum GDC-6036 dose to be administered in this study has been clarified as 1 gram daily, on the basis of predicted pharmacokinetics and pill burden (Section 4.3.2.1).
- The maximum infusion rate for cetuximab has been updated to 5 mg/min for the initial administration and 10 mg/min for subsequent administrations to align with the cetuximab Summary of Product Characteristics (SmPC) (Section 4.3.2.3).
- [redacted]
- A clarification has been made to the QT interval criteria for study drug discontinuation (Section 5.1.6.7) for consistency with Section 4.5.7.
- The medical monitor has been updated to [redacted] from [redacted] (Section 5.4.1).
- Two tables presenting the two-sided confidence intervals for observed adverse events and for observed responses in 20 patients have been added to provide additional justification of the sample size of 20 patients for the expansion cohorts (Section 6.1.2).

- Optional interim analyses have been removed from the statistical plan because no interim analysis is planned for this Phase I study (previously Section 6.9).
- The pregnancy testing schedule has been updated to include an additional pregnancy test at Cycle 1 Day 1 and monthly pregnancy testing following study treatment discontinuation in order to address the potential risk of pregnancy for women of childbearing potential. The duration of the pregnancy testing follow-up is based on the last dose of each study drug: 1 month for GDC-6036, [redacted] and cetuximab, [redacted] (Sections 3.1 and 4.5.6; Appendix 1 and Appendix 2).
- [redacted]
- [redacted]
- The schedule for ECG assessments has been updated to include single predose 12-lead ECG collection on Day 1 of each cycle starting at Cycle 3 in order to ensure appropriate cardiac safety monitoring throughout the study (Appendix 3).
- The guidelines for the use of corticosteroids as part of premedication for cetuximab administration have been updated to reflect the most recent U.S. Package Insert under the cetuximab safety management guidelines (Appendix 9).

Additional minor changes have been made to improve clarity and consistency. Substantive new information appears in italics. This amendment represents cumulative changes to the original protocol.

PROTOCOL AMENDMENT, VERSION 6 (EUROPE): RATIONALE

Protocol GO42144, Version 6 (Europe), replaces Protocol GO42144, Version 5 (VHP).

Study GO42144 evaluates the safety, pharmacokinetics, and preliminary activity of GDC-6036 alone and in combination with other anti-cancer therapies. [redacted]

- [redacted]
- [redacted]
- [redacted]

Additional changes to the protocol, along with a rationale for each change, are summarized below:

- [redacted]
- [redacted]

- [redacted]
- The inclusion criterion pertaining to the duration of contraceptive measures following study treatment has been updated to specify two months after the final dose of cetuximab (Section 4.1.1.1). This update provides clarity based on the U.S. Package Insert, since the E.U. SmPC does not provide specific guidance.
- The biomarker eligibility criteria for patients with [redacted] and adenocarcinoma of the colon or rectum, and the criteria for patients requiring accessible lesions were moved to the general inclusion criteria section, as it was applicable to multiple study cohorts. This information was already previously under Section 4.1.1.2 (Section 4.1.1).
- The description of the Cockcroft-Gault estimation has been updated to remove "glomerular filtration rate," since this was erroneous as the estimation pertains to the creatinine clearance (Section 4.1.1.1).
- In the exclusion criteria pertaining to prior anti-cancer therapy, the wash-out period from prior treatment has been updated to exclude patients treated with chemotherapy, immunotherapy, biologic therapy, or investigational therapy as anti-cancer therapy within 3 weeks or five half-lives prior to initiation of study treatment, whichever is shorter (Section 4.1.2.1). This update provides additional flexibility for enrollment of patients who received prior therapies with short half-lives.
- Language has been added to indicate that study sites can confirm that appropriate temperature conditions have been maintained during IMP transit either by time monitoring (shipment arrival date and time) or temperature monitoring (Section 4.3.3).
- Medications given with precaution (Section 4.4.2.1) and potential drug-drug interactions (Section 5.1.1.4) have been updated to include co-administered P-glycoprotein (P-gp) and breast cancer resistance protein (BCRP) substrates given nonclinical data.
- [redacted]
- [redacted]
- [redacted]
- [redacted]

- [redacted]
- Language has been added to clarify that adverse events associated with a special situation that also qualify as adverse events of special interest should be reported within 24 hours (Section 5.3.5.11).
- Language has been added to indicate that the Informed Consent Form will instruct female patients to inform the investigator if they become pregnant (Section 5.4.3.1).
- Language regarding investigator reporting of pregnancies has been clarified (Section 5.4.3.2).
- The name of a Roche policy on data sharing has been corrected (Section 9.5).
- [redacted]
- [redacted]

Additional minor changes have been made to improve clarity and consistency. Substantive new information appears in italics. This amendment represents cumulative changes to the original protocol.

PROTOCOL AMENDMENT, VERSION 7: RATIONALE

Protocol GO42144, Version 7, has been amended to include the addition of [redacted] to include additional biopsy cohorts in the combination arms with cetuximab (Arm C2), [redacted]

- [redacted]

- Benefit–risk assessment and guidance on concomitant administration of severe acute respiratory syndrome coronavirus 2 (SARS-CoV-2) vaccines has been updated to reflect emerging safety information on COVID-19 and associated treatments (Sections 1.3.3 and 4.4.1).
- Language has been added to allow the enrollment of patients into newly added Stage II dose expansion biopsy cohorts (Arm C2, [redacted]) in order to facilitate the collection of additional pharmacodynamic (PD) biomarker data for robust analyses (Sections 3.1, 3.1.2.1, 3.4.1, 4.1.1.7– 4.1.1.9, 4.1.1.13, 4.5.6; Table 1; Figure 5; and Appendices 2 and 3).

  Accordingly, language referring to treatment arms has been modified to refer to cohorts.
- Language has been added to allow patients enrolled in new Stage II biopsy cohorts (Arm C2, [redacted]) to undergo intra-patient dose escalation (Sections 3.1, 3.1.2.1, 3.1.2.2, 4.1.1.7, 4.1.1.9, 4.1.1.13, 4.1.2.3, 4.1.2.4, and 4.1.2.6).
- [redacted]
- Language has been added to allow patients experiencing clinical benefit, in the judgment of the investigator, to continue study treatment beyond disease progression (as defined by RECIST, v1.1) at the investigator's discretion, provided that certain criteria are met including evidence of clinical benefit as assessed by investigator (Sections 3.1 and 4.6.1).
- [redacted]

[redacted]

- [redacted]
- [redacted]
- The definition of childbearing potential has been updated as per the newest Roche guidelines (Section 4.1.1.1).
- The contraception requirements for men have been amended to include condom use for all men instead of men who are not surgically sterile to address recommendations by the Belgium Health Authority (Section 4.1.1.1).
- [redacted]
- [redacted]
- [redacted]
- The responsibilities of the investigator and the role of the Medical Monitor during study conduct have been clarified (Appendix 8).
- [redacted]
- [redacted]
- Freshly collected tissue requirements have been updated to include Arm C2, [redacted] (Sections 4.5.6 and 4.5.12).
- [redacted]
- [redacted]

- [redacted]
- [redacted]
- Response evaluation criteria in solid tumors, version 1.1 has been edited for clarity (Appendix 5).
- [redacted]
- [redacted]

Additional minor changes have been made to improve clarity and consistency. Substantive new information appears in italics. This amendment represents cumulative changes to the original protocol.

PROTOCOL AMENDMENT, VERSION 8 (EUROPE): RATIONALE

Protocol GO42144, Version 8, has been amended to include the addition of [redacted] to include additional biopsy cohorts in the combination arms with cetuximab (Arm C2), [redacted]

- [redacted]

- Benefit–risk assessment and guidance on concomitant administration of severe acute respiratory syndrome coronavirus 2 (SARS-CoV-2) vaccines has been updated to reflect emerging safety information on COVID-19 and associated treatments (Sections 1.3.3 and 4.4.1).
- Language has been added to allow the enrollment of patients into newly added Stage II dose expansion biopsy cohorts (Arm C2, [redacted]) in order to facilitate the collection of additional pharmacodynamic (PD) biomarker data for robust analyses (Sections 3.1, 3.1.2.1, 3.4.1, 4.1.1.7– 4.1.1.9, 4.1.1.13, 4.5.6; Table 1; Figure 5; and Appendices 2 and 3).

  Accordingly, language referring to treatment arms has been modified to refer to cohorts.
- Language has been added to allow patients enrolled in new Stage II biopsy cohorts (Arm C2, [redacted]) to undergo intra-patient dose escalation (Sections 3.1, 3.1.2.1, 3.1.2.2, 4.1.1.7, 4.1.1.9, 4.1.1.13, 4.1.2.3, 4.1.2.4, and 4.1.2.6).
- [redacted].
- Language has been added to allow patients experiencing clinical benefit, in the judgment of the investigator, to continue study treatment beyond disease progression (as defined by RECIST, v1.1) at the investigator's discretion, provided that certain criteria are met including evidence of clinical benefit as assessed by investigator (Sections 3.1 and 4.6.1).
- [redacted]

[redacted]

- [redacted]
- [redacted]
- The definition of childbearing potential has been updated as per the newest Roche guidelines (Section 4.1.1.1).
- The contraception requirements for men have been amended to include condom use for all men instead of men who are not surgically sterile to address recommendations by the Belgium Health Authority (Section 4.1.1.1).
- [redacted]
- [redacted]
- [redacted]
- The responsibilities of the investigator and the role of the Medical Monitor during study conduct have been clarified (Appendix 8).
- [redacted]

[redacted]

- Freshly collected tissue requirements have been updated to include Arm C2, [redacted] (Sections 4.5.6 and 4.5.12).
- [redacted]
- The prevalence of potential gastrointestinal risks and elevation of hepatic transaminases of other KRAS G12C inhibitors has been revised to align with updated clinical reporting (Sections 5.1.1.1 and 5.1.1.3).

- [redacted]
- [redacted]
- Response evaluation criteria in solid tumors, version 1.1 has been edited for clarity (Appendix 5).
- [redacted]
- [redacted]

Additional minor changes have been made to improve clarity and consistency. Substantive new information appears in italics. This amendment represents cumulative changes to the original protocol.

PROTOCOL AMENDMENT, VERSION 8: RATIONALE

Protocol GO42144, Version 8, has been amended [redacted] These updates include:

- [redacted]
- The planned enrollment to select dose-expansion combination arms (Stage II Arm C1 [redacted]) has been updated from n = 20 to n = 20–40 patients based upon emerging safety, PK, pharmacodynamic, and anti-tumor activity data to enable further characterization of the safety and anti-tumor activity of these combinations at one or more dose level(s) that meet the criteria for dose expansion and/or in the context of the evolving treatment landscape for *KRAS G12C*–positive cancers (Sections 3.1, 3.1.2, 4.1, and 6.1.2).
- [redacted]

In addition, this amendment includes the following updated information:

- The email address for withdrawal from the Research Biosample Repository after site closure has been updated (Section 4.5.16.6).
- The timing for the measurement of the baseline QT interval value for the management of increases in QT interval has been clarified (Section 5.1.8.9).
- The URL address for the dissemination of clinical study data has been updated (Section 9.5).

Additional minor changes have been made to improve clarity and consistency. Substantive new information appears in italics. This amendment represents cumulative changes to the original protocol.

PROTOCOL AMENDMENT, VERSION 9 (EUROPE): RATIONALE

Protocol GO42144, Version 9, has been amended [redacted] These updates include:

- [redacted]
- The planned enrollment to select dose-expansion combination arms (Stage II Arm C1, [redacted]) has been updated from n = 20 to n = 20–40 patients based upon emerging safety, PK, pharmacodynamic, and anti-tumor activity data to enable further characterization of the safety and anti-tumor activity of these combinations at one or more dose level(s) that meet the criteria for dose expansion and/or in the context of the evolving treatment landscape for *KRAS G12C*–positive cancers (Sections 3.1, 3.1.2, 4.1, and 6.1.2).
- [redacted]

In addition, this amendment includes the following updated information:

- The email address for withdrawal from the Research Biosample Repository after site closure has been updated (Section 4.5.16.6).
- The timing for the measurement of the baseline QT interval value for the management of increases in QT interval has been clarified (Section 5.1.8.9).
- The URL address for the dissemination of clinical study data has been updated (Section 9.5).

Additional minor changes have been made to improve clarity and consistency. Substantive new information appears in italics. This amendment represents cumulative changes to the original protocol.

PROTOCOL

**TITLE:**	**A PHASE Ia/Ib DOSE-ESCALATION AND DOSE-EXPANSION STUDY EVALUATING THE SAFETY, PHARMACOKINETICS, AND ACTIVITY OF GDC-6036 AS A SINGLE AGENT AND IN COMBINATION WITH OTHER ANTI-CANCER THERAPIES IN PATIENTS WITH ADVANCED OR METASTATIC SOLID TUMORS WITH A *KRAS G12C* MUTATION**
**PROTOCOL NUMBER:**	GO42144
**VERSION NUMBER:**	5
**EUDRACT NUMBER:**	[redacted]
**IND NUMBER:**	[redacted]
**NCT NUMBER:**	NCT04449874
**TEST PRODUCTS:**	GDC-6036 (RO7435846), [redacted] Cetuximab, [redacted]
**MEDICAL MONITOR:**	[redacted]
**SPONSOR:**	Genentech, Inc.
**APPROVAL DATE:**	See electronic date stamp below.

PROTOCOL AMENDMENT APPROVAL

Date and Time (UTC)	Title	Approver's Name
29-Apr-2021 23:16:38	Company Signatory	[redacted]

CONFIDENTIAL

The information contained in this document, especially any unpublished data, is the property of Genentech, Inc. (or under its control) and therefore is provided to you in confidence as an investigator, potential investigator, or consultant, for review by you, your staff, and an applicable Ethics Committee or Institutional Review Board. It is understood that this information will not be disclosed to others without written authorization from Genentech except to the extent necessary to obtain informed consent from persons to whom the drug may be administered.

TABLE OF CONTENTS

PROTOCOL ACCEPTANCE FORM 20

PROTOCOL SYNOPSIS 21

1. BACKGROUND 46

1.1 Background on KRAS G12C and Cancer 46

1.2 Background on Study Treatments 47

1.2.1 GDC-6036 47

1.2.2 Combination Treatments 49

[redacted]

1.2.2.2 Cetuximab 49

[redacted]

1.3 Study Rationale and Benefit–Risk Assessment 51

1.3.1 GDC-6036 as a Single Agent 51

1.3.2 GDC-6036 in Combination Therapy 53

1.3.3 Treatment in the Setting of the COVID-19 Pandemic 53

2. OBJECTIVES AND ENDPOINTS 54

2.1 Safety Objective (Primary Study Objective) 55

2.2 Pharmacokinetic Objectives 55

[redacted]

2.4 Activity Objectives 56

2.5 Biomarker Objective 56

2.6 Additional Objective 57

3. STUDY DESIGN 57

3.1 Description of the Study 57

3.1.1 Dose-Escalation Stage (Stage I) 63

3.1.1.1 Dose Escalation of Single-Agent GDC-6036 (Arm A) 63

3.1.1.2 Dose-Escalation in Combination Therapy Arms (Stage I, Arms [redacted] C, [redacted]) 64
3.1.1.3 Definition of Dose-Limiting Toxicity 65
[redacted]
3.1.1.5 Dose-Escalation Rules: Stage I, [redacted] Arm C, [redacted] 68
3.1.1.6 Intra-Patient Dose Escalation 69
[redacted]
3.1.2 Dose-Expansion Stage (Stage II) 71
3.1.2.1 General Description 71
[redacted]
3.2 Internal Monitoring Committee 74
3.3 End of Study and Length of Study 75
3.4 Rationale for Study Design 75
3.4.1 Rationale for Patient Population 75
[redacted]
3.4.3 Rationale for Combining GDC-6036 with Other Anti-Cancer Therapies 79
[redacted]
3.4.3.2 Rationale for Combination Therapy with EGFR Inhibitors 80
[redacted]

[redacted] [redacted]
3.4.6 Rationale for Dosing beyond Cycle 1 84
3.4.7 Rationale for PK and [redacted] Sampling Schedule 84
3.4.8 Rationale for Biomarker Assessments.................................. 85
3.4.8.1 KRAS G12C Mutation Status from Tissue and Circulating Tumor DNA Assessments.................................. 86
3.4.8.2 Pharmacodynamic Pathway Modulation................................ 86
3.4.8.3 Sequencing of Genes Related to Resistance to GDC-6036 87
3.4.8.4 Protein, RNA, and DNA Analysis 87
3.4.8.5 Plasma Sample for Somatic Tumor Mutation Analysis and Other Biomarkers 88
3.4.8.6 Blood Sample for Next-Generation Sequencing 88
3.4.8.7 Optional Tumor Biopsy Sample at the Time of Disease Progression 88
3.4.8.8 Tumor Biopsy Sample for Patients Previously Treated with a KRAS G12C Inhibitor (Arms C, [redacted] [redacted]) 88
3.4.9 Rationale for QT/QTc Assessments 89
3.4.10 Rationale for Optional FDG-PET Imaging.............................. 90
[redacted] [redacted]
4. MATERIALS AND METHODS 90
4.1 Patients... 90
4.1.1 Inclusion Criteria 90
4.1.1.1 General Inclusion Criteria 91
[redacted] [redacted]

[redacted]

4.1.1.6 Stage I and Stage II Arm C (Cetuximab Combination) 96

[redacted]

4.1.2 Exclusion Criteria 100

4.1.2.1 General Exclusion Criteria 100

[redacted]

4.1.2.3 Stage I and Stage II Arm C (Cetuximab Combination) 104

[redacted]

4.2 Method of Treatment Assignment 105

4.3 Study Treatment and Other Treatments Relevant to the Study Design 105

4.3.1 Study Treatment Formulation, Packaging, and Handling 105

4.3.1.1 GDC-6036 105

[redacted]

4.3.1.3 Cetuximab 106

[redacted]

[redacted]
4.3.2 Study Treatment Dosage, Administration, and Compliance 106
4.3.2.1 GDC-6036 106
[redacted]
4.3.2.3 Cetuximab 113
[redacted]
4.3.3 Investigational Medicinal Product Handling and Accountability 116
4.3.4 Continued Access to GDC-6036 [redacted] 117
4.4 Concomitant Therapy, Prohibited Food, and Additional Restrictions 118
4.4.1 Permitted Therapy 118
4.4.2 Cautionary Therapy 118
4.4.2.1 Medications Given with Precaution due to Effects Related to GDC-6036 118
[redacted]
4.4.2.3 Medications Given with Precaution in Arm C (GDC-6036 in Combination with Cetuximab) 120
[redacted]
4.4.2.7 Herbal Therapies 121
4.4.3 Prohibited Therapy 121
4.4.3.1 Prohibited Therapy for All Patients 121
[redacted]
4.4.4 Prohibited Food 123

4.5.4.5 Additional Restrictions 123
4.5 Study Assessments 123
4.5.1 Informed Consent Forms and Screening Log 124
4.5.2 Medical History, Baseline Conditions, Concomitant Medication, and Demographic Data 124
4.5.3 Physical Examinations 124
4.5.4 Vital Signs 125
4.5.5 Tumor and Response Evaluations 125
4.5.6 Laboratory, Biomarker, and Other Biological Samples 125
4.5.7 Electrocardiograms 129
[redacted]
4.5.10 Blood Samples for Whole Genome Sequencing or Whole Exome Sequencing (Patients at Participating Sites) 131
4.5.11 Pre-Treatment Tumor Tissue 132
[redacted]
4.5.13 Optional Tumor Biopsy at the Time of Disease Progression 134
4.5.14 Optional Saliva and Hair Follicle Samples for Exploratory Biomarker Research 135
4.5.15 Optional FDG-PET Imaging 135
4.5.16 Collection of Photographs 136
4.5.17 Optional Samples for Research Biosample Repository 136
4.5.17.1 Overview of the Research Biosample Repository 136
4.5.17.2 Approval by the Institutional Review Board or Ethics Committee 137
4.5.17.3 Sample Collection 137
4.5.17.4 Confidentiality 137
4.5.17.5 Consent to Participate in the Research Biosample Repository 138

4.5.17.6 Withdrawal from the Research Biosample Repository 138
4.5.17.7 Monitoring and Oversight 139
4.6 Treatment, Patient, Study, and Site Discontinuation 139
4.6.1 Study Treatment Discontinuation 139
4.6.2 Patient Discontinuation from the Study 140
4.6.3 Study Discontinuation 140
4.6.4 Site Discontinuation 140
5. ASSESSMENT OF SAFETY 141
5.1 Safety Plan 141
5.1.1 Risks Associated with GDC-6036 142
5.1.1.1 Potential Gastrointestinal Toxicities (Diarrhea, Nausea, and Vomiting) 142
5.1.1.2 Potential Oral Mucosal Irritation 143
5.1.1.3 Potential Elevation of Hepatic Transaminases 143
5.1.1.4 Potential Drug-Drug Interactions 143
[redacted]
5.1.3 Risks Associated with Cetuximab 145
[redacted]

5.1.7 Management of Patients Who Experience Adverse Events 150

5.1.7.1 Dose Modifications and Treatment Interruption 151

5.1.7.2 Management Guidelines 152

[redacted]

5.1.7.4 Management of Patients Who Experience Specific Adverse Events with Cetuximab 154

[redacted]

5.1.7.8 Management of Increases in QT Interval 155

5.2 Safety Parameters and Definitions 156

5.2.1 Adverse Events 156

5.2.2 Serious Adverse Events (Immediately Reportable to the Sponsor) 156

5.2.3 Adverse Events of Special Interest (Immediately Reportable to the Sponsor) 157

5.2.3.1 Adverse Events of Special Interest for GDC-6036 (All Arms) 157

5.2.3.2 Adverse Events of Special Interest for Combination Treatments 158

5.2.4 Dose-Limiting Toxicities (Immediately Reportable to the Sponsor) 159

5.3 Methods and Timing for Capturing and Assessing Safety Parameters 159

5.3.1 Adverse Event Reporting Period 159

5.3.2 Eliciting Adverse Event Information 160

5.3.3 Assessment of Severity of Adverse Events 160

5.3.4 Assessment of Causality of Adverse Events 161

5.3.5 Procedures for Recording Adverse Events 161

5.3.5.1 Diagnosis versus Signs and Symptoms 162

5.3.5.2 Adverse Events That Are Secondary to Other Events 162

5.3.5.3 Persistent or Recurrent Adverse Events 162
5.3.5.4 Abnormal Laboratory Values 163
5.3.5.5 Abnormal Vital Sign Values 163
5.3.5.6 Abnormal Liver Function Tests 164
5.3.5.7 Deaths 164
5.3.5.8 Preexisting Medical Conditions 165
5.3.5.9 Lack of Efficacy or Worsening of KRAS G12C–Positive Cancer 165
5.3.5.10 Hospitalization or Prolonged Hospitalization 165
5.3.5.11 Cases of Overdose, Medication Error, Drug Abuse, or Drug Misuse 166
5.3.5.12 Safety Biomarker Data 168
5.4 Immediate Reporting Requirements from Investigator to Sponsor 168
5.4.1 Emergency Medical Contacts 169
5.4.2 Reporting Requirements for Serious Adverse Events, Adverse Events of Special Interest, and Dose-Limiting Toxicities 169
5.4.2.1 Events That Occur prior to Study Drug Initiation 169
5.4.2.2 Events That Occur after Study Drug Initiation 169
5.4.3 Reporting Requirements for Pregnancies 170
5.4.3.1 Pregnancies in Female Patients 170
5.4.3.2 Pregnancies in Female Partners of Male Patients 170
5.4.3.3 Abortions 171
5.4.3.4 Congenital Anomalies/Birth Defects 171
5.5 Follow-Up of Patients after Adverse Events 171
5.5.1 Investigator Follow-Up 171
5.5.2 Sponsor Follow-Up 171
5.6 Adverse Events That Occur after the Adverse Event Reporting Period 172
5.7 Expedited Reporting to Health Authorities, Investigators, Institutional Review Boards, and Ethics Committees 172

6. STATISTICAL CONSIDERATIONS AND ANALYSIS PLAN 173
6.1 Determination of Sample Size 173

6.1.1 Dose Escalation (Stage I) 173
6.1.2 Dose Expansion (Stage II) 174
6.2 Summaries of Conduct of Study 175
6.3 Summaries of Demographic and Baseline Characteristics 175
6.4 Safety Analyses 175
6.5 Pharmacokinetic Analyses 176
6.6 Immunogenicity Analyses 176
6.7 Activity Analyses 177
6.7.1 Objective Response Rate 177
6.7.2 Duration of Response 177
6.7.3 Progression-Free Survival 177
6.8 Biomarker Analyses 178

7. DATA COLLECTION AND MANAGEMENT 178
7.1 Data Quality Assurance 178
7.2 Electronic Case Report Forms 179
7.3 Source Data Documentation 179
7.4 Use of Computerized Systems 180
7.5 Retention of Records 180

8. ETHICAL CONSIDERATIONS 180
8.1 Compliance with Laws and Regulations 180
8.2 Informed Consent 180
8.3 Institutional Review Board or Ethics Committee 182
8.4 Confidentiality 182
8.5 Financial Disclosure 183

9. STUDY DOCUMENTATION, MONITORING, AND ADMINISTRATION 183
9.1 Study Documentation 183
9.2 Protocol Deviations 183
9.3 Site Inspections 183
9.4 Administrative Structure 184
9.5 Dissemination of Data and Protection of Trade Secrets 184

9.6 Protocol Amendments 185

10. REFERENCES 186

LIST OF TABLES

Table 1 Study Treatment and Tumor Types by Stage and Arm 58

[redacted]

Table 3 Guidelines for Management of Patients Who Experience Adverse Events with GDC-6036 153

Table 4 Adverse Event Severity Grading Scale for Events Not Specifically Listed in NCI CTCAE 160

Table 5 Causal Attribution Guidance 161

Table 6 Probability of Observing DLTs with Different Underlying DLT Rates 173

Table 7 Probability of Safety-Signal Detection with an Expansion Cohort of 20 Patients 174

Table 8 Two-Sided Exact Confidence Intervals for Observed Adverse Events in 20 Patients 174

Table 9 Two-Sided Exact Confidence Intervals for Observed Responses in 20 Patients 175

LIST OF FIGURES

[redacted]

Figure 2 Study Schema for Stage I Combination Arms [redacted] C, [redacted] Dose Escalation 62

[redacted]

Figure 4 Study Schema for Stage II: Dose Expansion 63

Figure 5 Cycle 1 GDC-6036 Dosing, PK, and Biopsy Schedules 109

LIST OF APPENDICES

[redacted]

Appendix 2 Schedule of Activities: Stage I and Stage II for Combination Arms [redacted] C, [redacted] 197

Appendix 3 Pharmacokinetic, [redacted] Biomarker, and ECG Schedule: Stages I and II (All Arms) 204
Appendix 4 Eastern Cooperative Oncology Group Performance Status Scale 214
Appendix 5 Response Evaluation Criteria in Solid Tumors, Version 1.1 (RECIST v1.1) 215
Appendix 6 New York Heart Association Functional Classification 225
[redacted]
Appendix 9 Guidelines for Management of Patients Who Experience Adverse Events with Cetuximab 257
[redacted]

PROTOCOL ACCEPTANCE FORM

**TITLE:**	**A PHASE Ia/Ib DOSE-ESCALATION AND DOSE-EXPANSION STUDY EVALUATING THE SAFETY, PHARMACOKINETICS, AND ACTIVITY OF GDC-6036 IN PATIENTS AS A SINGLE AGENT AND IN COMBINATION WITH OTHER ANTI-CANCER THERAPIES WITH ADVANCED OR METASTATIC SOLID TUMORS WITH A *KRAS G12C* MUTATION**
**PROTOCOL NUMBER:**	GO42144
**VERSION NUMBER:**	5
**EUDRACT NUMBER:**	[redacted]
**IND NUMBER:**	[redacted]
**NCT NUMBER:**	NCT04449874
**TEST PRODUCTS:**	GDC-6036 (RO7435846), [redacted] Cetuximab, [redacted]
**MEDICAL MONITOR:**	[redacted]
**SPONSOR:**	Genentech, Inc.

I agree to conduct the study in accordance with the current protocol.

____
Principal Investigator's Name (print)

____ ____________________
Principal Investigator's Signature Date

Please return the signed original of this form to your contract research organization's representatives. Please retain a copy for your study files.

PROTOCOL SYNOPSIS

**TITLE:**	**A PHASE Ia/Ib DOSE-ESCALATION AND DOSE-EXPANSION STUDY EVALUATING THE SAFETY, PHARMACOKINETICS, AND ACTIVITY OF GDC-6036 AS A SINGLE AGENT AND IN COMBINATION WITH OTHER ANTI-CANCER THERAPIES IN PATIENTS WITH ADVANCED OR METASTATIC SOLID TUMORS WITH A *KRAS G12C* MUTATION**
**PROTOCOL NUMBER:**	GO42144
**VERSION NUMBER:**	5
**EUDRACT NUMBER:**	[redacted]
**IND NUMBER:**	[redacted]
**NCT NUMBER:**	NCT04449874
**TEST PRODUCTS:**	GDC-6036 (RO7435846), [redacted] Cetuximab, [redacted]
**PHASE:**	Ia/Ib
**INDICATION:**	Advanced or metastatic *KRAS G12C*–positive cancers
**SPONSOR:**	Genentech, Inc.

OBJECTIVES AND ENDPOINTS

This Phase Ia/Ib study will evaluate the safety, pharmacokinetics, [redacted] preliminary activity, and biomarkers of GDC-6036 as a single agent (Arm A) and in combination with other anti-cancer therapies in patients with advanced or metastatic solid tumors with a *KRAS G12C* mutation. Combination therapies will include [redacted] cetuximab (Arm C), [redacted] CRC, [redacted] Specific objectives and corresponding endpoints for the study are outlined below.

<u>Safety Objective (Primary Study Objective)</u>

The safety objective for this study is to evaluate the safety of GDC-6036 as a single agent and in combination with other anti-cancer therapies on the basis of the following endpoints:

- Incidence and severity of adverse events, with severity determined according to National Cancer Institute Common Terminology Criteria for Adverse Events, Version 5.0 (NCI CTCAE v5.0)
- Incidence and nature of dose-limiting toxicities (DLTs)
- Change from baseline in targeted vital signs
- Change from baseline in targeted clinical laboratory test results
- Change from baseline in targeted ECG parameters

Pharmacokinetic Objectives

The pharmacokinetic (PK) objectives for this study are to characterize the PK profile of GDC-6036 when administered as a single agent and in combination with other anti-cancer therapies and to characterize the PK profile of these anti-cancer therapies when administered in combination with GDC-6036, on the basis of the following endpoints:

- Plasma concentrations of GDC-6036, [redacted] at specified timepoints
- Serum concentrations of [redacted] cetuximab, and [redacted] at specified timepoints

The exploratory PK objectives for this study are as follows:

- To evaluate potential relationships between drug exposure and the safety and activity of GDC-6036 *as a single agent and in combination with other anti-cancer therapies*
- To evaluate the exposure of potential circulating metabolites of GDC-6036 following a single or repeat oral dose(s) of GDC-6036 *as a single agent or in combination with other anti-cancer therapies*
- [redacted]

[redacted]

Activity Objectives

The activity objective for this study is to make a preliminary assessment of the activity of GDC-6036 as a single agent and in combination with other anti-cancer therapies on the basis of the following endpoints:

- Objective response rate (ORR), defined as the proportion of patients with a complete response (CR) or partial response (PR) on two consecutive occasions ≥4 weeks apart, as determined by the investigator according to Response Evaluation Criteria in Solid Tumors, Version 1.1 (RECIST v1.1)
- Duration of response (DOR), defined as the time from the first occurrence of a documented objective response to disease progression or death from any cause during the study (whichever occurs first), as determined by the investigator according to RECIST v1.1
- Progression-free survival (PFS), defined as the time from first treatment at Cycle 1 Day 1 to the first occurrence of disease progression or death from any cause during the study (whichever occurs first), as determined by the investigator according to RECIST v1.1

Biomarker Objective

The exploratory biomarker objective for this study is to identify and/or evaluate biomarkers that are potentially predictive of response to GDC-6036 as a single agent or in combination with other anti-cancer therapies (i.e., predictive biomarkers), early surrogates of activity, associated

with progression to a more severe disease state (i.e., prognostic biomarkers), associated with *intrinsic or* acquired resistance to KRAS G12C inhibitors (e.g., GDC-6036), associated with susceptibility to developing adverse events or can lead to improved adverse event monitoring or investigation (i.e., safety biomarkers), can provide evidence of GDC-6036 activity as a single agent or in combination with other anti-cancer therapies (i.e., pharmacodynamic [PD] biomarkers), or can increase the knowledge and understanding of disease biology and drug safety. Corresponding biomarker endpoints include the following:

- Relationship between exploratory biomarkers in blood, plasma, and tumor tissue and safety, PK, activity, or other biomarker endpoints

Additional Objective

An additional objective for this study is to identify a recommended Phase II dose and regimen for GDC-6036 when administered as a single agent and when administered in combination with other anti-cancer therapies on the basis of any of the following endpoints:

- Relationship between GDC-6036 exposure (PK parameters) and safety and activity endpoints
- Relationship between tumor pharmacodynamic effects of GDC-6036 and safety and activity endpoints

STUDY DESIGN

Description of Study

This is a first-in-human Phase Ia/Ib, open-label, multicenter dose-escalation and dose-expansion study designed to evaluate the safety, pharmacokinetics, and preliminary activity of GDC-6036 as a single agent and in combination with other anti-cancer therapies in patients with advanced or metastatic solid tumors that harbor the *KRAS G12C* mutation. The combination therapies in this study are [redacted] cetuximab (Arm C), [redacted] CRC, [redacted] . [redacted]

Patients who do not meet the criteria for participation in this study (screen failure) may qualify for up to two re-screening opportunities (for a total of three screenings per participant) at the investigator's discretion. Patients are not required to re-sign the consent form if they are re-screened within 30 days after previously signing the consent form. For patients who are re-screened, all eligibility criteria must be re-evaluated and screening assessments should be repeated as applicable to meet the eligibility criteria. The investigator will record reasons for screen failure in the screening log.

Patients will be enrolled in two stages: a dose-escalation stage (Stage I) and a dose-expansion stage (Stage II). Patients will be assigned to one of [redacted] as outlined in the table below. All cycles will be 21 days in length.

Stage	Arm	Study Treatment	KRAS G12C–Positive Tumor Type	Number of Patients
[redacted]	[redacted]	[redacted]	[redacted]	[redacted]
	[redacted]	[redacted]	[redacted]	
[redacted]	[redacted]	[redacted]	[redacted]	[redacted]
	[redacted]	[redacted]	[redacted]	[redacted]
	[redacted]	[redacted]	[redacted]	[redacted]
	[redacted]	[redacted]	[redacted]	[redacted]
[redacted]	[redacted]	[redacted]	[redacted]	[redacted]
I, II	Arm C	GDC-6036 + cetuximab	CRC	Stage I: n = 12<br>Stage II: n = 20
[redacted]	[redacted]	[redacted]	[redacted]	[redacted]
[redacted]	[redacted]	[redacted]	[redacted]	[redacted]
[redacted]	[redacted]	[redacted]	[redacted]	[redacted]
	[redacted]	[redacted]	[redacted]	
[redacted]	[redacted]	[redacted]	[redacted]	[redacted]
	[redacted]	[redacted]	[redacted]	[redacted]
	[redacted]	[redacted]	[redacted]	[redacted]

[redacted] CRC = colorectal cancer

[a] [redacted]

[b] [redacted]

[redacted]

During the dose-escalation stage, patients will be evaluated for DLTs at escalating dose levels to determine the maximum tolerated dose (MTD) or maximum administered dose (MAD, if the MTD was not identified) for GDC-6036 as a single agent and in combination with other anti-cancer therapies.

[redacted]

In the combination arms in Stage I, the starting dose of GDC-6036 will be no higher than one dose level below the *MTD or MAD identified* in Stage I Arm A (GDC-6036 single-agent dose escalation).

The starting dose and schedule of the combination therapy agents will be as follows: [redacted] cetuximab as an initial dose of 400 mg/m^2 IV followed by 250 mg/m^2 IV weekly in 21-day cycles; [redacted]

[redacted]

Approximately [redacted] additional patients may be enrolled in the dose-expansion stage (Stage II) at or below the MTD (or MAD, if the MTD was not identified) established during Stage I in each arm to further assess the safety, tolerability, PK, and preliminary anti-tumor activity of GDC-6036 as a single agent and in combination with other anti-cancer therapies. [redacted]

The study consists of a screening period of up to 28 days, a treatment period, and a safety follow-up period during which patients will be followed for safety outcomes *(including pregnancy testing)* for a treatment-specific period after their final dose of study drug or until they receive another anti-cancer therapy, whichever occurs first. Patients who provide a separate consent may be screened for *KRAS G12C* mutation status through central testing of circulating tumor DNA (ctDNA).

In the absence of unacceptable toxicities and unequivocal disease progression as determined by the investigator, patients may continue treatment with GDC-6036 until the end of the study.

All patients will be closely monitored for adverse events throughout the study and for a treatment-specific period after the final dose of study treatment or until initiation of another anti-cancer therapy, whichever occurs first. Adverse events will be graded according to the NCI CTCAE v5.0.

To characterize the PK properties of GDC-6036, plasma samples will be taken at various timepoints before and after dosing.

Approximately [redacted] patients are expected be enrolled in this study, at approximately [redacted] investigative sites in North America, Europe, and Asia-Pacific. [redacted]

[redacted]

Dose-Escalation in Combination Therapy Arms (Stage I, Arms [redacted] C, [redacted])

Up to approximately [redacted] patients will be enrolled across dose-escalation Stage I for the following combination therapy arms: [redacted] Arm C (GDC-6036 in combination with cetuximab)[redacted]

The starting dose of GDC-6036 in the dose-escalation stage of the combination arms will be no higher than one dose level below the *MTD or MAD identified* in Stage I Arm A (GDC-6036 single-agent dose escalation) and will follow a 3+3 dose-escalation design. The starting dose and schedule of the combination therapy agents [redacted] will be as follows: [redacted]; cetuximab as an initial dose of 400 mg/m^2 IV followed by 250 mg/m^2 IV weekly in 21-day cycles (Arm C); [redacted]

Enrollment of the first 2 patients in all 3+3 dose-escalation cohorts will be separated by at least 24 hours. Patients will be closely monitored for adverse events during a DLT assessment window, defined as Days 1–21 of Cycle 1. Adverse events identified as DLTs, as defined in the protocol, will be reported to the Sponsor within 24 hours.

Patients who discontinue from study treatment prior to completing the DLT assessment window for reasons other than a DLT will be considered non-evaluable for dose-escalation decisions and MTD or MAD assessments, and will be replaced by an additional patient at that same dose level. Patients who miss more than 25% of doses of any study drug during the DLT assessment window for reasons other than a DLT will also be replaced. Patients whose GDC-6036 dose is reduced during the DLT assessment window for reasons other than DLT may be replaced. Patients who receive supportive care during the DLT assessment window that confounds the evaluation of DLTs (not including supportive care described below as part of the DLT definition) may be replaced at the discretion of the Medical Monitor.

In addition, patients who have received KRAS G12C inhibitors prior to enrollment and for whom screening tissue and/or circulating tumor DNA assessments indicate a second KRAS mutation (in addition to KRAS G12C) following study enrollment may be replaced, and the investigator will be notified of the detection of a second KRAS mutation.

The initiation of combination arms (Arms [redacted] *C,* [redacted]*) may be subject to country-specific approval by appropriate regulatory agencies and/or Institutional Review Boards/Ethics Committees (IRBs/ECs). Initiation of combination arms (Arms* [redacted] *C,* [redacted]*) could be made contingent on the review of the data from the single-agent dose escalation (Stage I Arm A). If regulatory approval for initiating the combination arms is formally required, this will be documented appropriately.*

Dose-Expansion Stage (Stage II)

A total of approximately [redacted] patients will be enrolled in the dose-expansion stage (Stage II), with approximately 20 patients in each of the following arms, [redacted]

- [redacted]
- Stage II Arm C: GDC-6036 with cetuximab in patients with CRC
- [redacted]

Patients will be treated at or below the MTD or MAD (if the MTD was not identified) of GDC-6036 as a single agent or in combination with other anti-cancer therapies determined in the dose-escalation stage of each regimen to obtain additional safety, tolerability, and PK data, as well as preliminary evidence of clinical activity.

The Sponsor, in consultation with the investigators, will evaluate all available safety data on an ongoing basis to assess the tolerability of the dose level(s) studied. If the frequency of Grade 3 or 4 toxicities or other unacceptable toxicities at the initial expansion-stage dose level suggests that the safety or tolerability of the selected GDC-6036 dose level is unacceptable, accrual at that dose level will be halted, and patients who continue on study treatment will be allowed to reduce the GDC-6036 dose. Consideration will then be given to enrolling patients in an expansion cohort at a lower dose level. In addition, if accumulating tolerability, PK, or PD data suggest that the dose level in an expansion stage cohort is suboptimal for evaluation of anti-tumor activity, consideration will be given to enrolling new patients in that cohort at a different dose level. At no time will a dose level studied in the expansion stage exceed the highest dose level that has met escalation criteria in the dose-escalation stage.

[redacted]

Patients who do not receive GDC-6036 or discontinue GDC-6036 treatment before completing one cycle may be replaced. *Patients who have received KRAS G12C inhibitors prior to enrollment and for whom screening tissue and/or circulating tumor DNA assessments indicate a second KRAS mutation (in addition to KRAS G12C) following enrollment may be replaced, and the investigator will be notified of the detection of a second KRAS mutation.*

[redacted]

[redacted]

<u>Internal Monitoring Committee</u>

An Internal Monitoring Committee (IMC) will be formed before enrollment into any Stage II expansion arm. At a minimum, the IMC will consist of the following Sponsor representatives associated with the study (Medical Monitor, safety scientist, biostatistician, and clinical pharmacologist), as well as a designated Sponsor medical oncologist not associated with the study who will be the IMC Chair. The IMC will operate according to a pre-specified charter;

this charter will be made available upon request to the appropriate regulatory agencies. The IMC will periodically evaluate the accumulating safety data from all patients treated in this study and will make recommendations about the study conduct, as defined in the IMC charter.

Number of Patients

Approximately [redacted] patients with *KRAS G12C*–positive cancer, including but not limited to NSCLC and CRC, will be enrolled in this study.

Target Population

Inclusion Criteria

In addition to the general inclusion criteria, patients must meet the additional criteria listed below for entry into specific cohorts.

General Inclusion Criteria

Patients must meet the following criteria for study entry:

- Signed Informed Consent Form
- Age ≥ 18 years at time of signing Informed Consent Form
- Ability to comply with the study protocol, in the investigator's judgment
- Evaluable or measurable disease per RECIST v1.1
- Eastern Cooperative Oncology Group (ECOG) performance status of 0 or 1
- Life expectancy of ≥12 weeks
- Adequate hematologic and organ function within 14 days prior to initiation of study treatment, defined by the following:
  - Absolute neutrophil count ≥1200/μL
  - Hemoglobin ≥9 g/dL
  - Platelet count ≥100,000/μL
  - Total bilirubin ≤1.5 × ULN
  - Serum albumin ≥2.5 g/dL
  - AST and ALT ≤2.5 × ULN with the following exception:

    Patients with documented liver metastases may have AST and/or ALT ≤5.0 × ULN.
  - Serum creatinine ≤1.5 × ULN or creatinine clearance ≥50 mL/min on the basis of the Cockcroft-Gault estimation:

    $$\frac{(140 - \text{age}) \times (\text{weight in kg}) \times (0.85 \text{ if female})}{72 \times (\text{serum creatinine in mg/dL})}$$
  - INR < 1.5 × ULN and aPTT <1.5 × ULN

    This applies only to patients who are not receiving therapeutic anticoagulation. Patients receiving therapeutic anticoagulation should be on a stable dose for at least 1 week prior to Cycle 1 Day 1.
- For women of childbearing potential: Agreement to remain abstinent (refrain from heterosexual intercourse) or use contraception, and agreement to refrain from donating eggs, as defined below:

  Women must remain abstinent or use contraceptive methods with a failure rate of < 1% per year and must refrain from donating eggs during the treatment period and after the final dose of study treatment for at least:

  - 6 months for GDC-6036
  - [redacted]
  - *2 months for cetuximab (Arm C)*
  - [redacted]

– [redacted]

A woman is considered to be of childbearing potential if she is postmenarchal, has not reached a postmenopausal state (≥ 12 continuous months of amenorrhea with no identified cause other than menopause), and is not permanently infertile due to surgery (i.e., removal of ovaries, fallopian tubes, and/or uterus) or another cause as determined by the investigator (e.g., Müllerian agenesis). The definition of childbearing potential may be adapted for alignment with local guidelines or regulations.

Examples of contraceptive methods with a failure rate of < 1% per year include bilateral tubal ligation, male sterilization, hormonal contraceptives that inhibit ovulation, hormone-releasing intrauterine devices, and copper intrauterine devices.

The reliability of sexual abstinence should be evaluated in relation to the duration of the clinical trial and the preferred and usual lifestyle of the patient. Periodic abstinence (e.g., calendar, ovulation, symptothermal, or postovulation methods) and withdrawal are not adequate methods of contraception. If required per local guidelines or regulations, locally recognized adequate methods of contraception and information about the reliability of abstinence will be described in the local Informed Consent Form.

- For men who are not surgically sterile: Agreement to remain abstinent (refrain from heterosexual intercourse) or use contraception, and agreement to refrain from donating sperm, as defined below:

  With a female partner of childbearing potential who is not pregnant, men must remain abstinent or use a condom plus an additional contraceptive method that together result in a failure rate of < 1% per year and must refrain from donating sperm during the treatment period and after the final dose of study treatment for at least:

  – 4 months for GDC-6036

  – *2 months for* cetuximab (Arm C)

  – [redacted]

  With pregnant female partners, men must remain abstinent or use a condom to avoid exposing the embryo during the treatment period and after the final dose of study treatment for at least:

  – 4 months for GDC-6036

  – *2 months for* cetuximab (Arm C)

  – [redacted]

  The reliability of sexual abstinence should be evaluated in relation to the duration of the clinical trial and the preferred and usual lifestyle of the patient. Periodic abstinence (e.g., calendar, ovulation, symptothermal, or postovulation methods) and withdrawal are not adequate methods of contraception. If required per local guidelines or regulations, locally recognized adequate methods of contraception and information about the reliability of abstinence will be described in the local Informed Consent Form.

- Confirmation of biomarker eligibility: Valid results from either central testing of blood or local testing of blood or tumor tissue documenting the presence of the *KRAS G12C* mutation

  Local testing of blood or tumor tissue must be performed using a Sponsor-approved validated polymerase chain reaction (PCR)-based or next-generation sequencing (NGS) assay performed at a CLIA or equivalently certified laboratory.

Patients without available local test results for *KRAS G12C* mutation status must submit a blood sample to determine whether an eligible *KRAS G12C* mutation is present by the NGS-based FoundationOne® Liquid CDx (F1L CDx) Assay.

- Submission of a freshly collected pre-treatment blood sample, whether patients are enrolled by local or central test results for the *KRAS G12C* mutation
- Consent to provide fresh (preferred) or archival tumor tissue specimen

  It is preferred that the specimen be from the most recently collected and available tumor tissue (within 5 years), and whenever possible, from a metastatic site of disease. See the laboratory manual for instructions.

  For patients enrolled in Stage I, confirmation of available tumor tissue sample is required. For patients enrolled in Stage II, confirmation of shipment of tumor tissue is required.
- *For* patients with [redacted] and adenocarcinoma of the colon or rectum: *patients* must not have a known concomitant second oncogenic driver ([redacted] or *for patients with* adenocarcinoma of the colon or rectum: *BRAF V600E* mutation, *ERBB2* amplification) as determined by the Foundation Medicine, Inc. (FMI) NGS assay or by a Sponsor-approved validated PCR-based or NGS assay performed at a local CLIA-certified or equivalently-certified laboratory
- *For patients requiring accessible lesion(s) that permit a total of at least two biopsies (pre-treatment and on-treatment) without unacceptable risk of a significant procedural complication:*

  In lieu of a fresh pre-treatment biopsy, a recently obtained biopsy performed after completion of the last anti-cancer therapy will be acceptable.

  Acceptable samples include core needle biopsies for deep tumor tissue or lymph nodes or excisional, incisional, punch, or forceps biopsies for cutaneous, subcutaneous, or mucosal lesions. Fine needle aspirates, cell pellets from effusions or ascites, lavage samples, and bone biopsies are not permitted. Target lesions considered for core needle biopsies should be deemed suitable for retrieval of at least three cores at a given time point (minimum diameter 18-gauge, if feasible).

  If multiple lesions are available, it is preferable to obtain the on-treatment biopsy from the same lesion (or organ) as the pre-treatment biopsy, if feasible, to avoid introduction of heterogeneity related to site of metastasis.

[redacted]

[redacted]

[redacted]

If a patient that has progressed after at least one available standard therapy has [redacted]

[redacted]

Stage I and Stage II Arm C (Cetuximab Combination)

- Histologically documented, locally advanced, recurrent, or metastatic incurable adenocarcinoma of the colon or rectum

  Patients with appendiceal tumors are excluded.

  Patients must have experienced disease progression or intolerance to at least one prior chemotherapy regimen (e.g., FOLFOX, FOLFIRI, FOLFOXIRI ± bevacizumab)

  If a patient that has progressed after at least one available standard therapy has additional approved standard treatment options available, the study doctor must *offer these additional approved treatment options and* discuss the risks and benefits of those treatments before informed consent to participate in this study is obtained. This discussion must be documented in patient records.

- For patients who have previously been treated with a KRAS G12C inhibitor, consent to provide the following:
  - An archival tumor tissue specimen collected prior to treatment with the KRAS G12C inhibitor (e.g., diagnostic specimen)
  - A recently acquired tumor specimen after completion of the last KRAS G12C inhibitor treatment

[redacted]

[redacted]

[redacted]

Exclusion Criteria

In addition to the general exclusion criteria, patients who meet any of the additional criteria listed below will be excluded from entry into specific cohorts.

General Exclusion Criteria

Patients who meet any of the following criteria will be excluded from study entry:

- Inability or unwillingness to swallow pills
- Inability to comply with study and follow-up procedures

- Malabsorption syndrome or other condition that would interfere with enteral absorption
- Known and untreated, or active central nervous system (CNS) metastases (progressing or requiring anticonvulsants or corticosteroids for symptomatic control)

  Patients with a history of treated CNS metastases are eligible, provided they meet all of the following criteria:

  - Measurable or evaluable disease outside the CNS
  - No history of intracranial hemorrhage or spinal cord hemorrhage
  - No ongoing requirement for corticosteroids as therapy for CNS metastases, with corticosteroids discontinued for $\geq$ 2 weeks prior to enrollment and no ongoing symptoms attributed to CNS metastases
  - No stereotactic radiation within 7 days or whole-brain radiation within 14 days prior to Day 1 of Cycle 1
  - No evidence of interim progression between the completion of CNS-directed therapy and the screening radiographic study
  - Note: Patients with new asymptomatic CNS metastases detected at screening are eligible for the study after receiving radiotherapy and/or surgery. Following treatment, these patients may be eligible without the need to repeat the additional brain scan, if all other criteria are met.
- Leptomeningeal disease or carcinomatous meningitis
- Uncontrolled pleural effusion, pericardial effusion, or ascites requiring recurrent drainage procedures biweekly or more frequently

  Indwelling pleural or abdominal catheters may be allowed, provided the patient has adequately recovered from the procedure, is hemodynamically stable and symptomatically improved, and after discussion with the Medical Monitor.
- Any active infection that, in the opinion of the investigator, could impact patient safety, or serious infection requiring IV antibiotics within 7 days prior to Day 1 of Cycle 1

  In the setting of a pandemic or epidemic, screening for active infections should be considered according to local or institutional guidelines or those of applicable professional societies (e.g., American Society of Clinical Oncology or European Society for Medical Oncology).
- Clinically significant history of liver disease, including viral or other hepatitis, current alcohol abuse, or cirrhosis
- Known HIV infection

  [redacted]
- Any other diseases, active or uncontrolled pulmonary dysfunction, metabolic dysfunction, physical examination finding, or clinical laboratory finding giving reasonable suspicion of a disease or condition that contraindicates the use of an investigational drug, that may affect the interpretation of the results, or renders the patients at high risk from treatment complications
- Uncontrolled hypercalcemia ($>$ 1.5 mmol/L ionized calcium or calcium $>$ 12 mg/dL or corrected serum calcium $\geq$ ULN) or symptomatic hypercalcemia requiring continued use of bisphosphonate therapy or denosumab
- Significant traumatic injury or major surgical procedure within 4 weeks prior to Day 1 of Cycle 1
- Patients with chronic diarrhea, short bowel syndrome or significant upper gastrointestinal surgery including gastric resection, a history of inflammatory bowel disease (e.g., Crohn's disease or ulcerative colitis) or any active bowel inflammation (including diverticulitis)

- Prior treatment with any KRAS G12C inhibitor, with the following exceptions:

  Patients in Arm C [redacted] only may have had prior KRAS G12C inhibitor treatment. *These patients must not have discontinued prior KRAS G12C inhibitor treatment because of intolerance or toxicity assessed as related to the prior KRAS G12C inhibitor.*

- Treatment with chemotherapy, immunotherapy, biologic therapy *or an investigational agent* as anti-cancer therapy within 3 weeks *or five half-lives* prior to initiation of study treatment*, whichever is shorter*, or endocrine therapy within 2 weeks prior to initiation of study treatment, except for the following:

  Hormonal therapy with gonadotropin-releasing hormone (GnRH) agonists or antagonists for endocrine sensitive cancers (e.g., prostate, endometrial, hormone receptor-positive breast cancer)

  Kinase inhibitors, approved by regulatory authorities, may be used up to 2 weeks prior to initiation of study treatment, provided any drug-related toxicity has completely resolved and after discussion with the Medical Monitor.

- Radiation therapy (other than palliative radiation to bony metastases and radiation to CNS metastases as described above) as cancer therapy within 4 weeks prior to initiation of study treatment
- Palliative radiation to bony metastases within 2 weeks prior to initiation of GDC-6036
- Adverse events from prior anti-cancer therapy that have not resolved to Grade ≤ 1 except for alopecia, vitiligo, endocrinopathy managed with replacement therapy, or Grade ≤ 2 peripheral neuropathy
- History of other malignancy within 5 years prior to screening, with the exception of patients with a negligible risk of metastasis or death and/or treated with expected curative outcome (such as appropriately treated carcinoma in situ of the cervix, non-melanoma skin carcinoma, localized prostate cancer, ductal carcinoma in situ, or Stage I uterine cancer)
- History of or active clinically significant cardiovascular dysfunction, including the following:
  - History of stroke or transient ischemic attack within 6 months prior to first dose of study treatment
  - History of myocardial infarction within 6 months prior to first dose of study treatment
  - New York Heart Association Class III or IV cardiac disease or congestive heart failure requiring medication
  - Uncontrolled arrhythmias, history of or active ventricular arrhythmia requiring medication
  - Coronary heart disease that is symptomatic or unstable angina
  - Congenital long QT syndrome or QT interval corrected through use of Fridericia's formula (QTcF) $>$470 ms demonstrated by at least two ECGs $>$30 minutes apart, or family history of sudden unexplained death or long QT syndrome
  - Current treatment with medications that are well known to prolong the QT interval
- Pregnant or breastfeeding, or intending to become pregnant during the study or within 6 months after the final dose of GDC-6036

  Women of childbearing potential (including those who have had a tubal ligation) must have a negative serum pregnancy test result within 14 days prior to initiation of study drug.

- *Known hypersensitivity to any of the components of GDC-6036*

[redacted]

Stage I and Stage II Arm C (Cetuximab Combination)

- History of idiopathic pulmonary fibrosis, organizing pneumonia (e.g., bronchiolitis obliterans), drug-induced pneumonitis, or idiopathic pneumonitis, or evidence of active pneumonitis on screening chest computed tomography (CT) scan

  History of radiation pneumonitis in the radiation field (fibrosis) is permitted.

- Known hypersensitivity to any of the components of cetuximab

[redacted]

[redacted]

[redacted]

[redacted]

End of Study

The end of this study is defined as the date when the last patient, last visit (LPLV), occurs. LPLV is expected to occur 12 months after the last patient is enrolled.

Length of Study

The total length of the study, from screening of the first patient to the end of the study, is expected to be approximately 42 months.

INVESTIGATIONAL MEDICINAL PRODUCTS

Test Product (Investigational Drug)

The investigational medicinal products (IMPs) for this study are GDC-6036, [redacted] cetuximab, [redacted] The reference safety information documents for GDC-6036, [redacted] are the respective Investigator's

Brochures, and the reference safety information documents for cetuximab and [redacted] are the respective Summaries of Product Characteristics.

GDC-6036

GDC-6036 will be supplied by the Sponsor as an active pharmaceutical ingredient (API) powder-in-capsule (PIC) formulation in three strengths: 5 mg, 25 mg, and 100 mg (free base equivalent). Additionally, a film-coated tablet formulation in a dose strength of 100 mg (free base equivalent) will also be supplied for clinical use.

[redacted]

In [redacted] *Stage I combination arms (Arms* [redacted] *C,* [redacted]*),* and Stage II expansion cohorts *(Arms* [redacted] *C,* [redacted] QD dosing of GDC-6036 will begin on Day 1 of Cycle 1 and each cycle will be 21 days in length.

For GDC-6036 doses to be administered at home, a sufficient number of capsules or tablets should be dispensed to the patient to last until the next visit, or at the investigator's discretion, through one cycle. Patients will self-administer GDC-6036 as detailed below, except on study visit days when GDC-6036 will be administered in the clinic.

Patients should take GDC-6036 at approximately the same time each day unless otherwise instructed. Patients will be instructed as to the number and strength of capsules or tablets to take, according to their assigned dose level and schedule. Patients will be asked to record the time and date that they take each dose in a medication diary.

[redacted]

Cetuximab

Cetuximab will be supplied by the Sponsor in commercially available formulations, or supplied by the study sites and reimbursed by the Sponsor. Cetuximab will be administered at an initial dose of 400 mg/m^2 as a 120-minute IV infusion on Day 1 followed by 250 mg/m^2 as a 60-minute IV infusion weekly, in 21-day cycles. The maximum infusion rate must not exceed *5 mg/min for the initial administration and* 10 mg/min *for the subsequent administrations.* Cetuximab should be administered following administration of GDC-6036.

[redacted]

STATISTICAL METHODS

Primary Analysis

The safety analysis population will consist of all patients who received at least one dose of study drug, with patients grouped according to treatment received.

Safety will be assessed through summaries of exposure to study treatment, adverse events, changes in laboratory test results, and changes in vital signs and ECGs.

Study treatment exposure (such as treatment duration, total dose received, and number of cycles and dose modifications) will be summarized with descriptive statistics.

All verbatim adverse event terms will be mapped to Medical Dictionary for Regulatory Activities (MedDRA) thesaurus terms, and adverse event severity will be graded according to NCI CTCAE v5.0. All adverse events, serious adverse events, adverse events leading to death, adverse events of special interest, and adverse events leading to study treatment discontinuation that occur on or after the first dose of study treatment (i.e., treatment emergent adverse events) will be summarized by mapped term, appropriate thesaurus level, and severity grade. For events of varying severity, the highest grade will be used in the summaries. Deaths and cause of death will be summarized.

Relevant laboratory, vital sign (pulse rate, respiratory rate, blood pressure, pulse oximetry, and temperature; oxygen saturation at screening), and ECG data will be displayed by time, with grades identified where appropriate. Additionally, a shift table of selected laboratory tests will be used to summarize the baseline and maximum postbaseline severity grade. Changes in vital signs and ECGs will be summarized.

Determination of Sample Size

This study is intended to obtain preliminary safety, PK, and activity information in the safety-evaluable population. The sample sizes do not reflect any explicit power and type I error considerations.

Dose Escalation (Stage I)

The sample size for this trial is based on the dose-escalation rules described in the protocol. The planned enrollment is approximately [redacted] and [redacted] patients for the combination dose-escalation stage (Arms [redacted] C, [redacted]). [redacted] Stage I dose-escalation arms in combination with other anti-cancer therapies will follow a 3+3 design based on the criteria described in the protocol. The protocol provides probabilities of observing no DLT in 3 patients or observing ≤ 1 DLT in 6 patients, given different underlying DLT rates.

Dose Expansion (Stage II)

The planned enrollment for the expansion stage of this study (Stage II [redacted] Arm C, [redacted]) is up to approximately [redacted] patients.

Approximately 20 patients each will be enrolled in [redacted] CRC, and [redacted] cohorts to assess the safety, tolerability, and preliminary evidence of anti-tumor activity of GDC-6036 administered at or below its MTD or MAD as a single agent or in combination with other anti-cancer therapies. The protocol provides probabilities of observing at least one adverse event among 20 patients for probabilities ranging from 0.01 to 0.2 (i.e., adverse event frequencies of 1%–20%) *and illustrates that the planned sample size is sufficient to provide a meaningful likelihood of observing adverse events occurring at appreciable frequency (5% or higher). For a given adverse event with a true rate of 20%, 10%, or 5%, the probability of observing at least one adverse event in an expanded cohort of 20 patients is 0.99, 0.88, and 0.64, respectively. All available safety data will be evaluated to assess the tolerability of the treatment for a specific indication. In addition, the cohort size of 20 patients will enable a preliminary assessment of anti-tumor activity. The observed ORR will be used to evaluate the potential benefit for patients.*

LIST OF ABBREVIATIONS AND DEFINITIONS OF TERMS

Abbreviation	Definition
AUC	area under the plasma concentration–time curve
BCRP	*breast cancer resistance protein*
BID	twice a day
CDx	companion diagnostic
CLIA	Clinical Laboratory Improvement Amendments
C_{max}	maximum plasma concentration observed
C_{min}	minimum plasma concentration observed
CMV	cytomegalovirus
CR	complete response
CRC	colorectal cancer
CSF	colony-stimulating factor
CT	computed tomography (scan)
ctDNA	circulating tumor DNA
CTCAE	Common Terminology Criteria for Adverse Events
DLT	dose-limiting toxicity
DOR	duration of response
EC	Ethics Committee
ECOG	Eastern Cooperative Oncology Group
eCRF	electronic Case Report Form
EDC	electronic data capture
EGFR	epidermal growth factor receptor
E.U.	*European Union*
F1CDx	FoundationOne®CDx
F1L CDx	FoundationOne®Liquid CDx
FDA	(U.S.) Food and Drug Administration
FDG-PET	^{18}F-fluorodeoxyglucose positron emission tomography
FFPE	formalin-fixed, paraffin-embedded
FMI	Foundation Medicine, Inc.
G12	glycine 12
G12C	change from glycine 12 to cysteine
GI	gastrointestinal
GnRH	gonadotropin-releasing hormone
[redacted]	[redacted]
[redacted]	[redacted]

Abbreviation	Definition
[redacted]	[redacted]
[redacted]	[redacted]
[redacted]	[redacted]
HED	human equivalent dose
HIPAA	Health Insurance Portability and Accountability Act
IC_{90}	concentration associated with 90% target inhibition
ICH	International Council for Harmonisation
IHC	immunohistochemistry
IMC	*Internal Monitoring Committee*
IMP	investigational medicinal product
IND	Investigational New Drug (Application)
IRB	Institutional Review Board
[redacted]	[redacted]
IxRS	interactive voice or web-based response system
KRAS	Kirsten rat sarcoma viral oncogene homolog
LPLV	last patient, last visit
MAD	maximum administered dose
MRI	magnetic resonance imaging
[redacted]	[redacted]
[redacted]	[redacted]
mRNA	messenger RNA
MTD	maximum tolerated dose
NCCN	National Comprehensive Cancer Network
NCI	National Cancer Institute
NCI CTCAE v5.0	National Cancer Institute Common Terminology Criteria for Adverse Events, Version 5.0
NGS	next-generation sequencing
NOAEL	no-observed-adverse-effect level
[redacted]	[redacted]
ORR	objective response rate
PBPK	*physiologically based pharmacokinetic*
PCR	polymerase chain reaction
PD	pharmacodynamic
PD-1	programmed cell death protein 1
PD-L1	programmed death-ligand 1
PFS	progression-free survival

Abbreviation	Definition
P-gp	*P-glycoprotein*
PIC	powder in capsule (formulation)
PK	pharmacokinetic
PO	by mouth; orally
PR	partial response
QD	once a day
QTcF	QT interval corrected through use of Fridericia's formula
RBR	Research Biosample Repository
RECIST v1.1	Response Evaluation Criteria in Solid Tumors, Version 1.1
SD	stable disease
[redacted]	[redacted]
SmPC	Summary of Product Characteristics
STD_{10}	severely toxic dose to 10%
$t_{1/2}$	half-life
t_{max}	time to maximum plasma concentration
ULN	upper limit of normal
[redacted]	[redacted]
WES	whole exome sequencing
WGS	whole genome sequencing

1. BACKGROUND

1.1 BACKGROUND ON *KRAS G12C* AND CANCER

The Kirsten rat sarcoma viral oncogene homolog (*KRAS*) is a central component of the RAS/MAPK signal transduction pathway, an intracellular network of proteins that transmit extracellular growth factor signals to regulate cell proliferation, differentiation, and survival. Mutations in *KRAS* can result in alterations at several amino acids, including glycine 12 (G12), glycine 13, and glutamine 61, commonly found in solid tumors and associated with tumorigenesis and aggressive tumor growth (Der et al. 1982; Parada et al. 1982; Santos et al. 1982; Taparowsky et al. 1982; Capon et al. 1983). Oncogenic *KRAS* mutations that result in the change from G12 to cysteine (G12C) are prevalent in [redacted] colorectal cancer (CRC) (~4%), and other tumor types (≤4%) (Bailey et al. 2016; Campbell et al. 2016; Giannakis et al. 2016; Hartmaier et al. 2017; Jordan et al. 2017).

Advanced stage tumors harboring the *KRAS G12C* mutation (hereafter referred to as *KRAS G12C*–positive tumors), including [redacted] CRC, and other solid tumors, are incurable and carry a poor prognosis (Roman et al. 2018; Wan et al. 2019). In addition, patients with advanced stage *KRAS G12C*–positive cancers may derive limited benefit from select chemotherapies and targeted therapies, thus, restricting effective available treatment options (Roman et al. 2018).

[redacted]

For advanced or metastatic CRC, systemic treatment can consist of combinations of active agents or select individual agents and includes 5-fluorouracil/leucovorin, capecitabine, irinotecan, oxaliplatin, bevacizumab, cetuximab, panitumumab, ziv-aflibercept, ramucirumab, regorafenib, trifluridine-tipiracil, pembrolizumab, nivolumab, ipilimumab, and encorafenib. Treatment is selected based on the goals of care, type and timing of prior therapy, mutational profile of the tumor, anticipated toxicity profile, and patient's performance status (NCCN 2020b). Specifically, *KRAS* mutations were associated with resistance to anti-EGFR therapies (Lièvre et al. 2006; Karapetis et al. 2008; Van Cutsem et al. 2009; Misale et al. 2012). Thus, patients whose cancers harbor *KRAS* mutations are not eligible for treatment with cetuximab or panitumumab (NCCN 2020b) and treatment options remain limited.

[redacted].

Although previously considered an "undruggable" cancer target, the landmark discovery of the switch II pocket within KRAS has enabled the development of covalent inhibitors specific for KRAS G12C (Ostrem et al. 2013). The clinical development of covalent inhibitors of KRAS G12C offers a potential therapeutic intervention to prevent or delay the progression of cancers that harbor a *KRAS G12C* mutation (McCormick 2019). Early clinical data from covalent inhibitors specific for KRAS G12C demonstrate single-agent anti-tumor activity and potential to improve treatment options and outcomes for patients with advanced stage cancer that harbors the *KRAS G12C* mutation (Jänne et al. 2019; Hong et al. 2020).

1.2 BACKGROUND ON STUDY TREATMENTS

1.2.1 GDC-6036

GDC-6036 is an oral, covalent, anti-cancer therapeutic agent that selectively inhibits KRAS G12C, but not other mutations in KRAS, the wild-type form of KRAS, or other members of the RAS family. Nonclinical studies demonstrate that treatment of *KRAS G12C*–positive cancer cell lines or tumor xenograft models with GDC-6036 results in decreased KRAS pathway signaling, suppression of proliferation, and induction of apoptosis.

In vitro and in vivo pharmacology studies demonstrate that GDC-6036 is a highly potent and selective covalent inhibitor of KRAS G12C, exhibiting over 20,000-fold selectivity in growth inhibition for *KRAS G12C*–positive over *KRAS G12C*–negative cancer cell lines. Mechanism of action studies with GDC-6036 demonstrate that downstream MAPK pathway components such as phosphorylated (p)ERK and pS6, in addition to *KRAS* target genes such as *DUSP6* and *SPRY4*, are inhibited and apoptosis induction is observed in *KRAS G12C*–positive cancer cell lines. In addition, GDC-6036 has potent single-agent activity and inhibits tumor growth in a number of nonclinical xenograft models of *KRAS G12C*–positive lung tumors. These in vitro and in vivo pharmacology studies support the use of GDC-6036 for the treatment of patients with locally advanced or metastatic *KRAS G12C*–positive solid tumors.

The results of nonclinical toxicology studies completed to date provide a robust characterization of the toxicity profile of GDC-6036 and support the administration of GDC-6036 in a first-in-human Phase I trial in patients with cancer. Comprehensive nonclinical toxicity studies were completed to evaluate the potential single and repeat dose oral toxicity, genetic toxicity, phototoxicity, and safety pharmacology of GDC-6036.

Because the *KRAS G12C* mutation is not present in healthy animals, there are no pharmacologically relevant nonclinical species for KRAS G12C inhibition. However, nonclinical studies were performed to assess the risk related to wild-type *KRAS* inhibition and off-target effects of GDC-6036. [redacted]

[redacted]

[redacted]

Results from the nonclinical toxicity and safety pharmacology studies completed to date characterize the toxicology profile of GDC-6036 and support the administration of

GDC-6036 to patients with advanced cancer. Toxicities observed in nonclinical species are expected to be manageable and/or monitorable in a clinical setting.

Refer to the GDC-6036 Investigator's Brochure for details on nonclinical studies.

1.2.2 <u>Combination Treatments</u>

[redacted]

[redacted]

[redacted]

[redacted]

1.2.2.2 Cetuximab

Cetuximab is a chimeric monoclonal IgG1 antibody that is specifically directed against the epidermal growth factor receptor (EGFR, also known as HER1 or c-ErbB-1).

The EGFR is a transmembrane glycoprotein that is a member of a subfamily of type I receptor tyrosine kinases including EGFR, HER2, HER3, and HER4. The EGFR is constitutively expressed in many normal epithelial tissues, including the skin and hair follicle. Expression of EGFR is also detected in many human cancers, including those of the head and neck, colon, and rectum.

Cetuximab binds specifically to the EGFR on both normal and tumor cells and competitively inhibits the binding of epidermal growth factor (EGF) and other ligands, such as transforming growth factor-alpha.

Cetuximab is approved for the treatment of squamous cell carcinoma of the head and neck and *KRAS* wild-type, EGFR-expressing metastatic colorectal cancer.

Refer to the Cetuximab E.U. Summary of Product Characteristics (SmPC) for details on nonclinical and clinical studies.

[redacted]

[redacted]

[redacted]

[redacted]

[redacted]

[redacted]

[redacted]

[redacted]

[redacted]

1.3 STUDY RATIONALE AND BENEFIT–RISK ASSESSMENT

[redacted]

[redacted]

[redacted]

[redacted]

[redacted]

[redacted]

1.3.2 GDC-6036 in Combination Therapy

Early Phase I clinical data from the ongoing studies of AMG 510 and MRTX849 as single agents have shown that KRAS G12C inhibitors are tolerable and have promising anti-tumor activity in patients with metastatic [redacted] and CRC (Janne et al. 2019; Hong et at. 2020). However, there still remains a great unmet need to improve upon the anti-tumor activity and durability reported in [redacted] and CRC with this class of inhibitors as a single agent while importantly retaining their tolerable safety profile.

One strategy to improve upon the efficacy of KRAS G12C inhibitors focuses on inhibiting additional nodes upstream or downstream of KRAS in the RTK-RAS-MAPK pathway, or [redacted] This approach aims to more comprehensively shut down primary MAPK signaling and/or compensatory signaling that can drive tumor growth.

[redacted]

Together, the mechanistic rationale along with data from in vitro and in vivo combination studies (refer to the GDC-6036 Investigator's Brochure) have guided the selection of anti-cancer therapies to be evaluated in combination with GDC-6036. Combination arms of this study will evaluate the safety, tolerability, PK and PD effects, [redacted] (when applicable), and preliminary anti-tumor activity of GDC-6036 in combination with other anti-cancer therapies. *Additional details* on the rationale *and benefit–risk assessment* for each combination are provided in *Section 3.4.3*.

1.3.3 Treatment in the Setting of the COVID-19 Pandemic

In the setting of the COVID-19 pandemic, patients with comorbidities, including those with cancer, are a more vulnerable population. Infection with SARS-CoV-2 has been associated with higher morbidity and mortality in patients with cancer in some retrospective analysis. It is unclear whether or how cancer therapies such as chemotherapy, targeted therapy, or immunotherapy impact the incidence or severity of COVID-19 *infection*. It is not anticipated that GDC-6036 will increase the risk of infection with SARS-CoV-2. *There is currently a lack of vaccine efficacy and immunogenicity data, specifically in the cancer population. Based on the published mechanism of action of inactivated SARS-CoV-2 vaccines and the known mechanism*

of action of GDC-6036 and the combination therapies including cetuximab, [redacted] *there is no scientific rationale to expect that the SARS-CoV-2 vaccines will affect the efficacy or safety of these therapies and vice versa. The inactivated SARS-CoV-2 vaccines should be given in accordance with the approved/authorized vaccine label and local official immunization guidance, and each administration of a SARS-CoV-2 vaccine should be recorded as concomitant medication.*

Severe COVID-19 is associated with a cytokine release syndrome (CRS) involving the inflammatory cytokines IL-6, IL-10, IL-2 and interferon-gamma (IFN-γ) (Merad and Martin 2020). [redacted]

[redacted] If a diagnosis of COVID-19 is confirmed, the disease should be managed as per local or institutional guidelines.

2. OBJECTIVES AND ENDPOINTS

This Phase Ia/Ib study will evaluate the safety, pharmacokinetics, [redacted] preliminary activity, and biomarkers of GDC-6036 as a single agent (Arm A) and in combination with other anti-cancer therapies in patients with advanced or metastatic solid tumors with a *KRAS G12C* mutation. Combination therapies will include [redacted] cetuximab (Arm C), [redacted] in [redacted] CRC, and [redacted] Specific objectives and corresponding endpoints for the study are outlined below.

2.1 SAFETY OBJECTIVE (PRIMARY STUDY OBJECTIVE)

The safety objective for this study is to evaluate the safety of GDC-6036 as a single agent and in combination with other anti-cancer therapies on the basis of the following endpoints:

- Incidence and severity of adverse events, with severity determined according to National Cancer Institute Common Terminology Criteria for Adverse Events, Version 5.0 (NCI CTCAE v5.0)
- Incidence and nature of DLTs
- Change from baseline in targeted vital signs
- Change from baseline in targeted clinical laboratory test results
- Change from baseline in targeted ECG parameters

2.2 PHARMACOKINETIC OBJECTIVES

The pharmacokinetic (PK) objectives for this study are to characterize the PK profile of GDC-6036 when administered as a single agent and in combination with other anti-cancer therapies and to characterize the PK profile of these anti-cancer therapies when administered in combination with GDC-6036, on the basis of the following endpoints:

- Plasma concentrations of GDC-6036, [redacted] at specified timepoints
- Serum concentrations of [redacted] cetuximab, and [redacted] at specified timepoints

The exploratory PK objectives for this study are as follows:

- To evaluate potential relationships between drug exposure and the safety and activity of GDC-6036 *as a single agent and in combination with other anti-cancer therapies*
- To evaluate the exposure of potential circulating metabolites of GDC-6036 following a single or repeat oral dose(s) of GDC-6036 *as a single agent or in combination with other anti-cancer therapies*
- [redacted]

[redacted]

- [redacted]

[redacted]

- [redacted]

[redacted] [redacted]

[redacted]

[redacted]

[redacted]

2.4 ACTIVITY OBJECTIVES

The activity objective for this study is to make a preliminary assessment of the activity of GDC-6036 as a single agent and in combination with other anti-cancer therapies on the basis of the following endpoints:

- Objective response rate (ORR), defined as the proportion of patients with a complete response (CR) or PR on two consecutive occasions ≥4 weeks apart, as determined by the investigator according to Response Evaluation Criteria in Solid Tumors, Version 1.1 (RECIST v1.1)
- Duration of response (DOR), defined as the time from the first occurrence of a documented objective response to disease progression or death from any cause during the study (whichever occurs first), as determined by the investigator according to RECIST v1.1
- Progression-free survival (PFS), defined as the time from first treatment at Cycle 1 Day 1 to the first occurrence of disease progression or death from any cause during the study (whichever occurs first), as determined by the investigator according to RECIST v1.1

2.5 BIOMARKER OBJECTIVE

The exploratory biomarker objective for this study is to identify and/or evaluate biomarkers that are potentially predictive of response to GDC-6036 as a single agent or in combination with other anti-cancer therapies (i.e., predictive biomarkers), early surrogates of activity, associated with progression to a more severe disease state (i.e., prognostic biomarkers), associated with *intrinsic or* acquired resistance to KRAS G12C inhibitors (e.g., GDC-6036), associated with susceptibility to developing adverse events or can lead to improved adverse event monitoring or investigation (i.e., safety biomarkers), can provide evidence of GDC-6036 activity as a single agent or in combination with other anti-cancer therapies (i.e., pharmacodynamic [PD] biomarkers), or can increase the knowledge and understanding of disease biology and drug safety. Corresponding biomarker endpoints include the following:

- Relationship between exploratory biomarkers in blood, plasma, and tumor tissue (listed in Section 4.5.6) and safety, PK, activity, or other biomarker endpoints

2.6 ADDITIONAL OBJECTIVE

An additional objective for this study is to identify a recommended Phase II dose and regimen for GDC-6036 when administered as a single agent and when administered in combination with other anti-cancer therapies on the basis of any of the following endpoints:

- Relationship between GDC-6036 exposure (PK parameters) and safety and activity endpoints
- Relationship between tumor pharmacodynamic effects of GDC-6036 and safety and activity endpoints

3. STUDY DESIGN

3.1 DESCRIPTION OF THE STUDY

This is a first-in-human Phase Ia/Ib, open-label, multicenter dose-escalation and dose-expansion study designed to evaluate the safety, pharmacokinetics, and preliminary activity of GDC-6036 as a single agent and in combination with other anti-cancer therapies in patients with advanced or metastatic solid tumors that harbor the *KRAS G12C* mutation. The combination therapies in this study are [redacted] cetuximab (Arm C), [redacted] CRC, [redacted]. [redacted]

Patients who do not meet the criteria for participation in this study (screen failure) may qualify for up to two re-screening opportunities (for a total of three screenings per participant) at the investigator's discretion. Patients are not required to re-sign the consent form if they are re-screened within 30 days after previously signing the consent form. For patients who are re-screened, all eligibility criteria must be re-evaluated and screening assessments should be repeated as applicable to meet the eligibility criteria outlined in Section 4.1. The investigator will record reasons for screen failure in the screening log (see Section 4.5.1).

Patients will be enrolled in two stages: a dose-escalation stage (Stage I; see Section 3.1.1) and a dose-expansion stage (Stage II; see Section 3.1.2). Patients will be assigned to one of *six* regimens as outlined in Table 1. All cycles will be 21 days in length.

Table 1 Study Treatment and Tumor Types by Stage and Arm

Stage	Arm	Study Treatment	KRAS G12C–Positive Tumor Type	Number of Patients
[redacted]	[redacted]	[redacted]	[redacted]	[redacted]
	[redacted]	[redacted]	[redacted]	
[redacted]	[redacted]	[redacted]	[redacted]	[redacted]
	[redacted]	[redacted]	[redacted]	[redacted]
	[redacted]	[redacted]	[redacted]	[redacted]
	[redacted]	[redacted]	[redacted]	[redacted]
[redacted]	[redacted]	[redacted]	[redacted]	[redacted]
I, II	Arm C	GDC-6036 + cetuximab	CRC	Stage I: n = 12<br>Stage II: n = 20
[redacted]	[redacted]	[redacted]	[redacted]	[redacted]
[redacted]	[redacted]	[redacted]	[redacted]	[redacted]
[redacted]	[redacted]	[redacted]	[redacted]	[redacted]
	[redacted]	[redacted]	[redacted]	
[redacted]	[redacted]	[redacted]	[redacted]	[redacted]
	[redacted]	[redacted]	[redacted]	[redacted]
	[redacted]	[redacted]	[redacted]	[redacted]

[redacted] CRC = colorectal cancer

[a] [redacted]

During the dose-escalation stage, patients will be evaluated for DLTs at escalating dose levels to determine the maximum tolerated dose (MTD) or maximum administered dose (MAD, if the MTD was not identified) for GDC-6036 as a single agent and in combination with other anti-cancer therapies (see Section 3.1.1).

[redacted]

In the combination arms in Stage I, the starting dose of GDC-6036 will be no higher than one dose level below the *MTD or MAD identified* in Stage I Arm A (GDC-6036 single-agent dose escalation).

The starting dose and schedule of the combination therapy agents will be as follows: [redacted] cetuximab as an initial dose of 400 mg/m^2 IV followed by 250 mg/m^2 IV weekly in 21-day cycles; [redacted] (see *Section 3.1.1.2*). [redacted]

[redacted]

[redacted]

Approximately [redacted] additional patients may be enrolled in the dose-expansion stage (Stage II) at or below the MTD (or MAD, if the MTD was not identified) established during Stage I in each arm to further assess the safety, tolerability, PK, and preliminary anti-tumor activity of GDC-6036 as a single agent and in combination with other anti-cancer therapies (see Section 3.1.2). [redacted]

[redacted]

The study consists of a screening period of up to 28 days, a treatment period, and a safety follow-up period during which patients will be followed for safety outcomes *(including pregnancy testing)* for a treatment-specific period after their final dose of study drug or until they receive another anti-cancer therapy, whichever occurs first (see Section 5.3.1). Patients who provide a separate consent may be screened for *KRAS G12C* mutation status through central testing of circulating tumor DNA (ctDNA).

In the absence of unacceptable toxicities and unequivocal disease progression as determined by the investigator, patients may continue treatment with GDC-6036 until the end of the study.

All patients will be closely monitored for adverse events throughout the study and for a treatment-specific period after the final dose of study treatment or until initiation of another anti-cancer therapy, whichever occurs first (see Section 5.3.1). Adverse events will be graded according to the NCI CTCAE v5.0.

To characterize the PK properties of GDC-6036, plasma samples will be taken at various timepoints before and after dosing (see Appendix 3).

Approximately [redacted] patients are expected be enrolled in this study, at approximately [redacted] investigative sites in North America, Europe, and Asia-Pacific.

The study design is shown in Figure 1, Figure 2, Figure 3, and Figure 4. Schedules of activities are provided in Appendix 1 through Appendix 3.

[redacted]

[redacted]

Figure 2 Study Schema for Stage I Combination Arms [redacted] C, [redacted]: Dose Escalation

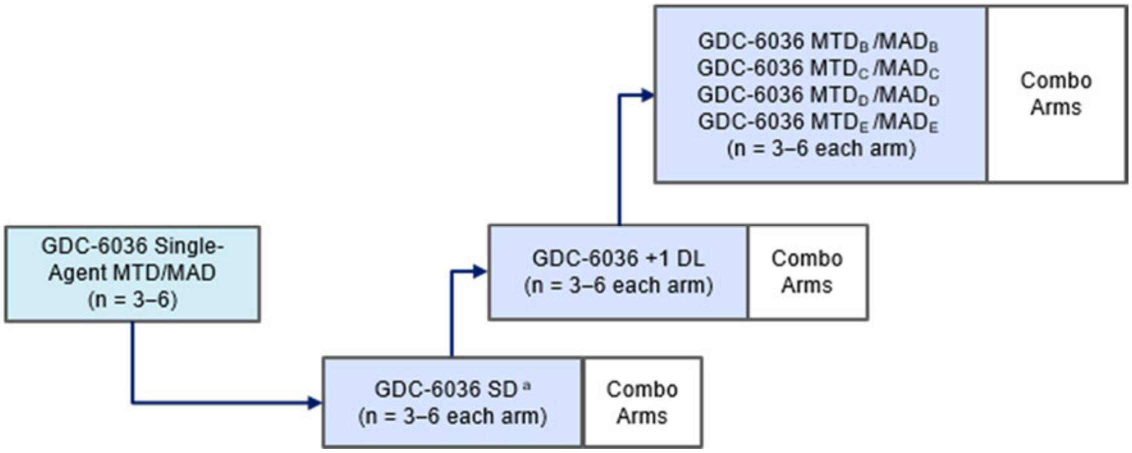

Combo = combination treatment; DL = dose level; *MAD = maximum administered dose; MTD = maximum tolerated dose;* $MTD_{(X)}$/$MAD_{(X)}$ = maximum administered dose/maximum tolerated dose for Arm (X); SD = starting dose.

[a] The starting dose of GDC-6036 in combination arms will be no higher than one dose level below the *MTD or MAD identified* in Stage I Arm A (GDC-6036 single-agent dose escalation).

[redacted]

Figure 4 Study Schema for Stage II: Dose Expansion

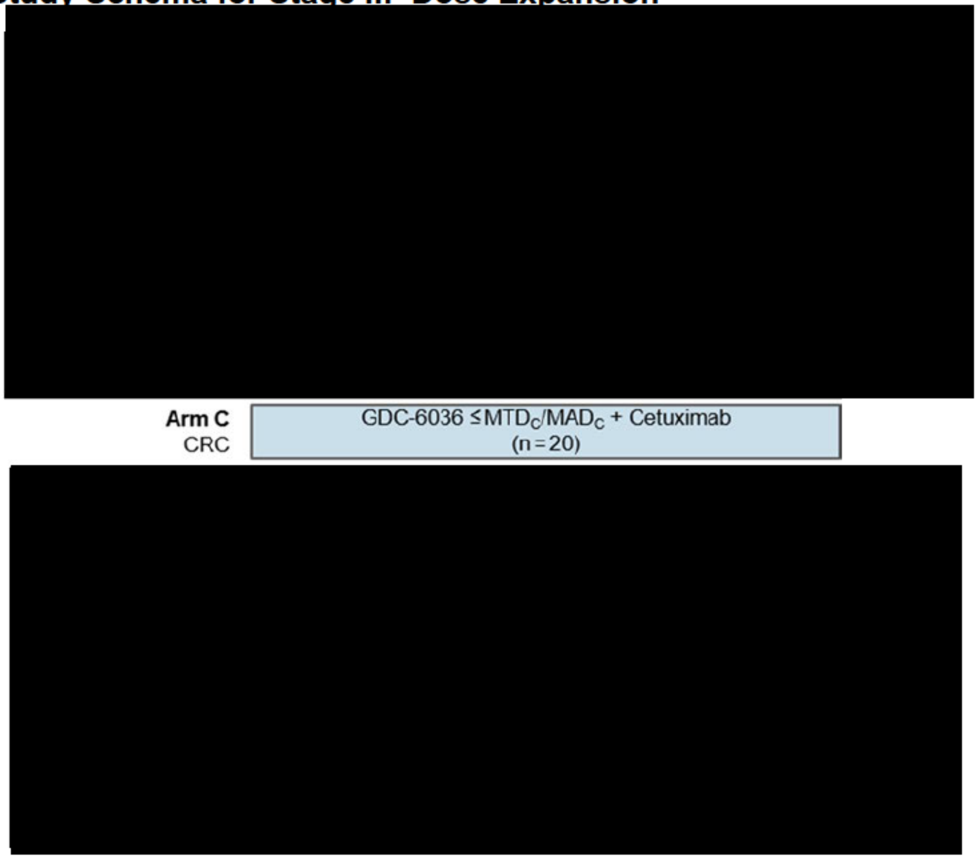

CRC = colorectal cancer; MAD = maximum administered dose; MTD = maximum tolerated dose; MTD$_{(X)}$/MAD$_{(X)}$ = maximum administered dose/maximum tolerated dose for Arm (X); [redacted]

3.1.1 Dose-Escalation Stage (Stage I)

Approximately [redacted] patients will be enrolled in the dose-escalation stage of the study.

[redacted]

[redacted]

[redacted]

[redacted]

[redacted]

[redacted]

3.1.1.2 Dose-Escalation in Combination Therapy Arms (Stage I, Arms [redacted] C, [redacted])

Up to approximately [redacted] patients will be enrolled across dose-escalation Stage I for the following combination therapy arms: [redacted] Arm C (GDC-6036 in combination with cetuximab), [redacted]

The starting dose of GDC-6036 in the dose-escalation stage of the combination arms will be no higher than one dose level below the *MTD or MAD identified* in Stage I Arm A (GDC-6036 single-agent dose escalation) and will follow a 3+3 dose-escalation design. The starting dose and schedule of the combination therapy agents [redacted] will be as follows: [redacted] cetuximab as an initial dose of 400 mg/m^2 IV followed by 250 mg/m^2 IV weekly in 21-day cycles (Arm C); [redacted]

[redacted]

Enrollment of the first 2 patients in all 3+3 dose-escalation cohorts will be separated by at least 24 hours. Patients will be closely monitored for adverse events during a DLT assessment window, defined as Days 1–21 of Cycle 1. Adverse events identified as DLTs, as defined below (see Section 3.1.1.3), will be reported to the Sponsor within 24 hours (see Section 5.4.2).

Patients who discontinue from study treatment prior to completing the DLT assessment window for reasons other than a DLT will be considered non-evaluable for dose-escalation decisions and MTD or MAD assessments, and will be replaced by an additional patient at that same dose level. Patients who miss more than 25% of doses of any study drug during the DLT assessment window for reasons other than a DLT will also be replaced. Patients whose GDC-6036 dose is reduced during the DLT assessment window for reasons other than DLT may be replaced. Patients who receive supportive care during the DLT assessment window that confounds the evaluation of DLTs (not including supportive care described below as part of the DLT definition) may be replaced at the discretion of the Medical Monitor.

In addition, patients who have received KRAS G12C inhibitors prior to enrollment and for whom screening tissue and/or circulating tumor DNA assessments indicate a second KRAS mutation (in addition to KRAS G12C) following study enrollment may be replaced, and the investigator will be notified of the detection of a second KRAS mutation.

The initiation of combination arms (Arms [redacted] C, [redacted]) may be subject to country-specific approval by appropriate regulatory agencies and/or Institutional Review Boards/Ethics Committees (IRBs/ECs). Initiation of combination arms (Arms [redacted] C, [redacted]) could be made contingent on the review of the data from the single-agent dose escalation (Stage I Arm A). If regulatory approval for initiating the combination arms is formally required, this will be documented appropriately.

3.1.1.3 Definition of Dose-Limiting Toxicity

A DLT will be defined as any of the following adverse events unless such events are attributed by the investigator to another clearly identifiable cause (e.g., documented disease progression, concomitant or preexisting medication, or intercurrent illness), occurring during the DLT assessment period:

- Grade ≥4 neutropenia (ANC <500 cells/μL) lasting >7 days
- Grade ≥3 febrile neutropenia
- Grade ≥4 anemia
- Grade ≥4 thrombocytopenia, or Grade 3 thrombocytopenia associated with clinically significant bleeding

- Grade ≥3 elevation of serum hepatic transaminase (ALT or AST) lasting >7 days

  For patients with Grade ≤2 hepatic transaminase levels at baseline as a result of liver metastases, only a transaminase level ≥10×the upper limit of normal (ULN) lasting for >7 days will be considered a DLT.

- Grade ≥3 elevation of serum bilirubin
- AST or ALT >3×ULN (or >3 × baseline value for patients with liver metastases), with concurrent increase in total bilirubin >2×ULN without evidence of cholestasis or alternative explanations (e.g., viral hepatitis, disease progression in the liver, etc.), as per Hy's Law
- [redacted]
- Grade ≥3 non-hematologic, non-hepatic adverse event, with the following exceptions:

  Grade 3 nausea, vomiting, or diarrhea that responds to standard-of-care therapy in ≤3 days will not be considered a DLT.

  Grade 3 fatigue lasting ≤3 days will not be considered a DLT.

  Grade 3 laboratory abnormalities that are asymptomatic and considered by the investigator not to be clinically significant will not be considered a DLT.

  Grade 3 hypertension that responds to standard-of-care therapy in ≤7 days will not be considered a DLT.

  Grade 3 skin and/or epithelial toxicities attributed to EGFR inhibition that improve to Grade ≤2 within 7 days with supportive care will not be considered DLTs.

[redacted]

[redacted]

[redacted]

[redacted]

[redacted]

[redacted]

[redacted]

- [redacted]
- [redacted]
- [redacted]
- [redacted]
- [redacted]

- [redacted]

[redacted]

- [redacted]

3.1.1.5 Dose-Escalation Rules: Stage I, [redacted] Arm C, [redacted]

The starting dose of GDC-6036 in the dose-escalation stage of the combination arms will be no higher than one dose level below the *MTD or MAD identified* in Stage I Arm A (GDC-6036 single-agent dose escalation). The GDC-6036 dose increment in the combination arms will not exceed that in Stage I Arm A, and the GDC-6036 dose evaluated in any of the combination arms will not exceed the single-agent MTD or MAD. [redacted]

If the MTD is exceeded at the initial dose level tested in any combination arms in Stage I, then additional patients may be enrolled to evaluate GDC-6036 and/or the combination drug at a lower dose *level*. The decision to de-escalate the doses of GDC-6036 and/or the combination drug, and the specific *dose levels* to evaluate, will be made by the Sponsor together with the investigators after review of the available PK and safety data. *For the combination agents in Arms* [redacted] *the alternate lower dose levels investigated*

will be based on the E.U. SmPC of each combination agent. [redacted]

Relevant demographic, adverse event, laboratory, dose administration, and PK (if available) data will be reviewed prior to dose-escalation decisions, which will be made by the Medical Monitor in consultation with the Principal Investigators and a committee composed of the following Sponsor representatives: statistician, safety scientist, PK scientist, and clinical trial leader.

Dose escalation in 3+3 dose-escalation cohorts will occur in accordance with the rules listed below:

- A minimum of 3 DLT-evaluable patients will be enrolled in each cohort.
- If none of the first 3 DLT-evaluable patients experiences a DLT, enrollment of the next cohort at the next highest dose level may proceed.
- If 1 of the first 3 DLT-evaluable patients experiences a DLT, the cohort will be expanded to a minimum of 6 patients. If there are no further DLTs in the first 6 DLT-evaluable patients, enrollment of the next cohort at the next dose level may proceed.
- If 2 or more DLT-evaluable patients in a cohort experience a DLT, the MTD will have been exceeded and dose escalation will stop. Up to 6 patients will be evaluated for DLTs at the preceding dose level, unless 6 patients have already been evaluated at that level. However, if the dose level at which the MTD is exceeded is ≥25% higher than the preceding dose level, an intermediate dose level may be evaluated.
- If the MTD is exceeded at any dose level, the highest dose at which fewer than 2 of 6 DLT-evaluable patients (i.e., <33%) experience a DLT will be declared the MTD.
- If the MTD is not exceeded at any dose level, the highest dose administered in this study will be declared the MAD.

On the basis of a review of real-time safety data and available preliminary PK data, dose escalation may be halted or modified by the Sponsor as deemed appropriate.

3.1.1.6 Intra-Patient Dose Escalation

For patients in Stage I *Arms A*[redacted] who have stable disease (SD) or better for two cycles and have experienced no adverse event that would have qualified as a DLT if it had occurred during the DLT assessment window, the investigator may escalate *the GDC-6036* dose to a level that previously met the criteria for dose escalation in this study on Day 1 of the next cycle, provided that the Medical Monitor has approved the dose escalation. The GDC-6036 dose may be escalated more than once for individual patients but never to a dose level that has not met the criteria for protocol-specified dose escalation.

[redacted]

Intra-patient dose-escalation decisions will be made based on the safety data available for the patient being considered for dose escalation as well as all safety data available at the time of the decision.

For analysis purposes, patients will be included in the dose-level cohorts assigned at Cycle 1 Day 1, regardless of intra-patient dose escalation.

[redacted]

[redacted]

[redacted]

3.1.2 Dose-Expansion Stage (Stage II)

3.1.2.1 General Description

A total of approximately [redacted] patients will be enrolled in the dose-expansion stage (Stage II), with approximately 20 patients in each of the following arms, [redacted]

- [redacted]
- Stage II Arm C: GDC-6036 with cetuximab in patients with CRC
- [redacted]

Patients will be treated at or below the MTD or MAD (if the MTD was not identified) of GDC-6036 as a single agent or in combination with other anti-cancer therapies determined in the dose-escalation stage of each regimen to obtain additional safety, tolerability, and PK data, as well as preliminary evidence of clinical activity.

The Sponsor, in consultation with the investigators, will evaluate all available safety data on an ongoing basis to assess the tolerability of the dose level(s) studied. If the frequency of Grade 3 or 4 toxicities or other unacceptable toxicities at the initial expansion-stage dose level suggests that the safety or tolerability of the selected GDC-6036 dose level is unacceptable, accrual at that dose level will be halted, and patients who continue on study treatment will be allowed to reduce the GDC-6036 dose. Consideration will then be given to enrolling patients in an expansion cohort at a lower dose level. In addition, if accumulating tolerability, PK, or PD data suggest that the dose level in an expansion stage cohort is suboptimal for evaluation of anti-tumor activity, consideration will be given to enrolling new patients in that cohort at a different dose level. At no time will a dose level studied in the expansion stage exceed the highest dose level that has met escalation criteria in the dose-escalation stage.

[redacted]

Patients who do not receive GDC-6036 or discontinue GDC-6036 treatment before completing one cycle may be replaced. *Patients who have received KRAS G12C inhibitors prior to enrollment and for whom screening tissue and/or circulating tumor DNA assessments indicate a second KRAS mutation (in addition to KRAS G12C) following enrollment may be replaced, and the investigator will be notified of the detection of a second KRAS mutation.*

[redacted]

[redacted]

[redacted]

[redacted]

[redacted]

[redacted]

[redacted]

[redacted]

[redacted]

[redacted]

3.2 INTERNAL MONITORING COMMITTEE

An Internal Monitoring Committee (IMC) will be formed before enrollment into any Stage II expansion arm. At a minimum, the IMC will consist of the following Sponsor representatives associated with the study (Medical Monitor, safety scientist, biostatistician, and clinical pharmacologist), as well as a designated Sponsor medical oncologist not associated with the study who will be the IMC Chair. The IMC will operate according to a pre-specified charter; this charter will be made available upon request to the appropriate regulatory agencies. The IMC will periodically evaluate the accumulating safety data from all patients treated in this study and will make recommendations about the study conduct, as defined in the IMC charter.

3.3 END OF STUDY AND LENGTH OF STUDY

The end of this study is defined as the date when the last patient, last visit (LPLV), occurs. LPLV is expected to occur 12 months after the last patient is enrolled.

The total length of the study, from screening of the first patient to the end of the study, is expected to be approximately 42 months.

3.4 RATIONALE FOR STUDY DESIGN

3.4.1 Rationale for Patient Population

[redacted]

[redacted]

[redacted]

[redacted]

[redacted]

[redacted]

Stages I and II, Arm C (GDC-6036 in combination with cetuximab; dose escalation and dose expansion, respectively). This portion of the study will enroll patients with locally advanced, recurrent, or metastatic incurable *KRAS G12C*-positive CRC who have disease progression or intolerance to at least one prior chemotherapy regimen (e.g., FOLFOX, FOLFIRI, FOLFOXIRI ± bevacizumab). In addition, patients may have received prior *KRAS G12C* inhibitor therapy.

Based on mechanism of action and nonclinical data for GDC-6036 and other KRAS G12C inhibitors in development as monotherapy and in combination with anti-EGFR therapies, including data for combination therapy resulting in greater efficacy and overcoming resistance (GDC-6036 Investigator's Brochure; Amodio et al. 2020), the patient population has been selected to assess the safety and pharmacokinetics of GDC-6036 and cetuximab in patients who will provide safety information applicable to the patient population for future studies with this combination.

[redacted]

[redacted]

[redacted]

[redacted]

[redacted]

[redacted]

[redacted]

[redacted]

[redacted]

[redacted]

The enrollment of patients previously treated with a KRAS G12C inhibitor in the combination Arms C, [redacted] may be limited to enrich for KRAS G12C inhibitor-naive patients.

[redacted]

[redacted]

[redacted]

[redacted]

[redacted]

3.4.3 Rationale for Combining GDC-6036 with Other Anti-Cancer Therapies

[redacted]

[redacted]

[redacted]

[redacted]

3.4.3.2 Rationale for Combination Therapy with EGFR Inhibitors

GDC-6036, like other known KRAS G12C inhibitors, is a selective covalent inhibitor that locks KRAS G12C in its inactive GDP-bound state. Based on the mechanistic understanding of the RTK-RAS-MAPK pathway, it has been hypothesized that inhibition upstream of KRAS G12C with RTK inhibitors could potentially enhance KRAS G12C inhibition. Nonclinical studies in cell lines support this strategy by showing that EGFR inhibition, with either small molecules or anti-EGFR antibodies that inhibit the activity of wild-type EGFR, synergistically enhanced KRAS G12C inhibition (Lito et al. 2016; Canon et al. 2019; Amodio et al. 2020; Hallin et al. 2020). Possible mechanisms by which EGFR inhibition enhance the effect of KRAS G12C inhibitors include reducing nucleotide exchange to favor the GDP-bound state of KRAS G12C (Lito et al. 2016) and reducing the rebound enhancement of RTK signaling upon KRAS G12C inhibition (Amodio et al. 2020).

Combination with Cetuximab (Arm C)

In in vivo mouse studies, treatment of mice with CRC PDXs using the combination of GDC-6036 and cetuximab reduced tumor growth beyond that seen with GDC-6036 alone. Refer to the GDC-6036 Investigator's Brochure for more details.

Clinical validation of concomitant RTK-RAS-MAPK pathway inhibition in CRC has been shown by the approved use of the EGFR and BRAF inhibitors, cetuximab and encorafenib, to treat *BRAF V600E* colorectal carcinoma (Kopetz et al. 2019).

Arm C will build upon the strong nonclinical evidence showing synergy between EGFR inhibition and KRAS G12C inhibition in CRC and will investigate GDC-6036 in combination with cetuximab in patients with advanced or metastatic *KRAS G12C*-positive colorectal cancer (CRC) (GDC-6036 Investigator's Brochure; Amodio et al. 2020). The starting dose of cetuximab in combination with GDC-6036 will be an initial dose of 400 mg/m^2 as a 120-minute IV infusion on Day 1 followed by 250 mg/m^2 as a 60-minute IV infusion weekly, in 21-day cycles (Erbitux U.S. Package Insert; Erbitux E.U. SmPC; equivalent country-specific document). The combination of cetuximab and GDC-6036 is anticipated to have acceptable tolerability. Potential overlapping toxicities include gastrointestinal toxicities and elevated hepatic transaminases, and are expected

to be monitorable and manageable with supportive care and potentially dose modifications. *On the basis of nonclinical data available for GDC-6036 and clinical data from cetuximab, the Sponsor has assessed the benefit–risk profile of GDC-6036 in combination with cetuximab to be appropriate for initiating this first-in-human clinical study.*

[redacted]

[redacted]

[redacted]

[redacted]

[redacted]

[redacted]

[redacted]

[redacted]

[redacted]

[redacted]

[redacted]

[redacted]

[redacted]

[redacted]

[redacted]

[redacted]

[redacted]

[redacted]

3.4.6 Rationale for Dosing beyond Cycle 1

The ethical conduct of a study of cancer therapy requires that patients have the opportunity to continue study treatment provided that the treatment is active and tolerable, and that patients comply with the requirements of the protocol. Therefore, dosing beyond Cycle 1 will be allowed in the absence of unacceptable toxicity or clinically compelling disease progression, at the investigator's discretion, following a careful assessment and thorough discussion of the potential risks and benefits with the patient.

3.4.7 Rationale for PK [redacted] Sampling Schedule

The PK evaluation schedule is based on the predicted GDC-6036 PK profile in humans using physiologically based PK modeling of preclinical PK and in vitro hepatocyte turnover data. [redacted]

The frequent sampling schedule that follows a single dose of GDC-6036 is designed to capture data at a sufficient number of time points to provide a detailed profile of the absorption, distribution, and elimination of this drug (i.e., C_{max}, t_{max}, apparent terminal $t_{1/2}$, and AUC). PK sampling up to [redacted] hours postdose (approximately [redacted] is expected to allow for characterization of the complete PK profile.

Steady-state pharmacokinetics of GDC-6036 will be evaluated following the dose on Day 21. Additional plasma for PK estimation during Cycle 1 and at the start of subsequent cycles (Cycles ≥ 2) will be taken from blood samples drawn prior to dosing.

[redacted]

PK data obtained from patients in early cohorts will be used to determine whether GDC-6036 pharmacokinetics can be adequately characterized with the planned PK sampling schedule. If, upon analysis of these data, it is apparent that the pharmacokinetics of GDC-6036 cannot be adequately characterized with the planned PK sampling schedule, up to four additional samples may be collected from each patient, or

the PK sampling times may be adjusted (while keeping the total volume of blood drawn over 48 hours similar). Similarly, sampling time points that are not informative may be eliminated.

After the plasma samples are analyzed for GDC-6036 concentrations, any leftover samples may be used for exploratory metabolite profiling and identification, ex vivo protein binding, or development of PK or PD assays.

The PK sampling schedule for the [redacted] cetuximab, [redacted]) is based on their known time to C_{max} (30 minutes after the end of the infusion on Day 1 of Cycle 1) and C_{ss} or C_{min} (predose on Day 1 of Cycle 2 for all [redacted], and two additional predose assessments on Day 8 and Day 15 of Cycle 1 for cetuximab).

[redacted]

[redacted]

[redacted]

3.4.8 Rationale for Biomarker Assessments

Exploratory research on safety biomarkers may be conducted to support future drug development. Research may include further characterization of a safety biomarker or identification of safety biomarkers that are associated with susceptibility to developing adverse events or can lead to improved adverse event monitoring or investigation. Adverse event reports will not be derived from safety biomarker data by the Sponsor, and safety biomarker data will not be included in the formal safety analyses for this study. In addition, safety biomarker data will not inform decisions on patient management.

Clinical toxicity may not be a reliable surrogate of target modulation by GDC-6036. Therefore, PD biomarkers will be measured in tumor tissue to determine whether

clinically achievable exposures are sufficient for producing the desired effect on the intended molecular target.

[redacted] CRC, [redacted] are heterogeneous diseases. Therefore, the benefit from treatment with GDC-6036 may be variable among individuals. Predictive biomarkers will be assessed in an effort to identify those patients with *KRAS G12C*–positive tumors who are most likely to respond to GDC-6036. PD biomarkers will be assessed to demonstrate evidence of biologic activity of GDC-6036 in patients, to support selection of a recommended dose and dosing regimen, and to inform potential revisions to the PK sample collection schedule.

Blood samples will be collected at baseline, during the study, and at disease progression. Tumor tissue will be collected at baseline and, if deemed clinically feasible and with patients' consent, during the study and/or at the time of disease progression. Extraction of DNA, RNA, and protein from collected samples will enable exploratory analyses, including analysis to identify germline and/or somatic mutations that are predictive of response to study drug, are associated with progression to a more severe disease state, are associated with acquired resistance to study drug, are associated with susceptibility to developing adverse events, or can increase the knowledge and understanding of disease biology.

3.4.8.1 *KRAS G12C* Mutation Status from Tissue and Circulating Tumor DNA Assessments

Approximately [redacted], 4% of CRC, [redacted] harbor the *KRAS G12C* mutation. GDC-6036 is a potent and highly selective inhibitor that targets KRAS G12C, but not other mutations in KRAS, the wild-type form of KRAS, or other members of the RAS family. Therefore, only patients with tumors harboring the *KRAS G12C* mutation are eligible for this study. *KRAS* mutation status may be determined using the FoundationOne® CDx (F1CDx) assay, a U.S. Food and Drug Administration (FDA)-approved broad companion diagnostic (CDx) assay, FoundationOne® Liquid CDx (F1L CDx) assay, as well as other Sponsor-approved, FDA approved (FDA 2020), or well-validated laboratory developed tests performed in a Clinical Laboratory Improvement Amendments (CLIA)-validated or equivalently certified laboratory. Previous studies indicate that occurrence of the *KRAS G12C* mutation is an early event (Jamal-Hanjani et al. 2017), suggesting that analysis of archival tissue is a sufficient surrogate for selection of patients with *KRAS G12C*–positive tumors for GDC-6036 treatment.

3.4.8.2 Pharmacodynamic Pathway Modulation

GDC-6036 is a KRAS G12C inhibitor that suppresses downstream MAPK signaling by alkylation of KRAS G12C, thereby locking it in its inactive GDP-bound state. In nonclinical models, the level of KRAS G12C alkylation by GDC-6036 and the extent of MAPK pathway suppression correlate with response to GDC-6036. Pre-treatment and on-treatment tumor tissue collection will enable an assessment of the correlation of

MAPK pathway suppression and anti-tumor activity with GDC-6036 treatment. The extent of MAPK pathway suppression can be assessed using RNA analysis of MAPK target genes (e.g., *DUSP6*, *SPRY4*) or immunohistochemistry (IHC) analysis of phosphorylated downstream markers (e.g., pERK, pS6). In addition, on-treatment tumor tissue biopsies may enable direct assessment of the level of KRAS G12C alkylation by GDC-6036. The assessment of these PD biomarkers may inform future dose selection.

[redacted]

[redacted]

Plasma samples will also be collected to assess potential PD biomarkers that modulate signaling pathway activities.

3.4.8.3 Sequencing of Genes Related to Resistance to GDC-6036

DNA sequencing techniques, such as targeted next-generation sequencing (NGS) and whole exome sequencing, may offer a unique opportunity to identify biomarkers of response and/or resistance to GDC-6036. Sequencing of cancer-related genes may result in the identification of de novo and acquired mechanisms of resistance to GDC-6036.

3.4.8.4 Protein, RNA, and DNA Analysis

Evaluation of the signaling activities (e.g., MAPK, PI3K/AKT) in tumor cells and the immune activities (e.g., PD-L1) in the tumor microenvironment could provide valuable insights in the sensitivity or resistance to GDC-6036 treatment as a single agent or in combination therapy. [redacted]

In addition to mutational activation of proteins, expression levels of RNA or alterations in DNA may also modulate the activity of signaling pathways. RNA profiling of tumors will allow intrinsic subtyping of patients enrolled in the study. Analysis of the potential association between subtypes and patient outcome may identify subpopulations of patients who are most likely to respond to GDC-6036.

3.4.8.5 Plasma Sample for Somatic Tumor Mutation Analysis and Other Biomarkers

There is increasing evidence that cell-free DNA obtained from blood specimens of patients with cancer contains ctDNA, which is representative of the DNA and mutational status of cells in the tumor (Diehl et al. 2008; Maheswaran et al. 2008). Assays have been validated to detect cancer-related mutations (e.g., *KRAS*) from plasma. Results of these assays may be correlated with the mutational status determined from analysis of tumor specimens. The use of ctDNA to monitor response to treatment is an area of great interest, and could allow for an early, non-invasive, and quantifiable method for use in the clinical setting to identify candidates for specific therapies and monitoring of mutation status of the cancer over time (Wan et al. 2017). Analysis of ctDNA collected at various times during study treatment and after a patient progresses on GDC-6036 may help to identify mechanisms of response and acquired resistance to study treatment.

3.4.8.6 Blood Sample for Next-Generation Sequencing

Next-generation sequencing (NGS) technologies can generate a large quantity of sequencing data. Tumor DNA can contain both reported and unreported chromosomal alterations because of the tumorigenesis process. To help control for sequencing calls in previously unreported genomic alterations, a predose blood sample will be taken to determine whether the alteration is somatic.

3.4.8.7 Optional Tumor Biopsy Sample at the Time of Disease Progression

Understanding the mechanisms of resistance to KRAS G12C inhibitors is critical for the development of combination therapies and may provide an opportunity to develop next-generation inhibitors to prevent resistance. Notable examples include the *T790M* gatekeeper acquired mutation in *EGFR* in patients who progress on EGFR inhibitors and reactivation of the MAPK pathway in *BRAF*-mutant melanoma cancers that progress on BRAF inhibitors.

In all arms, tumor tissue may be collected at the time of disease progression to perform additional exploratory biomarker analyses. These analyses may include, but are not limited to DNA and RNA NGS or protein-based methods to assess cancer-related genes and biomarkers associated with common molecular and biological pathways.

3.4.8.8 Tumor Biopsy Sample for Patients Previously Treated with a KRAS G12C Inhibitor (Arms C, [redacted] [redacted])

Patients enrolled in Arm C (GDC-6036 with cetuximab), [redacted] who have previously been treated with a KRAS G12C inhibitor will consent to provide a recently acquired tumor specimen after completion of the last KRAS G12C inhibitor treatment, in addition to an archival tumor tissue specimen collected prior to treatment with any KRAS G12C inhibitor (e.g., diagnostic specimen). The analysis of these

samples will aid in determining resistance mechanisms to KRAS G12C inhibitors and potentially influence future therapies for patients who progress on a KRAS inhibitor.

3.4.9 Rationale for QT/QTc Assessments

The potential of non-cardiovascular drugs to delay cardiac repolarization, which may cause cardiac arrhythmias such as torsade de pointes, has emerged as a major concern for sponsors and regulators. Two International Council for Harmonisation (ICH) guidelines for nonclinical (S7B) and clinical (E14) testing have been developed.

Cardiovascular toxicity assessments were incorporated in nonclinical studies conducted with GDC-6036 on the basis of the testing strategy outlined in the ICH S6 and S7B guidelines. [redacted]

The QT/QTc assessment strategy for this trial was based on recommendations in the ICH E14 guideline and the special considerations for evaluating drug effects on the QT/QTc interval in patients with cancer (Fingert and Varterasian 2006). For patients undergoing intensive PK sampling, triplicate digital ECGs time-matched with PK sampling will be collected at baseline and postdose at 2 hours and 8 hours on Day 1 of Cycle 1 and Day 1 of Cycle 2 (Appendix 3). [redacted] Together, these assessments will allow an evaluation of the relationship between GDC-6036 exposure and changes in QT/QTc interval. Meal intake relative to dosing and other factors (e.g., supine position, ECGs collected before PK sampling) will be standardized, as it is well established that numerous external factors such as these may prolong the QT/QTc interval (Bednar et al. 2001).

The timing of ECG collections was based on the predicted GDC-6036 PK profile in humans using physiologically based PK modeling of preclinical PK and in vitro hepatocyte turnover data, and a typical small-molecule PK profile. The collection schedule should allow for an adequate characterization of the QT/QTc interval over a range of GDC-6036 concentrations. If the observed PK profile is different from the predicted PK profile, additional ECGs may be collected, or the ECG measurement times may be adjusted. Likewise, measurement times that are not informative will be eliminated.

3.4.10 Rationale for Optional FDG-PET Imaging

FDG-PET imaging has been shown to be a useful tool to identify changes associated with tumor activity (Weber 2006) and may provide an early readout of response in patients receiving GDC-6036 monotherapy or combination therapy. In addition, [redacted]

In patients who have consented to the optional FDG-PET imaging assessment, FDG-PET will be performed at baseline (up to 28 days prior to Cycle 1 Day 1) and between Days 10–16 of Cycle 1, prior to biopsy procedures whenever possible, if applicable. Please refer to Section 4.5.15 and the study imaging manual for additional details.

[redacted]

[redacted]

4. MATERIALS AND METHODS

4.1 PATIENTS

Approximately [redacted] patients with *KRAS G12C*–positive cancer, including but not limited to [redacted] CRC, will be enrolled in this study.

4.1.1 Inclusion Criteria

In addition to the general inclusion criteria in Section 4.1.1.1, patients must meet the criteria listed in Sections 4.1.1.2 through 4.1.1.8 for entry into specific cohorts.

4.1.1.1 General Inclusion Criteria

Patients must meet the following criteria for study entry:

- Signed Informed Consent Form
- Age ≥ 18 years at time of signing Informed Consent Form
- Ability to comply with the study protocol, in the investigator's judgment
- Evaluable or measurable disease per RECIST v1.1
- Eastern Cooperative Oncology Group (ECOG) performance status of 0 or 1
- Life expectancy of ≥12 weeks
- Adequate hematologic and organ function within 14 days prior to initiation of study treatment, defined by the following:
  - Absolute neutrophil count ≥1200/μL
  - Hemoglobin ≥9 g/dL
  - Platelet count ≥100,000/μL
  - Total bilirubin ≤1.5 × ULN
  - Serum albumin ≥2.5 g/dL
  - AST and ALT ≤2.5 × ULN with the following exception:

    Patients with documented liver metastases may have AST and/or ALT ≤5.0 × ULN.
  - Serum creatinine ≤1.5 × ULN or creatinine clearance ≥50 mL/min on the basis of the Cockcroft-Gault estimation:

$$\frac{(140 - \text{age}) \times (\text{weight in kg}) \times (0.85 \text{ if female})}{72 \times (\text{serum creatinine in mg/dL})}$$

  - INR < 1.5 × ULN and aPTT < 1.5 × ULN

    This applies only to patients who are not receiving therapeutic anticoagulation. Patients receiving therapeutic anticoagulation should be on a stable dose for at least 1 week prior to Cycle 1 Day 1.
- For women of childbearing potential: Agreement to remain abstinent (refrain from heterosexual intercourse) or use contraception, and agreement to refrain from donating eggs, as defined below:

  Women must remain abstinent or use contraceptive methods with a failure rate of < 1% per year and must refrain from donating eggs during the treatment period and after the final dose of study treatment for at least:

  - 6 months for GDC-6036
  - [redacted]
  - *2 months for cetuximab (Arm C)*
  - [redacted]

[redacted]

A woman is considered to be of childbearing potential if she is postmenarchal, has not reached a postmenopausal state (≥12 continuous months of amenorrhea with no identified cause other than menopause), and is not permanently infertile due to surgery (i.e., removal of ovaries, fallopian tubes, and/or uterus) or another cause as determined by the investigator (e.g., Müllerian agenesis). The definition of childbearing potential may be adapted for alignment with local guidelines or regulations.

Examples of contraceptive methods with a failure rate of < 1% per year include bilateral tubal ligation, male sterilization, hormonal contraceptives that inhibit ovulation, hormone-releasing intrauterine devices, and copper intrauterine devices.

The reliability of sexual abstinence should be evaluated in relation to the duration of the clinical trial and the preferred and usual lifestyle of the patient. Periodic abstinence (e.g., calendar, ovulation, symptothermal, or postovulation methods) and withdrawal are not adequate methods of contraception.
If required per local guidelines or regulations, locally recognized adequate methods of contraception and information about the reliability of abstinence will be described in the local Informed Consent Form.

- For men who are not surgically sterile: Agreement to remain abstinent (refrain from heterosexual intercourse) or use contraception, and agreement to refrain from donating sperm, as defined below:

  With a female partner of childbearing potential who is not pregnant, men must remain abstinent or use a condom plus an additional contraceptive method that together result in a failure rate of < 1% per year and must refrain from donating sperm during the treatment period and after the final dose of study treatment for at least:

  – 4 months for GDC-6036

  – *2 months for* cetuximab (Arm C*)*

  [redacted]

  With pregnant female partners, men must remain abstinent or use a condom to avoid exposing the embryo during the treatment period and after the final dose of study treatment for at least:

  – 4 months for GDC-6036

  – *2 months for* cetuximab (Arm C*)*

  [redacted]

- [redacted]
- [redacted]

The reliability of sexual abstinence should be evaluated in relation to the duration of the clinical trial and the preferred and usual lifestyle of the patient. Periodic abstinence (e.g., calendar, ovulation, symptothermal, or postovulation methods) and withdrawal are not adequate methods of contraception.
If required per local guidelines or regulations, locally recognized adequate methods of contraception and information about the reliability of abstinence will be described in the local Informed Consent Form.

- Confirmation of biomarker eligibility: Valid results from either central testing of blood or local testing of blood or tumor tissue documenting the presence of the *KRAS G12C* mutation

  Local testing of blood or tumor tissue must be performed using a Sponsor-approved validated polymerase chain reaction (PCR)-based or NGS assay performed at a CLIA or equivalently certified laboratory.

  Patients without available local test results for *KRAS G12C* mutation status must submit a blood sample to determine whether an eligible *KRAS G12C* mutation is present by the NGS-based FoundationOne® Liquid CDx (F1L CDx) Assay.

- Submission of a freshly collected pre-treatment blood sample, whether patients are enrolled by local or central test results for the *KRAS G12C* mutation
- Consent to provide fresh (preferred) or archival tumor tissue specimen

  It is preferred that the specimen be from the most recently collected and available tumor tissue (within 5 years), and whenever possible, from a metastatic site of disease. See the laboratory manual for instructions.

  For patients enrolled in Stage I, confirmation of available tumor tissue sample is required. For patients enrolled in Stage II, confirmation of shipment of tumor tissue is required.

- *For* patients with [redacted] and adenocarcinoma of the colon or rectum: *patients* must not have a known concomitant second oncogenic driver [redacted] *BRAF V600E* mutation, *ERBB2* amplification) as determined by the Foundation Medicine, Inc. (FMI) NGS assay or by a Sponsor-approved validated PCR-based or NGS assay performed at a local CLIA-certified or equivalently-certified laboratory
- *For patients requiring accessible lesion(s) that permit a total of at least two biopsies (pre-treatment and on-treatment) without unacceptable risk of a significant procedural complication:*

  In lieu of a fresh pre-treatment biopsy, a recently obtained biopsy performed after completion of the last anti-cancer therapy will be acceptable.

Acceptable samples include core needle biopsies for deep tumor tissue or lymph nodes or excisional, incisional, punch, or forceps biopsies for cutaneous, subcutaneous, or mucosal lesions. Fine needle aspirates, cell pellets from effusions or ascites, lavage samples, and bone biopsies are not permitted. Target lesions considered for core needle biopsies should be deemed suitable for retrieval of at least three cores at a given time point (minimum diameter 18-gauge, if feasible).

If multiple lesions are available, it is preferable to obtain the on-treatment biopsy from the same lesion (or organ) as the pre-treatment biopsy, if feasible, to avoid introduction of heterogeneity related to site of metastasis.

[redacted]

[redacted]

[redacted]

[redacted]

[redacted]

[redacted]

[redacted]

[redacted]

- [redacted]
    - [redacted]
    - [redacted]

        [redacted]
- [redacted]
    - [redacted]

        [redacted]
- [redacted]
    - [redacted]

        [redacted]
    - [redacted]

[redacted]

4.1.1.6 Stage I and Stage II Arm C (Cetuximab Combination)

- Histologically documented, locally advanced, recurrent, or metastatic incurable adenocarcinoma of the colon or rectum

  Patients with appendiceal tumors are excluded.

  Patients must have experienced disease progression or intolerance to at least one prior chemotherapy regimen (e.g., FOLFOX, FOLFIRI, FOLFOXIRI ± bevacizumab).

  If a patient that has progressed after at least one available standard therapy has additional approved standard treatment options available, the study doctor must *offer these additional approved treatment options and* discuss the risks and benefits of those treatments before informed consent to participate in this study is obtained. This discussion must be documented in patient records.

- For patients who have previously been treated with a KRAS G12C inhibitor, consent to provide the following:
  - An archival tumor tissue specimen collected prior to treatment with the KRAS G12C inhibitor (e.g., diagnostic specimen)
  - A recently acquired tumor specimen after completion of the last KRAS G12C inhibitor treatment

[redacted]

[redacted]

[redacted]

[redacted]

[redacted]

[redacted]

[redacted]

[redacted]

[redacted]

- [redacted]

[redacted]

[redacted]

[redacted]

[redacted]

[redacted]

- [redacted]
  - [redacted]
  - [redacted]
    - [redacted]

4.1.2 Exclusion Criteria

In addition to the general exclusion criteria in Section 4.1.2.1, patients who meet any of the criteria listed in Sections 4.1.2.2 through 4.1.2.5 will be excluded from entry into specific cohorts.

4.1.2.1 General Exclusion Criteria

Patients who meet any of the following criteria will be excluded from study entry:

- Inability or unwillingness to swallow pills
- Inability to comply with study and follow-up procedures
- Malabsorption syndrome or other condition that would interfere with enteral absorption
- Known and untreated, or active central nervous system (CNS) metastases (progressing or requiring anticonvulsants or corticosteroids for symptomatic control)

  Patients with a history of treated CNS metastases are eligible, provided they meet all of the following criteria:

  - Measurable or evaluable disease outside the CNS
  - No history of intracranial hemorrhage or spinal cord hemorrhage
  - No ongoing requirement for corticosteroids as therapy for CNS metastases, with corticosteroids discontinued for ≥2 weeks prior to enrollment and no ongoing symptoms attributed to CNS metastases
  - No stereotactic radiation within 7 days or whole-brain radiation within 14 days prior to Day 1 of Cycle 1
  - No evidence of interim progression between the completion of CNS-directed therapy and the screening radiographic study
  - Note: Patients with new asymptomatic CNS metastases detected at screening are eligible for the study after receiving radiotherapy and/or surgery. Following treatment, these patients may be eligible without the need to repeat the additional brain scan, if all other criteria are met.
- Leptomeningeal disease or carcinomatous meningitis
- Uncontrolled pleural effusion, pericardial effusion, or ascites requiring recurrent drainage procedures biweekly or more frequently

  Indwelling pleural or abdominal catheters may be allowed, provided the patient has adequately recovered from the procedure, is hemodynamically stable and symptomatically improved, and after discussion with the Medical Monitor.

- Any active infection that, in the opinion of the investigator, could impact patient safety, or serious infection requiring IV antibiotics within 7 days prior to Day 1 of Cycle 1

  In the setting of a pandemic or epidemic, screening for active infections should be considered according to local or institutional guidelines or those of applicable professional societies (e.g., American Society of Clinical Oncology or European Society for Medical Oncology).

- Clinically significant history of liver disease, including viral or other hepatitis, current alcohol abuse, or cirrhosis
- Known HIV infection

  [redacted]

- Any other diseases, active or uncontrolled pulmonary dysfunction, metabolic dysfunction, physical examination finding, or clinical laboratory finding giving reasonable suspicion of a disease or condition that contraindicates the use of an investigational drug, that may affect the interpretation of the results, or renders the patients at high risk from treatment complications
- Uncontrolled hypercalcemia (> 1.5 mmol/L ionized calcium or calcium > 12 mg/dL or corrected serum calcium ≥ ULN) or symptomatic hypercalcemia requiring continued use of bisphosphonate therapy or denosumab
- Significant traumatic injury or major surgical procedure within 4 weeks prior to Day 1 of Cycle 1
- Patients with chronic diarrhea, short bowel syndrome or significant upper gastrointestinal surgery including gastric resection, a history of inflammatory bowel disease (e.g., Crohn's disease or ulcerative colitis) or any active bowel inflammation (including diverticulitis)
- Prior treatment with any KRAS G12C inhibitor, with the following exceptions:

  Patients in Arm C, [redacted] only may have had prior KRAS G12C inhibitor treatment. *These patients must not have discontinued prior KRAS G12C inhibitor treatment because of intolerance or toxicity assessed as related to the prior KRAS G12C inhibitor.*

- Treatment with chemotherapy, immunotherapy, biologic therapy *or an investigational agent* as anti-cancer therapy within 3 weeks *or five half-lives* prior to initiation of study treatment*, whichever is shorter*, or endocrine therapy within 2 weeks prior to initiation of study treatment, except for the following:

  Hormonal therapy with gonadotropin-releasing hormone (GnRH) agonists or antagonists for endocrine sensitive cancers (e.g., prostate, endometrial, hormone receptor-positive breast cancer)

  Kinase inhibitors, approved by regulatory authorities, may be used up to 2 weeks prior to initiation of study treatment, provided any drug-related toxicity has completely resolved and after discussion with the Medical Monitor.

- Radiation therapy (other than palliative radiation to bony metastases and radiation to CNS metastases as described above) as cancer therapy within 4 weeks prior to initiation of study treatment
- Palliative radiation to bony metastases within 2 weeks prior to initiation of GDC-6036
- Adverse events from prior anti-cancer therapy that have not resolved to Grade ≤ 1 except for alopecia, vitiligo, endocrinopathy managed with replacement therapy, or Grade ≤ 2 peripheral neuropathy
- History of other malignancy within 5 years prior to screening, with the exception of patients with a negligible risk of metastasis or death and/or treated with expected curative outcome (such as appropriately treated carcinoma in situ of the cervix, non-melanoma skin carcinoma, localized prostate cancer, ductal carcinoma in situ, or Stage I uterine cancer)
- History of or active clinically significant cardiovascular dysfunction, including the following:
  - History of stroke or transient ischemic attack within 6 months prior to first dose of study treatment
  - History of myocardial infarction within 6 months prior to first dose of study treatment
  - New York Heart Association Class III or IV cardiac disease or congestive heart failure requiring medication (see Appendix 6)
  - Uncontrolled arrhythmias, history of or active ventricular arrhythmia requiring medication
  - Coronary heart disease that is symptomatic or unstable angina
  - Congenital long QT syndrome or QT interval corrected through use of Fridericia's formula (QTcF) >470 ms demonstrated by at least two ECGs >30 minutes apart, or family history of sudden unexplained death or long QT syndrome
  - Current treatment with medications that are well known to prolong the QT interval
- Pregnant or breastfeeding, or intending to become pregnant during the study or within 6 months after the final dose of GDC-6036

  Women of childbearing potential (including those who have had a tubal ligation) must have a negative serum pregnancy test result within 14 days prior to initiation of study drug.
- *Known hypersensitivity to any of the components of GDC-6036*

[redacted]

- [redacted]

4.1.2.3 Stage I and Stage II Arm C (Cetuximab Combination)

- History of idiopathic pulmonary fibrosis, organizing pneumonia (e.g., bronchiolitis obliterans), drug-induced pneumonitis, or idiopathic pneumonitis, or evidence of active pneumonitis on screening chest computed tomography (CT) scan

  History of radiation pneumonitis in the radiation field (fibrosis) is permitted.

- Known hypersensitivity to any of the components of cetuximab

[redacted]

[redacted]

4.2 METHOD OF TREATMENT ASSIGNMENT

This is a non-randomized, open-label study. Each patient will be assigned to a cohort in the order in which he or she is enrolled.

After initial written informed consent has been obtained, all screening procedures and assessments have been completed, and eligibility has been established for a patient, the study site will obtain the patient's identification number and treatment assignment from an interactive voice or web-based response system (IxRS).

4.3 STUDY TREATMENT AND OTHER TREATMENTS RELEVANT TO THE STUDY DESIGN

The investigational medicinal products (IMPs) for this study are GDC-6036, [redacted] cetuximab, [redacted]

The reference safety information (RSI) document for each IMP is as follows:

- GDC-6036: Investigator's Brochure
- [redacted]
- Cetuximab: *E.U.* Summary of Product Characteristics
- [redacted]

4.3.1 Study Treatment Formulation, Packaging, and Handling

4.3.1.1 GDC-6036

[redacted]

[redacted]

[redacted]

4.3.1.3 **Cetuximab**

Cetuximab will be supplied by the Sponsor in commercially available formulations, or supplied by the study sites and reimbursed by the Sponsor. For information on the formulation and handling of cetuximab, see the local prescribing information for cetuximab.

[redacted]

[redacted]

[redacted]

4.3.2 <u>Study Treatment Dosage, Administration, and Compliance</u>

4.3.2.1 GDC-6036

[redacted]

[redacted]

[redacted]

[redacted]

In Stage I [redacted] *Stage I combination arms (Arms* [redacted] *C,* [redacted] *),* and Stage II expansion cohorts *(Arms* [redacted] *C,* [redacted] QD dosing of GDC-6036 will begin on Day 1 of Cycle 1 and each cycle will be 21 days in length.

For GDC-6036 doses to be administered at home, a sufficient number of capsules [redacted] should be dispensed to the patient to last until the next visit, or at the investigator's discretion, through one cycle. Patients will self-administer GDC-6036 as detailed below, except on study visit days when GDC-6036 will be administered in the clinic.

Patients should take GDC-6036 at approximately the same time each day unless otherwise instructed. Patients will be instructed as to the number and strength of capsules [redacted] to take, according to their assigned dose level and schedule. Patients will be asked to record the time and date that they take each dose in a medication diary.

Unless otherwise instructed, GDC-6036 should be taken on an empty stomach, i.e., food should be avoided at least 2 hours before as well as 1 hour after the dose is administered*,* [redacted] There are no restrictions on water intake. Importantly, GDC-6036 capsules [redacted] will be swallowed whole (not chewed) with a minimum of 240 mL (8 fluid ounces) of water.

[redacted]

[redacted]

[redacted]

[redacted]

Refer to the pharmacy manual for detailed instructions on drug preparation, storage, and administration.

Any dose modification should be noted on the Study Drug Administration electronic Case Report Form (eCRF). Cases of overdose, medication error, drug abuse, or drug misuse, along with any associated adverse events, should be reported as described in Section 5.3.5.11.

If a patient misses any dose of GDC-6036 or vomits up a capsule [redacted], he or she should be instructed to skip that dose, document it in patient's drug diary, and resume dosing with the next scheduled dose. Missed doses will not be made up.

Patients will be instructed to bring their medication diary to each study visit for assessment of compliance. Patients will also be instructed to bring all unused capsules [redacted] to each study visit or, at the investigator's discretion, at the end of each cycle, for drug accountability.

Guidelines for dosage modification and treatment interruption or discontinuation for patients who experience adverse events are provided in Section *5.1.7.1*. Guidelines for intra-patient dose escalation are provided in Section 3.1.1.6 *(Stage I) and Section* 3.1.2.2 *(Stage II Arm A4).*

[redacted]

[redacted]

[redacted]

[redacted]

[redacted]	[redacted]
[redacted]	[redacted]

[redacted]

[redacted]

[redacted]

[redacted]

4.3.2.3 Cetuximab

Cetuximab will be administered at an initial dose of 400 mg/m^2 as a 120-minute IV infusion on Day 1 followed by 250 mg/m^2 as a 60-minute IV infusion weekly, in 21-day cycles. The maximum infusion rate must not exceed *5 mg/min for the initial administration and* 10 mg/min *for the subsequent administrations.* Cetuximab should be administered following administration of GDC-6036.

On PK collection days (see Appendix 3), the start of the cetuximab administration should be 30 minutes after the administration of GDC-6036.

Administration of cetuximab will be performed in a monitored setting where there is immediate access to trained personnel and adequate equipment and medicine to manage potentially serious reactions. For anaphylaxis precautions, see *Appendix 13*. Guidelines for medical management of cetuximab infusion-related reactions (IRRs) are provided in Appendix 9. The investigator must ensure that the intended premedication is suitable for participants according to national prescribing information. Prior to the first infusion, participants must receive premedication with an antihistamine and a corticosteroid. This premedication is recommended prior to all subsequent infusions (see local prescribing information for cetuximab [Erbitux U.S. Package Insert; Erbitux E.U. SmPC; or equivalent country-specific document]). Close monitoring is required during the infusion and for at least 1 hour after the end of the infusion.

Cetuximab dose may be reduced when deemed necessary. Guidelines for dosage modification and treatment interruption or discontinuation for patients who experience adverse events are provided in Appendix 9.

[redacted] [redacted]

[redacted]

[redacted]

[redacted]

[redacted]

[redacted]

[redacted]

[redacted]

[redacted]

[redacted]

4.3.3 Investigational Medicinal Product Handling and Accountability

All IMPs required for completion of this study will be provided by the Sponsor. The study site (i.e., investigator or other authorized personnel [e.g., pharmacist]) is responsible for maintaining records of IMP delivery to the site, IMP inventory at the site, IMP use by each patient, and disposition or return of unused IMP, thus enabling reconciliation of all IMP received, and for ensuring that patients are provided with doses specified by the protocol.

The study site should follow all instructions included with each shipment of IMP. The study site will acknowledge receipt of IMPs supplied by the Sponsor using the IxRS to confirm the shipment condition and content. Any damaged shipments will be replaced. The investigator or designee must confirm that appropriate temperature conditions have been maintained during transit*, either by time monitoring (shipment arrival date and time) or temperature monitoring,* for all IMPs received and that any discrepancies have been reported and resolved before use of the IMPs. All IMPs must be stored in a secure, environmentally controlled, and monitored (manual or automated) area in accordance with the labeled storage conditions, with access limited to the investigator and authorized staff.

Only patients enrolled in the study may receive IMPs, and only authorized staff may supply or administer IMPs.

IMPs will either be disposed of at the study site according to the study site's institutional standard operating procedure or be returned to the Sponsor with the appropriate documentation. The site's method of destroying Sponsor-supplied IMPs must be agreed to by the Sponsor. The site must obtain written authorization from the Sponsor before any Sponsor-supplied IMP is destroyed, and IMP destruction must be documented on the appropriate form.

Accurate records of all IMPs received at, dispensed from, returned to, and disposed of by the study site should be recorded on the drug accountability log.

Refer to the pharmacy manual and/or the GDC-6036 Investigator's Brochure for information on IMP handling, including preparation and storage, and accountability.

[redacted] [redacted]

[redacted]

[redacted]

- [redacted]
- [redacted]
- [redacted]

[redacted]

- [redacted]
- [redacted]
- [redacted]
- [redacted]

[redacted]

[redacted]

4.4 CONCOMITANT THERAPY, PROHIBITED FOOD, AND ADDITIONAL RESTRICTIONS

Concomitant therapy consists of any medication (e.g., prescription drugs, over-the-counter drugs, vaccines, herbal or homeopathic remedies, nutritional supplements) used by a patient in addition to protocol-mandated treatment from 7 days prior to initiation of study drug to the study treatment completion. All such medications should be reported to the investigator and recorded on the Concomitant Medications eCRF.

4.4.1 Permitted Therapy

Patients who experience toxicities should be treated symptomatically as clinically indicated.

Patients treated with anti-seizure medications or warfarin should have medication levels or appropriate laboratory tests monitored regularly.

Patients who use oral contraceptives or other allowed maintenance therapy as specified in the eligibility criteria (see Sections 4.1.1 and 4.1.2) should continue their use.

Anti-emetic *medications should not be administered prophylactically before initial treatment with study drug during Stage I DLT assessment. Anti*-diarrheal medications should not be administered prophylactically before initial treatment with study drug. At the discretion of the investigator, prophylactic anti-emetic and anti-diarrheal medication(s) may be used as per standard clinical practice before subsequent doses of study drug.

Pain medications administered per standard clinical practice are acceptable while the patient is enrolled in the study.

Bisphosphonate and denosumab therapy for bone metastases or osteopenia/osteoporosis is allowed. Patients on oral bisphosphonate therapy should be without signs of esophagitis.

Multivitamins, calcium, and vitamins C, D, and E supplements are allowed. However, due to the potential for drug-supplement interactions, and variability among suppliers and batches, the use of other dietary supplements is cautioned and should be discussed with the Medical Monitor.

4.4.2 Cautionary Therapy

4.4.2.1 Medications Given with Precaution due to Effects Related to GDC-6036

[redacted]

benefits should be discussed with the Medical Monitor prior to concomitant [redacted]

- [redacted]
- [redacted]

[redacted]

The above lists of medications are not necessarily comprehensive. The investigator should consult the prescribing information when determining whether a concomitant medication can be safely administered with study treatment. In addition, the investigator should contact the Medical Monitor if questions arise regarding medications not listed above.

[redacted]

[redacted]

[redacted]

4.4.2.3 Medications Given with Precaution in Arm C (GDC-6036 in Combination with Cetuximab)

Please refer to local prescribing information for cetuximab (Erbitux U.S. Package Insert; Erbitux E.U. SmPC; equivalent country-specific document).

[redacted]

[redacted]

[redacted]

[redacted]

[redacted]

[redacted]

[redacted]

[redacted]

[redacted]

[redacted]

[redacted]

[redacted] [redacted]

[redacted]

[redacted]

4.4.2.7 Herbal Therapies

Concomitant use of herbal therapies is not recommended because their pharmacokinetics, safety profiles, and potential drug–drug interactions are generally unknown.

4.4.3 Prohibited Therapy

4.4.3.1 Prohibited Therapy for All Patients

Use of the following concomitant therapies is prohibited during the study and for at least 7 days prior to initiation of study treatment, unless specified below:

- Investigational therapy (other than protocol-mandated study treatment) is prohibited within 3 weeks or five half-lives prior to initiation of study treatment, whichever is shorter, and during study treatment.

- Concomitant therapy intended for the treatment of cancer whether approved by the FDA or experimental, including chemotherapy, radiotherapy, immunotherapy, biologic therapy, herbal therapy, or hormonal therapy except for the following:

  Hormonal therapy with gonadotropin-releasing hormone (GnRH) agonists or antagonists for endocrine sensitive cancers (e.g., prostate, endometrial, hormone receptor-positive breast cancer) is allowed.

  Hormone replacement therapy or oral contraception is allowed.

- Radiotherapy for unequivocal progressive disease with the exception of new brain metastases in the setting of systemic response as follows: patients who have demonstrated control of their systemic disease (defined as having received clinical benefit [i.e., a PR, CR, or SD for ≥3 months]), but who have developed brain metastases that are treatable with radiation, will be allowed to continue to receive therapy with GDC-6036 during the study until they either experience systemic progression of their disease and/or further progression in the brain (based on investigator assessments). Patients should not miss more than one cycle of study treatment due to radiation treatment and must meet eligibility requirements to continue on study treatment (Section 4.1.1). After Cycle 1 in Stage I Arm A or for all cycles in Stage II, certain forms of radiation therapy may be considered for pain palliation, if patients are deriving benefit. Study treatment may be suspended during radiation therapy with agreement by the Medical Monitor.

- Quinidine or other anti-arrhythmic agents

- Initiation or increased dose of hematopoietic colony-stimulating factors (CSFs; e.g., granulocyte CSF; filgrastim, granulocyte/macrophage CSF; sargramostim, pegfilgrastim, erythropoietin, darbepoetin, and thrombopoietin) from 7 days before Cycle 1, Day 1 until completion of the DLT assessment window in the absence of a DLT (Stage I Arm A)

  After the DLT assessment window has been completed (or after DLT has been observed), CSFs may be administered per standard clinical practice.

Patients who require the use of any prohibited therapies will be discontinued from study treatment and followed for safety outcomes for a treatment-specific period after their final dose of study treatment or until they receive another anti-cancer therapy, whichever occurs first (see Section 5.3.1).

[redacted]

[redacted]

[redacted]

- [redacted]
- [redacted]

[redacted]

[redacted]

[redacted]

4.4.4 Prohibited Food

Use of the following foods is prohibited as described below:

- Consumption of grapefruit, grapefruit juice, *pomelo,* or Seville oranges (potent CYP3A4 enzyme inhibitors) is prohibited for at least 3 days prior to initiation of study treatment and during study treatment.

4.4.5 Additional Restrictions

From screening until completion of the study, patients will drink no more than two servings of alcohol per day (one serving is 12 ounces of beer, 5 ounces of wine, 1.5 ounces of spirits, or equivalent). Patients must agree to abstain from alcohol consumption 12 hours prior to Day 1.

4.5 STUDY ASSESSMENTS

The schedules of activities to be performed during the study are provided in Appendix 1 through Appendix 3.

All activities should be performed and documented for each patient.

All patients will be closely monitored for safety and tolerability throughout the course of therapy and at the study treatment discontinuation visit. Patients should be assessed for toxicity prior to each dose; dosing will occur only if the clinical assessment and local laboratory test values are acceptable.

Every effort should be made to adhere to the protocol-mandated visits and procedures. For patients receiving study treatment beyond the DLT assessment window, if the timing of a protocol-mandated visit coincides with a holiday and/or other scheduling conflict that

causes the visit to fall outside of the protocol-defined visit windows, the visit should be scheduled on the nearest date, which may be earlier or later than the originally scheduled visit.

Collection of any non-safety-related data or patient samples may be terminated by the Sponsor at any time if further collection of such data or samples is not related to the study's primary objective(s). Discontinuation of any such data collection will be communicated to investigators by means of a memorandum and will not require a protocol amendment.

4.5.1 Informed Consent Forms and Screening Log

Written informed consent for participation in the study must be obtained before performing any study-related procedures (including screening evaluations). Informed Consent Forms for enrolled patients and for patients who are not subsequently enrolled will be maintained at the study site.

All screening evaluations must be completed and reviewed to confirm that patients meet all eligibility criteria before enrollment. The investigator will maintain a screening log to record details of all patients screened and to confirm eligibility or record reasons for screening failure, as applicable.

4.5.2 Medical History, Baseline Conditions, Concomitant Medication, and Demographic Data

Medical history, including clinically significant diseases, surgeries, cancer history (including prior tumor characteristics, cancer therapies and procedures), reproductive status, smoking history and use of alcohol and drugs of abuse, will be recorded at baseline. In addition, all medications (e.g., prescription drugs, over-the-counter drugs, vaccines, herbal or homeopathic remedies, nutritional supplements) used by the patient within 7 days prior to initiation of study treatment will be recorded. At the time of each follow-up physical examination, an interval medical history should be obtained and any changes in medications and allergies should be recorded.

Demographic data will include age, sex, and self-reported race/ethnicity. Demographic data may be collected on all patients who undergo screening, including those who screen fail.

4.5.3 Physical Examinations

A complete physical examination, performed at screening and other specified visits, should include an evaluation of the head, eyes, ears, nose, and throat, and the cardiovascular, dermatologic, musculoskeletal, respiratory, gastrointestinal, genitourinary, and neurologic systems. Any abnormality identified at baseline should be recorded on the General Medical History and Baseline Conditions eCRF.

Limited, symptom-directed physical examinations should be performed at specified postbaseline visits and as clinically indicated. Changes from baseline abnormalities should be recorded in patient notes. New or worsened clinically significant abnormalities should be recorded as adverse events on the Adverse Event eCRF.

4.5.4 Vital Signs

Vital signs will include measurements of respiratory rate, pulse rate, and systolic and diastolic blood pressure while the patient is in a seated position, and temperature. In addition, oxygen saturation will be measured at the screening visit. Record abnormalities observed at baseline on the General Medical History and Baseline Conditions eCRF. At subsequent visits, record new or worsened clinically significant abnormalities on the Adverse Event eCRF.

4.5.5 Tumor and Response Evaluations

Imaging studies will include a computed tomography (CT) scan of the chest, abdomen, and pelvis. A bone scan or other institutional standard bone imaging to assess for extent of disease should be performed as clinically indicated. At screening, a CT scan of the neck and CT or magnetic resonance imaging (MRI) brain should be obtained if clinically indicated *or if the neck or brain are known sites of disease.*

All known sites of disease must be documented at screening and re-assessed at each subsequent tumor evaluation. A documented standard-of-care tumor assessment performed within 28 days prior to Cycle 1 Day 1 may be used for the required screening assessment provided it meets the above requirements.

Response will be assessed by the investigator on the basis of physical examinations, computed tomography (CT) scans, and other modalities as clinically indicated, which may include brain imaging (CT or MRI), MRI scans, or bone scan, using RECIST v1.1 criteria (Appendix 5). An objective response should be confirmed by repeat assessments ≥4 weeks after initial documentation. The same radiographic procedure used to assess disease sites at screening should be used throughout the study (e.g., the same contrast protocol for CT scans). Assessments should be performed by the same evaluator to ensure internal consistency across visits whenever possible.

At the investigator's discretion, CT scans may be repeated at any time if progressive disease is suspected.

4.5.6 Laboratory, Biomarker, and Other Biological Samples

Samples for the following laboratory tests will be sent to the study site's local laboratory for analysis:

- Hematology: WBC count, RBC count, hemoglobin, hematocrit, reticulocyte count, platelet count, differential count (neutrophils, eosinophils, basophils, monocytes, lymphocytes, other cells)

- Chemistry panel (serum or plasma): bicarbonate or total carbon dioxide (if considered standard of care for the region), sodium, potassium, chloride, glucose, BUN or urea, creatinine, total protein, albumin, phosphate, calcium, magnesium, total and direct bilirubin, ALP, ALT, and AST
- Lipid profile: total cholesterol, HDL, LDL, and triglycerides
- Amylase and lipase
- Coagulation: INR, aPTT, PT
- [redacted]

[redacted]

- Urinalysis, including dipstick (pH, specific gravity, glucose, protein, ketones, blood, leukocyte esterase, nitrite) and microscopic examination (sediment, RBCs, WBCs, casts, crystals, epithelial cells, bacteria)

[redacted]

- Pregnancy test

  All women of childbearing potential will have a serum pregnancy test at screening and serum or urine pregnancy tests performed at specified subsequent *timepoints. Following discontinuation of study treatment, patients will undergo monthly pregnancy testing for a treatment-specific period after the final dose of study drug or initiation of another anti-cancer therapy, whichever occurs first:*

  – [redacted]

  [redacted]

  – *Arm C: the later of approximately 1 month after the final dose of GDC-6036 or approximately 2 months after the final dose of cetuximab*

  – [redacted]

  Refer to Appendix 1 and Appendix 2.

The following samples will be sent to one or several central laboratories or to the Sponsor or a designee for analysis:

- Blood sample for *KRAS G12C* mutation status
- Plasma samples for PK, including analysis of GDC-6036 and potential circulating metabolites, and ex vivo plasma protein binding (see Appendix 3)
- [redacted]
- *Serum samples for PK/ADA for* [redacted] *cetuximab, and* [redacted]
- Plasma samples for biomarkers (somatic tumor mutation analysis of ctDNA and biomarkers of pathway modulation)

  Plasma samples will be collected at pre-defined timepoints during study treatment (see Appendix 3) and at the study treatment discontinuation visit for exploratory biomarker research.
- Blood samples for whole genome sequencing (WGS)

  Unless prohibited by the local regulatory authority, gene mutations will be assayed by WGS or other acceptable methodology such as multiplex PCR or allele-specific PCR. Results may be correlated to study treatment or other clinical measures to better understand the impact of genetic variants on drug metabolism, exposure, adverse events, and/or response.
- Blood samples for NGS (DNA and RNA)

- Archival or freshly collected (if archival is not available) tumor tissue sample obtained prior to treatment for determination of *KRAS G12C* mutation status and for exploratory research on biomarkers and biomarker assay development. *Refer to Section 4.5.11.*
- ***For patients*** **enrolled in Arm C, [redacted] who have previously been treated with a KRAS G12C inhibitor** ***only***: an archival tumor tissue specimen collected prior to treatment with any KRAS G12C inhibitor (e.g., diagnostic specimen) and a recently acquired tumor specimen after completion of the last KRAS G12C inhibitor treatment. *Refer to Section 4.5.11.*
- [redacted]
- ***For patients who consent to optional tumor biopsy at the time of disease progression only:*** *freshly collected on-treatment tumor biopsies. Refer to Section 4.5.13.*

Exploratory biomarker research may include, but will not be limited to, the following: KRAS G12C alkylation by GDC-6036, protein expression, copy number and mutation analysis of cancer-related genes, transcriptional analysis of KRAS/MAPK target genes (e.g., *DUSP6, SPRY4*), IHC analysis of phosphorylated *RTK/MAPK* markers (e.g., pERK, pS6, [redacted]) and related biomarkers (e.g., Ki67), gene expression signatures associated with disease biology, immune-related tumor microenvironment cancer signaling (e.g., PD-L1), analysis of ctDNA, expression of soluble, systemic cytokines and chemokines, analysis of potential predictive biomarkers and mechanisms of resistance, analysis of cytomegalovirus, human herpesvirus, and/or Epstein-Barr virus status, [redacted]

Exploratory biomarker research may involve DNA, ctDNA, protein or RNA extraction, analysis of somatic mutations, and use of WES or NGS.

NGS may be performed by FMI. If performed by FMI, the investigator may obtain an NGS report through Foundation Medicine's web portal. If allowed by local laws, the investigator may share and discuss the results with the patient, unless the patient chooses otherwise. The NGS report is generated for research purposes and is not provided for the purpose of guiding future treatment decisions. Results will not be available for samples that do not meet criteria for testing.

The investigator may obtain results through Foundation Medicine's web portal for the following:

- FMI testing performed on the blood sample submitted during screening for biomarker eligibility
- FMI testing performed on tissue submitted at the time of disease progression

Screening blood and tissue samples, including those collected from patients who do not enroll in the study, may be used for future research and/or development of diagnostic tests, which may allow for individualized drug therapy for patients in the future.

For sampling procedures, storage conditions, and shipment instructions, see the laboratory manual.

Unless the patient gives specific consent for his or her leftover samples to be stored for optional exploratory research (see Section 4.5.17), biological samples will be destroyed no later than the time of completion of the final Clinical Study Report, with the following exceptions:

- Plasma or serum samples collected for PK and [redacted] analyses may be needed for circulating metabolite profiling or for ex vivo determination of plasma protein binding or for additional exploratory PK or pharmacogenomics characterization; therefore, these samples will be destroyed no later than 5 years after the final Clinical Study Report has been completed.
- Blood, plasma, and tumor tissue samples ([redacted], if the patient gives specific consent; see Section 4.5.14) collected for biomarker research and biomarker assay development will be destroyed no later than 15 years after the final Clinical Study Report has been completed, or until they are exhausted. However, the storage period will be in accordance with the IRB/EC-approved Informed Consent Form and applicable laws (e.g., health authority requirements).
- For enrolled patients, remaining archival tissue blocks will be returned to the site upon request or no later than the time of final closure of the study database, whichever occurs first. For patients who are not enrolled, remaining archival tissue blocks will be returned to the site after eligibility determination.

When a patient withdraws from the study, samples collected prior to the date of withdrawal may still be analyzed, unless the patient specifically requests that the samples be destroyed or local laws require destruction of the samples. However, if samples have been tested prior to withdrawal, results from those tests will remain as part of the overall research data.

Data arising from sample analysis, including data on germline variants, will be subject to the confidentiality standards described in Section 8.4.

Given the complexity and exploratory nature of exploratory biomarker analyses, data derived from these analyses will generally not be provided to study investigators or patients unless required by law. The aggregate results of any conducted research will be available in accordance with the effective Sponsor policy on study data publication.

4.5.7 Electrocardiograms

Triplicate ECG recordings will be obtained at specified timepoints, as outlined in the schedule of activities (see Appendix 3). ECGs acquired on different days should be as

closely time-matched as feasible. Three interpretable ECG recordings (e.g., without artifacts) must be obtained at each timepoint (±5 minutes). The average of the three readings will be used to determine ECG intervals (e.g., PR, QRS, QT).

Single ECG recordings may be obtained at unscheduled timepoints as indicated.

All ECG recordings must be performed using a standard high-quality, high-fidelity digital electrocardiograph machine equipped with computer-based interval measurements. Lead placement should be as consistent as possible. ECG recordings must be performed after the patient has been resting in a supine position for at least 10 minutes. All ECGs are to be obtained prior to other procedures scheduled at that same time (e.g., vital sign measurements, blood draws) and should not be obtained within 3 hours after any meal, if possible. Circumstances that may induce changes in heart rate, including environmental distractions (e.g., television, radio, conversation), should be avoided during the pre-ECG resting period and during ECG recording.

For safety monitoring purposes, the investigator must review, sign, and date all ECG tracings. Paper copies of ECG tracings will be kept as part of the patient's permanent study file at the site. Digital recordings will be stored at a central ECG laboratory. The following should be recorded in the appropriate eCRF: heart rate, RR interval, QRS interval, PR duration, uncorrected QT interval, and QTcF based on the machine readings of the individual ECG tracings. Any morphologic waveform changes or other ECG abnormalities must be documented on the eCRF. If considered appropriate by the Sponsor, ECGs may be analyzed retrospectively at a central laboratory.

If at a particular postdose timepoint the mean QTcF is > 500 ms and/or 60 ms longer than the baseline value, another triplicate ECG must be recorded, ideally within the next 5 minutes, and triplicate ECG monitoring should continue until QTcF has stabilized on two successive ECGs. The Medical Monitor should be notified. Standard-of-care treatment may be instituted per the discretion of the investigator. If a PK sample is not scheduled for that timepoint, an unscheduled PK sample should be obtained. A decision on study drug interruption or discontinuation should be made, as described in Section 5.1.7.8. The investigator should also evaluate the patient for potential concurrent risk factors (e.g., electrolyte abnormalities, co-medications known to prolong the QT interval, severe bradycardia).

[redacted]

[redacted]

[redacted]

[redacted] [redacted]

[redacted]

4.5.10 Blood Samples for Whole Genome Sequencing or Whole Exome Sequencing (Patients at Participating Sites)

At participating sites, blood samples will be collected for DNA extraction to enable whole genome sequencing (WGS) or whole exome sequencing (WES) to identify germline mutations and/or somatic mutations and human leukocyte antigen (HLA) type that are predictive of response to study drug, are associated with progression to a more severe disease state, are associated with acquired resistance to study drug, are associated with susceptibility to developing adverse events, or can increase the knowledge and understanding of disease biology or the safety profile of GDC-6036. DNA extracted from blood may be compared with DNA extracted from tissue to identify somatic variants by distinguishing germline variants from somatic variants. The blood samples may be sent to one or more laboratories for analysis.

Collection and submission of blood samples for WGS or WES is contingent upon the review and approval of the exploratory research by each site's IRB/EC and, if applicable, an appropriate regulatory body. If a site has not been granted approval for WGS or WES, this section of the protocol (Section 4.5.8) will not be applicable at that site.

Genomics is increasingly informing researcher's understanding of disease pathobiology. WGS and WES provide a comprehensive characterization of the genome and exome, respectively, and, along with clinical data collected in this study, may increase the opportunity for developing new therapeutic approaches or new methods for monitoring efficacy and safety or predicting which patients are more likely to respond to a drug or develop adverse events. Data will be analyzed in the context of this study but may also be explored in aggregate with data from other studies. The availability of a larger dataset will assist in identification and characterization of important biomarkers and pathways to support future drug development.

For sampling procedures, storage conditions, and shipment instructions, see the laboratory manual.

Blood samples collected for WGS or WES are to be stored until they are no longer needed or until they are exhausted. However, the storage period will be in accordance with the IRB/EC–approved Informed Consent Form and applicable laws (e.g., health authority requirements).

Patient medical information associated with WGS specimens is confidential and may be disclosed to third parties only as permitted by the Informed Consent Form (or separate authorization for use and disclosure of personal health information) signed by the patient, unless permitted or required by law.

Given the complexity and exploratory nature of the WGS analyses, data derived from these analyses will generally not be provided to study investigators or patients unless required by law. The aggregate results of any conducted research will be available in accordance with the effective Sponsor policy on study data publication.

Refer to Section 4.5.6 for details on use of samples after patient withdrawal, confidentiality standards for data, and availability of data from biomarker analyses.

4.5.11 *Pre-Treatment Tumor Tissue*

All patients will provide an archival or freshly collected (if archival is not available) tumor tissue sample obtained prior to treatment for determination of *KRAS G12C* mutation status and for exploratory research on biomarkers and biomarker assay development.

The *KRAS G12C* mutation assay will be performed by a local or central investigational test site. Patients may enroll based on available local test results for *KRAS G12C* mutation and must provide tumor tissue and blood sample for retrospective confirmation.

In addition, patients enrolled in Arm C, [redacted] who have previously been treated with a KRAS G12C inhibitor will provide an archival tumor tissue specimen collected prior to treatment with any KRAS G12C inhibitor (e.g., diagnostic specimen) and a recently acquired tumor specimen after completion of the last KRAS G12C inhibitor treatment.

Tumor tissue specimens should be from the most recent metastatic biopsy whenever possible (see laboratory manual for detailed instructions). A representative FFPE tumor specimen in a paraffin block (preferred) or at least 15 slides containing unstained, freshly cut, serial sections should be submitted along with an associated pathology report. If less than 15 slides are available, the patient may still enroll in the study, after discussion with the Medical Monitor.

Tumor tissue should be of good quality based on total and viable tumor content. Samples should contain a minimum of 500 viable tumor cells that preserve cellular context and tissue architecture regardless of needle gauge or retrieval method.

Samples collected via resection, core-needle biopsy (at least three cores, embedded in a single paraffin block), or excisional, incisional, punch, or forceps biopsy are acceptable. Fine-needle aspiration (defined as samples that do not preserve tissue architecture and yield cell suspension and/or smears), brushing, cell pellets from pleural effusion, and lavage samples are not allowed without prior approval from the Medical Monitor. Tumor tissue from bone metastases that have been decalcified is strongly discouraged.

If archival tumor tissue is unavailable or unsuitable, a pre-treatment tumor biopsy is required (see laboratory manual for detailed instructions). A fresh frozen tumor specimen should also be collected whenever possible.

[redacted]

Refer to Section 4.5.6 for details on duration of sample storage, use of samples for exploratory biomarker research and after patient withdrawal, confidentiality standards for data, and availability of data from biomarker analyses.

A detailed description of tissue quality requirements and sampling procedures for collection, storage conditions, handling, and shipping of tumor tissue samples will be provided in a separate laboratory manual.

4.5.12 *On-Treatment Tumor Biopsy ([redacted]. Optional for All Other Arms)*

[redacted]

Patients enrolled in all other arms may consent to provide an optional on-treatment tumor biopsy.

The Informed Consent Form will contain a separate section that addresses optional biopsies. A separate, specific signature will be required to document a patient's agreement to undergo optional biopsies. The investigator should document whether or not the patient has given consent to participate and (if applicable) the date(s) of consent, by completing the optional sample Informed Consent eCRF.

In lieu of a fresh pre-treatment biopsy, a recently obtained biopsy performed after completion of the last anti-cancer therapy will be acceptable.

On-treatment biopsies should be collected approximately 24 hours after taking study drug and before the next daily dosing, whenever possible, on Cycle 1, Days 10–16.

Samples collected via resection, core needle biopsy (at least three cores preferred), or excisional, incisional, punch, or forceps biopsy are preferred.

FFPE and fresh frozen samples should be prepared from newly collected tumor biopsies. At least two optimal cutting temperature (OCT) embedded (preferred) or snap-frozen

core needle biopsy and one FFPE core needle biopsy are requested for biomarker analysis. If an FFPE tumor block cannot be submitted for various reasons (e.g., due to site restrictions), approximately 15–20 paraffin-embedded, unstained slides from the on-treatment biopsy are requested.

[redacted]

Patients may consent to undergo additional on-treatment biopsies at any other time at the investigator's discretion (if deemed clinically feasible by the investigator) for exploratory research on biomarkers and biomarker assay development.

A detailed description of tissue quality requirements and procedures for collection, handling, and shipping of tumor tissue samples will be provided in a separate laboratory manual.

4.5.13 Optional Tumor *Biopsy* at the Time of Disease Progression

For patients who sign the Informed Consent Form for the optional biopsy at disease progression, a tumor biopsy will be collected upon disease progression. *Whenever* possible, the biopsy at disease progression should be collected within 24 hours *and up to 2 weeks after* the patient's last dose of GDC-6036.

FFPE *and fresh frozen* samples will be prepared from the tumor biopsy. *At least two optimal cutting temperature (OCT) embedded (preferred) or snap-frozen core needle biopsy and one FFPE core needle biopsy are requested for biomarker analysis. If an FFPE tumor block cannot be submitted for various reasons (e.g., due to site restrictions), approximately 15–20 paraffin-embedded, unstained slides from the on-treatment biopsy are requested.*

NGS may be performed on tissue submitted at the time of disease progression by FMI. If performed by FMI, the investigator may obtain an NGS report through Foundation Medicine's web portal. If allowed by local laws, the investigator may share and discuss the results with the patient, unless the patient chooses otherwise. The NGS report is generated for research purposes only and is not provided for the purpose of guiding future treatment decisions. Results may not be available for samples that do not meet criteria for testing or for samples that fail the testing process.

[redacted]

A detailed description of tissue quality requirements and procedures for collection, handling, and shipping of tumor tissue samples will be provided in a separate laboratory manual.

[redacted] [redacted]

[redacted]

[redacted]

[redacted]

4.5.15 Optional FDG-PET Imaging

FDG-PET imaging will be optional for all enrolled patients in both GDC-6036 single-agent and combination therapy arms. If a patient consents to this optional collection, scans should be acquired at baseline (up to 28 days prior to Cycle 1 Day 1) with repeat imaging between Days 10–16 of Cycle 1. If the baseline FDG-PET imaging for a patient shows no detectable tumor FDG uptake, no subsequent scan is needed. If at all possible, scans should be acquired prior to any biopsy procedures, if applicable. The timing and days of FDG-PET imaging may change based on PK and PD data acquired during the study. FDG-PET results should not be used for response assessment or for decisions regarding study treatment continuation or discontinuation.

FDG-PET imaging should extend at least from the orbital meatus to the proximal femurs, and scanner equipment should remain consistent throughout all scans for the same patient. Patients are required to fast for 4 hours prior to scanning; further instructions for patient preparation and technical details of the FDG-PET acquisition are described in the study imaging manual. FDG-PET scans will be collected for evaluation by a central independent review facility and/or by Genentech.

The Informed Consent Form will contain a separate section that addresses participation in the collection of FDG-PET scans. Patients must give consent prior to the FDG-PET scans being acquired. When a patient withdraws from the study, images collected prior to the date of withdrawal may still be analyzed. Imaging data will be subject to the confidentiality standards described in Section 8.4.

4.5.16 Collection of Photographs

Ad-hoc photographs of the skin, the mucous membranes, [redacted] may be taken and submitted to the Sponsor to allow further evaluation, *as allowed per regulatory authorities*. These photographs may be taken in the setting of toxicities of the skin, the mucous membranes, [redacted] (i.e., rash, mucositis, [redacted]) or in the event of lesions responding to study treatment.

The Informed Consent Form will contain a separate section that addresses participation in the collection of photographs. Patients must give consent prior to the photographs being taken.

The photographs should be taken in such a manner as not to create any risk of identifying the patient; photographs will be thoroughly de-identified at the sites prior to providing them to the Sponsor.

4.5.17 Optional Samples for Research Biosample Repository

4.5.17.1 Overview of the Research Biosample Repository

The Research Biosample Repository (RBR) is a centrally administered group of facilities used for the long-term storage of human biological specimens, including body fluids, solid tissues, and derivatives thereof (e.g., DNA, RNA, proteins, peptides). The collection, storage, and analysis of RBR samples will facilitate the rational design of new pharmaceutical agents and the development of diagnostic tests, which may allow for individualized drug therapy for patients in the future.

Samples for the RBR will be collected from patients who give specific consent to participate in this optional research. RBR samples will be analyzed to achieve one or more of the following objectives:

- To study the association of biomarkers with efficacy or disease progression
- To identify safety biomarkers that are associated with susceptibility to developing adverse events or can lead to improved adverse event monitoring or investigation
- To increase knowledge and understanding of disease biology and drug safety
- To study drug response, including drug effects and the processes of drug absorption and disposition
- To develop biomarker or diagnostic assays and establish the performance characteristics of these assays

4.5.17.2 Approval by the Institutional Review Board or Ethics Committee

Collection, storage, and analysis of RBR samples is contingent upon the review and approval of the exploratory research and the RBR portion of the Informed Consent Form by each site's *IRB/EC* and, if applicable, an appropriate regulatory body. If a site has not been granted approval for RBR sampling, this section of the protocol (Section 4.5.17) will not be applicable at that site.

4.5.17.3 Sample Collection

The following samples will be stored in the RBR and used for research purposes, including, but not limited to, research on biomarkers related to GDC-6036, diseases, or drug safety:

- Leftover blood, serum, plasma, urine, [redacted], frozen tumor tissue, *processed tumor samples,* and FFPE tissue samples (with the exception of remaining archival tissue blocks, which will be returned to sites) and any derivatives thereof (e.g., [redacted] *single cells,* DNA, RNA, proteins, peptides), including leftover tissue samples from medically indicated procedures (e.g., bronchoscopy, esophagogastroduodenoscopy, colonoscopy) performed at the investigator's discretion during the course of the study

The above samples may be sent to one or more laboratories for analysis of germline or somatic variants via whole genome sequencing (WGS), whole exome sequencing (WES), or other genomic analysis methods. Genomics is increasingly informing researcher's understanding of disease pathobiology. WGS and WES provide a comprehensive characterization of the genome and exome, respectively, and, along with clinical data collected in this study, may increase the opportunity for developing new therapeutic approaches or new methods for monitoring efficacy and safety or predicting which patients are more likely to respond to a drug or develop adverse events.

Data generated from RBR samples will be analyzed in the context of this study but may also be explored in aggregate with data from other studies. The availability of a larger dataset will assist in identification and characterization of important biomarkers and pathways to support future drug development.

For sampling procedures, storage conditions, and shipment instructions, see the laboratory manual.

RBR samples are to be stored until they are no longer needed or until they are exhausted. However, the RBR storage period will be in accordance with the IRB/EC–approved Informed Consent Form and applicable laws (e.g., health authority requirements).

4.5.17.4 Confidentiality

RBR samples and associated data will be labeled with a unique patient identification number.

Patient medical information associated with RBR samples is confidential and may be disclosed to third parties only as permitted by the Informed Consent Form (or separate authorization for use and disclosure of personal health information) signed by the patient, unless permitted or required by law.

Given the complexity and exploratory nature of the analyses of RBR samples, data derived from these analyses will generally not be provided to study investigators or patients unless required by law. The aggregate results of any conducted research will be available in accordance with the effective Sponsor policy on study data publication.

Data generated from RBR samples must be available for inspection upon request by representatives of national and local health authorities, and Sponsor monitors, representatives, and collaborators, as appropriate.

Any inventions and resulting patents, improvements, and/or know-how originating from the use of the RBR data will become and remain the exclusive and unburdened property of the Sponsor, except where agreed otherwise.

4.5.17.5 Consent to Participate in the Research Biosample Repository

The Informed Consent Form will contain a separate section that addresses participation in the RBR. The investigator or authorized designee will explain to each patient the objectives, methods, and potential hazards of participation in the RBR. Patients will be told that they are free to refuse to participate and may withdraw their consent at any time and for any reason during the storage period. A separate, specific signature will be required to document a patient's agreement to provide optional RBR samples. Patients who decline to participate will not provide a separate signature.

The investigator should document whether or not the patient has given consent to participate and (if applicable) the date(s) of consent, by completing the RBR Research Sample Informed Consent eCRF.

In the event of an RBR participant's death or loss of competence, the participant's samples and data will continue to be used as part of the RBR research.

4.5.17.6 Withdrawal from the Research Biosample Repository

Patients who give consent to provide RBR samples have the right to withdraw their consent at any time for any reason. After withdrawal of consent, any remaining samples will be destroyed or will no longer be linked to the patient. However, if RBR samples have been tested prior to withdrawal of consent, results from those tests will remain as part of the overall research data. If a patient wishes to withdraw consent to the testing of his or her RBR samples during the study, the investigator must inform the Medical Monitor in writing of the patient's wishes through use of the appropriate RBR Subject Withdrawal Form and must enter the date of withdrawal on the RBR Research Sample Withdrawal of Informed Consent eCRF. If a patient wishes to withdraw consent to the

testing of his or her RBR samples after closure of the site, the investigator must inform the Sponsor by emailing the study number and patient number to the following email address:

global_rcr-withdrawal@roche.com

A patient's withdrawal from this study does not, by itself, constitute withdrawal of consent for testing of RBR samples. Likewise, a patient's withdrawal of consent for testing of RBR samples does not constitute withdrawal from this study.

4.5.17.7 Monitoring and Oversight

RBR samples will be tracked in a manner consistent with Good Clinical Practice by a quality-controlled, auditable, and appropriately validated laboratory information management system, to ensure compliance with data confidentiality as well as adherence to authorized use of samples as specified in this protocol and in the Informed Consent Form. Sponsor monitors and auditors will have direct access to appropriate parts of records relating to patient participation in the RBR for the purposes of verifying the data provided to the Sponsor. The site will permit monitoring, audits, IRB/EC review, and health authority inspections by providing direct access to source data and documents related to the RBR samples.

4.6 TREATMENT, PATIENT, STUDY, AND SITE DISCONTINUATION

4.6.1 Study Treatment Discontinuation

Patients must permanently discontinue study treatment if they experience any of the following:

- Unequivocal disease progression (defined using RECIST v1.1; see Appendix 5) as determined by the investigator
- Symptomatic deterioration attributed to disease progression as determined by the investigator
- Intolerable toxicity related to study treatment determined by the investigator to be unacceptable given the potential for treatment benefit and the severity of the event
- Use of non-protocol systemic anti-cancer therapy (see Section 4.4.2)
- Any medical condition that the investigator or Sponsor determines may jeopardize the patient's safety if he or she continues to receive study treatment
- Investigator or Sponsor determination that treatment discontinuation is in the best interest of the patient
- Pregnancy

[redacted]

The primary reason for study treatment discontinuation should be documented on the appropriate eCRF. Patients who discontinue study treatment prematurely will not be replaced.

Patients who discontinue study drug will return to the clinic for a treatment discontinuation visit within 28 days after their final dose of study drug (see Appendix 1 through Appendix 3).

4.6.2 Patient Discontinuation from the Study

Patients have the right to voluntarily withdraw from the study at any time for any reason. In addition, the investigator has the right to withdraw a patient from the study at any time.

Reasons for patient discontinuation from the study may include, but are not limited to, the following:

- Patient withdrawal of consent
- Any medical condition that the investigator or Sponsor determines may jeopardize the patient's safety if he or she continues in the study
- Patient non-compliance, defined as failure to comply with protocol requirements as determined by the investigator or Sponsor
- Study termination or site closure

Every effort should be made to obtain a reason for patient discontinuation from the study. The primary reason for discontinuation from the study should be documented on the appropriate eCRF. Patients who withdraw from the study will not be replaced.

4.6.3 Study Discontinuation

The Sponsor has the right to terminate this study at any time. Reasons for terminating the study may include, but are not limited to, the following:

- The incidence or severity of adverse events in this or other studies indicates a potential health hazard to patients
- Patient enrollment is unsatisfactory
- Data recording is inaccurate or incomplete

The Sponsor will notify the investigator if the Sponsor decides to discontinue the study.

4.6.4 Site Discontinuation

The Sponsor has the right to close a site at any time. Reasons for closing a site may include, but are not limited to, the following:

- Excessively slow recruitment
- Poor protocol adherence
- Inaccurate or incomplete data recording

- Non-compliance with the International Council for Harmonisation (ICH) guideline for Good Clinical Practice
- No study activity (i.e., all patients have completed the study and all obligations have been fulfilled)

5. ASSESSMENT OF SAFETY

5.1 SAFETY PLAN

GDC-6036 is not approved, and clinical development is ongoing; thus, the safety profile of the study medication and its combinations are not known at this time. The safety plan for patients in this study is based on the anticipated mechanism of action, nonclinical data (in vitro and in vivo), published data on similar KRAS G12C molecules, and the individual clinical safety profiles of the combination medications established to date. The anticipated important safety risks for GDC-6036 and the combination study medications are outlined below. Please refer to the GDC-6036, [redacted] Investigator's Brochures and the cetuximab [redacted] U.S. Package Inserts and E.U. SmPCs for complete summaries of individual drug safety information.

[redacted]

Several measures will be taken to ensure the safety of patients participating in this study. Eligibility criteria have been designed to exclude patients at higher risk for toxicities. Patients will undergo safety monitoring during the study, including assessment of the nature, frequency, and severity of adverse events graded according to NCI CTCAE v5.0. All serious adverse events, potential DLTs, and adverse events of special interest will be reported in "real time" (within 24 hours of the event) via the electronic data capture (EDC) system (for more details, see Section 5.2). In addition, adverse events will be discussed at regularly scheduled teleconferences between the Medical Monitor and the investigators.

In the setting of a pandemic or epidemic, screening for active infections (including SARS-CoV-2) prior to and during study participation should be performed according to local or institutional guidelines or guidelines of applicable professional societies (e.g., American Society of Clinical Oncology or European Society for Medical Oncology).

Safety assessments will include interval history since the previous assessment, physical examinations, and specific laboratory studies, including CBCs and a chemistry panel. Patients will be monitored as outlined in Appendix 1 and Appendix 2 until the end of the adverse event reporting period. In addition, guidelines for managing adverse events,

including criteria for dose modification and treatment interruption or discontinuation, are provided below.

As with any first-in-human study, there is the potential for toxicities that cannot be predicted from animal studies. To mitigate potential unknown risks, at least in part, dosing beyond Cycle 1 will be limited to patients who are tolerating GDC-6036 treatment and not exhibiting signs or symptoms of disease progression.

Dose escalation to the next highest level will occur following a review of available safety data after the last patient completes Cycle 1 in each cohort.

5.1.1 Risks Associated with GDC-6036

5.1.1.1 Potential Gastrointestinal Toxicities (Diarrhea, Nausea, and Vomiting)

In 4-week toxicology studies in cynomolgus monkeys with GDC-6036, clinical signs of gastrointestinal intolerance with retching and vomiting were observed. *Diarrhea, nausea, and vomiting have been reported in patients treated with GDC-6036. Please refer to the GDC-6036 Investigator's Brochure for additional information.*

Gastrointestinal toxicities (e.g., diarrhea, nausea, vomiting) have been reported in association with the oral KRAS G12C inhibitors AMG 510 (sotorasib) and MRTX849 currently in early clinical development. All grade adverse events assessed AMG 510 *(all events)* or MRTX849 *(related only)* included diarrhea (29.5% and 70%), nausea (20.9% and 60%), and vomiting (17.8% and 29%), respectively (AMG 510, n=129; MRTX849, n=17) (Janne et al. 2019; Hong et al. 2020). [redacted]

The mechanism of action *underlying GI toxicities reported with oral KRAS G12C inhibitors remains* unknown. *Patients* should be closely monitored for signs and symptoms related to gastrointestinal effects.

Development of abdominal pain, nausea, vomiting, and clinically significant changes in stool (e.g., diarrhea, bloody stools) may necessitate more frequent monitoring, and study drug may be held if symptoms are prohibitive for normal function. Clinical evaluation for infectious (e.g., *Clostridium difficile*, enteric bacteria, and cytomegalovirus) or inflammatory (e.g., inflammatory colitis) etiologies for diarrhea should be conducted with additional consideration for gastrointestinal consultation as needed. *Patients should be made aware of the risk for diarrhea, nausea, and vomiting and should be instructed to inform their treating physician as soon as possible if they experience these events. Patients* should be treated and managed per standard of care and per protocol guidelines (Section 5.1.7.2), including *early* use of anti-emetics, anti-diarrheals*, probiotics*, and appropriate supportive care including hydration and dietary modification if clinically indicated.

5.1.1.2 Potential Oral Mucosal Irritation

In preclinical studies with mice, microscopic evidence of laryngeal necrosis and inflammation was observed. The laryngeal findings were attributable to incidental exposure to the higher concentration drug formulation during the gavage process leading to chemical irritation. Refer to the GDC-6036 Investigator's Brochure, Section 4.3 for a detailed description of the nonclinical toxicology findings.

Laryngitis, pharyngitis, esophagitis, and mucositis have not been reported for the oral KRAS G12C inhibitors currently in early clinical development.

Patients should be instructed on proper pill swallowing without chewing or crushing the capsule or tablet. A minimum of 240 mL (8 ounces) of water should be taken with GDC-6036. Patients should be monitored for signs of stomatitis or mucositis. Refer to Section 5.1.7.2 for specific guidelines.

5.1.1.3 Potential Elevation of Hepatic Transaminases

In cynomolgus monkeys, minimal to mild transaminase elevation has been observed after administration of GDC-6036. Hepatic transaminase elevation has been reported for the oral KRAS G12C inhibitors currently in early clinical development. In the Phase I trial with AMG 510 (sotorasib), 17 out of 129 (13.2%) patients had reported any grade increased AST and 15 out of 129 (11.6%) patients had reported any grade increased ALT (Hong et al. 2020). Grade 3 treatment-related increased AST and ALT was reported in 3 out of 129 (2.3%) and 6 out of 129 (4.7%) patients respectively. One patient (0.8%) reported a Grade 4 treatment-related elevation of ALT, which returned to the baseline level after dose reduction and glucocorticosteroid tapering. One patient (0.8%) discontinued treatment because of Grade 3 treatment-related increase in ALT and AST levels. In the ongoing Phase I trial with MRTX849, 5 out of 17 patients had reported Grade 1 increased AST and 3 out 17 patients had reported Grade 1 increased ALT (Janne et al. 2019).

Patients will be monitored routinely with chemistry panels that include liver function tests and for signs or symptoms of hepatic pathology (e.g., jaundice, coagulopathy, abdominal pain). Patients with underlying liver disease will be excluded from this study. Patients experiencing hepatic enzyme elevation should be treated and managed per standard of care and protocol guidelines (see Section 5.1.7.2).

5.1.1.4 Potential Drug-Drug Interactions

GDC-6036 has been shown to have the potential to induce messenger RNA (mRNA), but not protein levels of CYP3A4 in human hepatocyte culture. Therefore, interaction with co-administered CYP3A4 substrates cannot be excluded at this point. As such, care should be taken with co-administered drugs that are metabolized by these enzyme systems, especially if those drugs have a narrow therapeutic index, because their systemic concentrations could be altered when co-administered with GDC-6036. There is also a low potential for drug-drug interaction with co-administered CYP3A4 modulators

but potential drug-drug interaction cannot be ruled out. Thus, caution should be taken with any medication that is metabolized by or strongly inhibits or induces this enzyme (see Section 4.4.2.1).

GDC-6036 has been shown to inhibit P-gp and BCRP transporters in vitro. While exposure of prototypical substrates of P-gp and BCRP transporters is not predicted to be affected based on PBPK-modeling based simulations in the clinical dose range of GDC-6036, interaction with co-administered P-gp, and BCRP substrates cannot be excluded. Caution should be taken with any medication that is an exclusive substrate of P-gp or BCRP with a narrow therapeutic index (see Section 4.4.2.1).

[redacted]

[redacted]

[redacted]

[redacted]

[redacted]

[redacted]

[redacted]

[redacted]

[redacted]

[redacted]

[redacted]

5.1.3 Risks Associated with Cetuximab

A comprehensive description of the safety profile of cetuximab is provided in the U.S. Package Insert and the E.U. SmPC. The summary information provided in the following section must always be checked against any update that may have occurred after the protocol has been issued.

The main undesirable effects of cetuximab are skin reactions which occur in more than 80% of patients (see Appendix 9 for management guidelines), hypomagnesaemia which occurs in more than 10% of patients, and IRRs which occur with mild to moderate symptoms in more than 10% of patients and with severe symptoms in more than 1% of patients. The risk of serious infusion reactions with cetuximab administration may be increased in patients who have had a tick bite or have a red meat allergy. Cardiopulmonary arrest and/or sudden death has been reported in 2% of patients receiving cetuximab in combination with radiation therapy (Bonner et al. 2006). A case of tumor lysis syndrome has been reported with single-agent cetuximab administration

within 24 hours of the first dose (Krishnan et al. 2008). More details are provided in the locally approved cetuximab label (see local prescribing information for cetuximab [Erbitux U.S. Package Insert; Erbitux E.U. SmPC; or equivalent country specific document]).

Potential overlapping toxicities associated with the combination use of cetuximab and GDC-6036 are gastrointestinal toxicities and elevated hepatic transaminases.

[redacted]

[redacted]

[redacted]

[redacted]

[redacted]

[redacted]

[redacted]

[redacted]

[redacted]

[redacted]

[redacted]

[redacted]

[redacted]

[redacted]

[redacted]

[redacted]

[redacted]

[redacted]

[redacted]

[redacted]

[redacted]

[redacted]

[redacted]

[redacted]

[redacted]

[redacted]

[redacted]

[redacted]

[redacted] [redacted]

[redacted]

[redacted]

[redacted]

[redacted] [redacted]

[redacted]

[redacted]

[redacted]

[redacted]

5.1.7 Management of Patients Who Experience Adverse Events

[redacted] These guidelines are intended to inform rather than supersede clinical judgment and the benefit–risk balance as assessed by the investigator in managing individual cases. In general, for the management of specific adverse events of Grade ≥3 outlined below, GDC-6036 administration should be interrupted.

[redacted]

[redacted]

For combination IMPs, specific adverse events requiring management and their management regimens are provided in Appendix 8 through *Appendix 12* and in the reference safety information.

For specific adverse events for all IMPs, the following general rules should apply:

- Investigate etiology [redacted]
- Initiate treatment as per institutional guidelines and check the relevant Investigator's Brochure(s)/SmPC for specific management guidelines.
- Contact the Medical Monitor as appropriate and as per Investigator's Brochure guidance.

5.1.7.1 Dose Modifications and Treatment Interruption

There are no dose reductions built into the study design for GDC-6036 during the DLT evaluation period. Patients who reduce the GDC-6036 dose during the DLT assessment window for reasons other than DLT may be replaced.

Patients who experience a DLT during the DLT assessment window (see Section 3.1.1.3) should have GDC-6036 dosing suspended. GDC-6036 should be permanently discontinued, except when the DLT is reversible and monitorable and the potential benefit–risk ratio suggests a reasonable rationale for the patient to continue study treatment, with approval by the Medical Monitor. In such cases, if the DLT improves to Grade ≤1 within 21 days, GDC-6036 dosing may resume and must be at one dose level lower (defined as the dose level of the prior cohort). If dose reduction below the dose level of the first cohort in Stage I, Arm A is required, the dose will be reduced from 50 mg to 25 mg. *For combination partners, refer to the safety management guidelines (see Appendix 8 through Appendix 12).*

If a patient in Stage I (dose-escalation stage) or Stage II (dose-expansion stage) has unacceptable GDC-6036–related toxicity per the investigator's clinical judgment, adjustment of the study treatment may be warranted, including but not requiring dose reduction, in consultation with the Medical Monitor. If GDC-6036 dosing is held for the toxicity, and upon adequate recovery the investigator determines that the patient should continue to receive the study treatment, GDC-6036 may be re-initiated upon agreement from the Medical Monitor. No dose re-escalation will be allowed without Medical Monitor approval.

If, in the opinion of the investigator, more than one dose level reduction is required, a second dose level reduction may be considered with approval of the Medical Monitor. No more than two dose-level reductions for a single patient will be allowed.

If GDC-6036 is held for >21 days from the previous study treatment due to toxicity, the study treatment should not be re-initiated without agreement from the Medical Monitor.

GDC-6036 may be suspended for up to 21 days for unanticipated intercurrent medical events that are not associated with study treatment toxicity or disease progression. If, in the judgment of the investigator, the patient is likely to derive clinical benefit from resuming GDC-6036 after dosing has been suspended for more than 21 days, study drug may be re-initiated with prior approval of the Medical Monitor.

If cumulative safety data suggest that a dose level initially selected for expansion is not tolerable, accrual at that dose level will be halted after consultation with the trial investigators (see Section 3.1.1.4). Individual patients who continue on study treatment will have the option of GDC-6036 dose reduction if, in the opinion of the investigator, that would be in that patient's best interest.

For dose modifications and treatment interruptions with combination study drugs, please refer to Appendix 8 through Appendix 12 and to the relevant reference safety information for the individual study drug.

5.1.7.2 Management Guidelines

Guidelines for management of specific adverse events with GDC-6036 are outlined in Table 3.

[redacted]

Table 3 Guidelines for Management of Patients Who Experience Adverse Events with GDC-6036

Event	Action to Be Taken
**Gastrointestinal Toxicities (Nausea, Vomiting, Diarrhea)**	
General	• Infectious or alternate etiology should be excluded.<br>• Patients should be closely monitored for GI symptoms and effects on well-being. Patients experiencing nausea, vomiting, and/or diarrhea should be managed according to local standards of care, including use of anti-emetics, anti-diarrheal agents, and appropriate supportive care including hydration and dietary modification as appropriate.<br>• Do not administer anti-diarrheals until at least 4 hours postdose.
Grade 1–2	• Manage with supportive care according to local standard of care. If persistent despite maximum medical therapy, hold treatment with GDC-6036 until resolution to Grade ≤ 1.
Grade ≥ 3	• Manage with supportive care according to local standard<br>• Hold treatment with GDC-6036 until resolution to Grade ≤ 1.<br>• Consider GI consult if inadequate response to supportive care and GDC-6036 interruption.<br>• Reduce GDC-6036 dose by one dose level [a] when treatment resumed if toxicity attributed to GDC-6036.<br>• Discuss with Medical Monitor for appropriate dose modification and management in patients receiving combination treatment when causality cannot be adequately determined.<br>• If Grade 4, permanently discontinue GDC-6036.
**Stomatitis/Mucosal Inflammation**	
General	• For any grade stomatitis/mucosal inflammation, aggressive mouth care that includes mouthwash formulations (e.g., combinations of local anesthetics, anti-histamine, corticosteroid, antacid, antifungal and/or antibiotics) may be implemented to help manage symptoms. Diet should be modified (e.g., avoidance of spicy food) and harsh mouth washes (e.g., Listerine) should be avoided.
Grade 1	• Monitor symptoms and initiate management (see above). Re-evaluate within 48–72 hours.
Grade 2	• Interrupt GDC-6036 and manage until Grade ≤ 1.<br>• When stomatitis/oral mucositis improves to Grade ≤ 1, resume dosing at the same dose or one dose level lower per investigator evaluation.<br>• For recurrent Grade 2 stomatitis or oral mucositis within 30 days, resume dosing at one dose level lower.
Grade ≥ 3	• Interrupt GDC-6036 and manage until Grade ≤ 1.<br>− When stomatitis/oral mucositis improves to Grade ≤ 1, resume dosing at one dose level lower if toxicity attributed to GDC-6036. Discuss with Medical Monitor for appropriate dose modification and management in patients receiving combination treatment when causality cannot be adequately determined.<br>• If Grade 4, permanently discontinue GDC-6036.

Table 3 Guidelines for Management of Patients Who Experience Adverse Events with GDC-6036 (cont.)

**Elevation of Hepatic Transaminases**	
General	• Patients presenting with jaundice, coagulopathy, abdominal pain or other symptoms suggestive of hepatic pathology should have their liver function tests checked and imaging of the liver performed. If the liver enzymes are elevated with no obvious malignant cause found, a hepatologist should be consulted.<br>• Patients experiencing hepatic enzyme elevation should be treated and managed per standard of care.<br>• Please refer to management sections for hepatic events attributed to individual combination agents (Appendix 8 through Appendix 12). If individual component causality cannot be adequately determined, the most conservative management recommendation should be applied. Consider early consultation with a hepatologist and the Medical Monitor for appropriate dose modification and management.
Grade ≥3	• Hold GDC-6036 until resolution to baseline or below upper limit of normal for AST/ALT or total bilirubin.<br>• Discuss with Medical Monitor for appropriate dose modification and management *in patients receiving combination treatment when causality cannot be adequately determined.*<br>• If Grade 4 or any case meeting Hy's Law criteria, permanently discontinue GDC-6036.

GI = gastrointestinal.

[a] Defined as the dose level of the previous cohort.

[redacted]

5.1.7.4 Management of Patients Who Experience Specific Adverse Events with Cetuximab

Guidelines for managing anticipated adverse events with cetuximab, including criteria for treatment interruption or discontinuation and dosage modification, are provided in Appendix 9.

[redacted]

[redacted]

[redacted]

5.1.7.8 Management of Increases in QT Interval

Study drug should be discontinued in patients who develop any of the following, unless there is a clear alternative cause for the changes:

- Sustained (at least two ECG measurements > 30 minutes apart) QTcF that is > 500 ms and/or > 60 ms longer than the baseline value
- Sustained absolute QTcF that is > 515 ms
- An episode of torsades de pointes or a new ECG finding of clinical concern

Of note, if there is a new intraventricular conduction block, the increase in QRS complex duration should be subtracted from the QTcF change, because this represents an increase in QTcF unrelated to alterations in repolarization. Also of note, it is not uncommon to record arrhythmias such as non-sustained ventricular tachycardia, supraventricular tachycardia, pauses, or atrial fibrillation in healthy volunteers receiving placebo during periods of extended ECG monitoring. Therefore, it is critical that expert cardiology advice be sought to confirm any ECG changes and to ascertain the likelihood of a drug-induced arrhythmia versus the background occurrence of this arrhythmia. In such a situation, saving all available ECG data is highly suggested.

Management of patients with sustained QTcF prolongation should include close monitoring, with ECGs repeated at least hourly until two successive ECGs show resolution of the findings, correction of any electrolyte abnormalities, and possible discontinuation of other concomitant medications that are known to prolong the QT interval. Consultation with a cardiologist or electrophysiologist is recommended, to help in the management of such patients. The Medical Monitor should be notified as soon as possible.

In rare circumstances, it may be acceptable to resume study drug, at a lower dose, provided that any ECG abnormalities have resolved and the patient is appropriately monitored. Clinical judgment should be applied and with agreement from the Medical Monitor.

5.2 SAFETY PARAMETERS AND DEFINITIONS

Safety assessments will consist of monitoring and recording adverse events, including serious adverse events and adverse events of special interest, performing protocol-specified safety laboratory assessments, measuring protocol-specified vital signs, and conducting other protocol-specified tests that are deemed critical to the safety evaluation of the study.

Certain types of events require immediate reporting to the Sponsor, as outlined in Section 5.4.

5.2.1 Adverse Events

According to the ICH guideline for Good Clinical Practice, an adverse event is any untoward medical occurrence in a clinical investigation subject administered a pharmaceutical product, regardless of causal attribution. An adverse event can therefore be any of the following:

- Any unfavorable and unintended sign (including an abnormal laboratory finding), symptom, or disease temporally associated with the use of a medicinal product, whether or not considered related to the medicinal product
- Any new disease or exacerbation of an existing disease (a worsening in the character, frequency, or severity of a known condition) (see Sections 5.3.5.9 and 5.3.5.10 for more information)
- Recurrence of an intermittent medical condition (e.g., headache) not present at baseline
- Any deterioration in a laboratory value or other clinical test (e.g., ECG, X-ray) that is associated with symptoms or leads to a change in study treatment or concomitant treatment or discontinuation from study drug
- Adverse events that are related to a protocol-mandated intervention, including those that occur prior to assignment of study treatment (e.g., screening invasive procedures such as biopsies)

5.2.2 Serious Adverse Events (Immediately Reportable to the Sponsor)

A serious adverse event is any adverse event that meets any of the following criteria:

- Is fatal (i.e., the adverse event actually causes or leads to death)
- Is life threatening (i.e., the adverse event, in the view of the investigator, places the patient at immediate risk of death)

    This does not include any adverse event that, had it occurred in a more severe form or was allowed to continue, might have caused death.

- Requires or prolongs inpatient hospitalization (see Section 5.3.5.10)
- Results in persistent or significant disability/incapacity (i.e., the adverse event results in substantial disruption of the patient's ability to conduct normal life functions)
- Is a congenital anomaly/birth defect in a neonate/infant born to a mother exposed to study drug
- Is a significant medical event in the investigator's judgment (e.g., may jeopardize the patient or may require medical/surgical intervention to prevent one of the outcomes listed above)

The terms "severe" and "serious" are not synonymous. Severity refers to the intensity of an adverse event (e.g., rated as mild, moderate, or severe, or according to NCI CTCAE; see Section 5.3.3); the event itself may be of relatively minor medical significance (such as severe headache without any further findings).

Severity and seriousness need to be independently assessed for each adverse event recorded on the eCRF.

Serious adverse events are required to be reported by the investigator to the Sponsor immediately (i.e., no more than 24 hours after learning of the event; see Section 5.4.2 for reporting instructions).

5.2.3 Adverse Events of Special Interest (Immediately Reportable to the Sponsor)

Adverse events of special interest are required to be reported by the investigator to the Sponsor immediately (i.e., no more than 24 hours after learning of the event; see Section 5.4.2 for reporting instructions).

5.2.3.1 Adverse Events of Special Interest for GDC-6036 (All Arms)

Adverse events of special interest for GDC-6036 are as follows:

- DLTs occurring during the DLT assessment window
- Grade $\geq$3 nausea/vomiting/diarrhea
- Grade $\geq$3 stomatitis/mucositis
- Grade $\geq$3 hepatitis or elevation in ALT or AST
- Cases of potential drug-induced liver injury that include an elevated ALT or AST in combination with either an elevated bilirubin or clinical jaundice, as defined by Hy's Law (see Section 5.3.5.6)

- Suspected transmission of an infectious agent by the study drug, as defined below

  Any organism, virus, or infectious particle (e.g., prion protein transmitting transmissible spongiform encephalopathy), pathogenic or non-pathogenic, is considered an infectious agent. A transmission of an infectious agent may be suspected from clinical symptoms or laboratory findings that indicate an infection in a patient exposed to a medicinal product. This term applies <u>only</u> when a contamination of the study drug is suspected.

[redacted]

- [redacted]
- [redacted]
- [redacted]
- [redacted]
- [redacted]
- [redacted]
- [redacted]
- [redacted]
- [redacted]

Cetuximab (Arm C)

There are no adverse events of special interest specific to cetuximab.

[redacted]

[redacted]

[redacted]

- [redacted]
- [redacted]
- [redacted]
- [redacted]

5.2.4 Dose-Limiting Toxicities (Immediately Reportable to the Sponsor)

During the DLT assessment window, adverse events identified as DLTs, as defined in Section 3.1.1.3, are required to be reported by the investigator to the Sponsor immediately (i.e., no more than 24 hours after learning of the event; see Section 5.4.2 for reporting instructions).

5.3 METHODS AND TIMING FOR CAPTURING AND ASSESSING SAFETY PARAMETERS

The investigator is responsible for ensuring that all adverse events (see Section 5.2.1 for definition) are recorded on the Adverse Event eCRF and reported to the Sponsor in accordance with instructions provided in this section and in Sections 5.4–5.6.

For each adverse event recorded on the Adverse Event eCRF, the investigator will make an assessment of seriousness (see Section 5.2.2 for seriousness criteria), severity (see Section 5.3.3), and causality (see Section 5.3.4).

5.3.1 Adverse Event Reporting Period

Investigators will seek information on adverse events at each patient contact. All adverse events, whether reported by the patient or noted by study personnel, will be recorded in the patient's medical record and on the Adverse Event eCRF.

After informed consent has been obtained but prior to initiation of study drug(s), only serious adverse events caused by a protocol-mandated intervention (e.g., invasive procedures such as biopsies, discontinuation of medications) should be reported (see Section 5.4.2 for instructions for reporting serious adverse events).

After initiation of study drug(s), all adverse events will be reported for the following durations after the final dose of study drug or until they receive another anti-cancer therapy, whichever occurs first:

- Arms [redacted] C, [redacted] 28 days after the final dose of any study drug
- [redacted]

[redacted]

Instructions for reporting adverse events that occur after the adverse event reporting period are provided in Section 5.6.

5.3.2 Eliciting Adverse Event Information

A consistent methodology of non-directive questioning should be adopted for eliciting adverse event information at all patient evaluation timepoints. Examples of nondirective questions include the following:

"How have you felt since your last clinic visit?"

"Have you had any new or changed health problems since you were last here?"

5.3.3 Assessment of Severity of Adverse Events

The adverse event severity grading scale for the NCI CTCAE (v5.0) will be used for assessing adverse event severity. Table 4 will be used for assessing severity for adverse events that are not specifically listed in the NCI CTCAE.

Table 4 Adverse Event Severity Grading Scale for Events Not Specifically Listed in NCI CTCAE

Grade	Severity
1	Mild; asymptomatic or mild symptoms; clinical or diagnostic observations only; or intervention not indicated
2	Moderate; minimal, local, or non-invasive intervention indicated; or limiting age-appropriate instrumental activities of daily living [a]
3	Severe or medically significant, but not immediately life-threatening; hospitalization or prolongation of hospitalization indicated; disabling; or limiting self-care activities of daily living [b, c]
4	Life-threatening consequences or urgent intervention indicated [d]
5	Death related to adverse event [d]

NCI CTCAE = National Cancer Institute Common Terminology Criteria for Adverse Events.

Note: Based on the most recent version of NCI CTCAE (v5.0), which can be found at: http://ctep.cancer.gov/protocolDevelopment/electronic_applications/ctc.htm

[a] Instrumental activities of daily living refer to preparing meals, shopping for groceries or clothes, using the telephone, managing money, etc.

[b] Examples of self-care activities of daily living include bathing, dressing and undressing, feeding oneself, using the toilet, and taking medications, as performed by patients who are not bedridden.

[c] If an event is assessed as a "significant medical event," it must be reported as a serious adverse event (see Section 5.4.2 for reporting instructions), per the definition of serious adverse event in Section 5.2.2.

[d] Grade 4 and 5 events must be reported as serious adverse events (see Section 5.4.2 for reporting instructions), per the definition of serious adverse event in Section 5.2.2.

5.3.4 Assessment of Causality of Adverse Events

Investigators should use their knowledge of the patient, the circumstances surrounding the event, and an evaluation of any potential alternative causes to determine whether an adverse event is considered to be related to the study drug, indicating "yes" or "no" accordingly. The following guidance should be taken into consideration (see also Table 5):

- Temporal relationship of event onset to the initiation of study drug
- Course of the event, with special consideration of the effects of dose reduction, discontinuation of study drug, or reintroduction of study drug (as applicable)
- Known association of the event with the study drug or with similar treatments
- Known association of the event with the disease under study
- Presence of risk factors in the patient or use of concomitant medications known to increase the occurrence of the event
- Presence of non-treatment-related factors that are known to be associated with the occurrence of the event

Table 5 Causal Attribution Guidance

Is the adverse event suspected to be caused by the study drug on the basis of facts, evidence, science-based rationales, and clinical judgment?	
YES	There is a plausible temporal relationship between the onset of the adverse event and administration of the study drug, and the adverse event cannot be readily explained by the patient's clinical state, intercurrent illness, or concomitant therapies; and/or the adverse event follows a known pattern of response to the study drug; and/or the adverse event abates or resolves upon discontinuation of the study drug or dose reduction and, if applicable, reappears upon *re-challenge*.
NO	An adverse event will be considered related, unless it fulfills the criteria specified below. Evidence exists that the adverse event has an etiology other than the study drug (e.g., preexisting medical condition, underlying disease, intercurrent illness, or concomitant medication); and/or the adverse event has no plausible temporal relationship to administration of the study drug (e.g., cancer diagnosed 2 days after first dose of study drug).

For patients receiving combination therapy, causality will be assessed individually for each protocol-mandated therapy.

5.3.5 Procedures for Recording Adverse Events

Investigators should use correct medical terminology/concepts when recording adverse events on the Adverse Event eCRF. Avoid colloquialisms and abbreviations.

Only one adverse event term should be recorded in the event field on the Adverse Event eCRF.

5.3.5.1 Diagnosis versus Signs and Symptoms

A diagnosis (if known) should be recorded on the Adverse Event eCRF rather than individual signs and symptoms (e.g., record only liver failure or hepatitis rather than jaundice, asterixis, and elevated transaminases). However, if a constellation of signs and/or symptoms cannot be medically characterized as a single diagnosis or syndrome at the time of reporting, each individual event should be recorded on the Adverse Event eCRF. If a diagnosis is subsequently established, all previously reported adverse events based on signs and symptoms should be nullified and replaced by one adverse event report based on the single diagnosis, with a starting date that corresponds to the starting date of the first symptom of the eventual diagnosis.

5.3.5.2 Adverse Events That Are Secondary to Other Events

In general, adverse events that are secondary to other events (e.g., cascade events or clinical sequelae) should be identified by their primary cause, with the exception of severe or serious secondary events. A medically significant secondary adverse event that is separated in time from the initiating event should be recorded as an independent event on the Adverse Event eCRF. For example:

- If vomiting results in mild dehydration with no additional treatment in a healthy adult, only vomiting should be reported on the eCRF.
- If vomiting results in severe dehydration, both events should be reported separately on the eCRF.
- If a severe gastrointestinal hemorrhage leads to renal failure, both events should be reported separately on the eCRF.
- If dizziness leads to a fall and consequent fracture, all three events should be reported separately on the eCRF.
- If neutropenia is accompanied by an infection, both events should be reported separately on the eCRF.

All adverse events should be recorded separately on the Adverse Event eCRF if it is unclear as to whether the events are associated.

5.3.5.3 Persistent or Recurrent Adverse Events

A persistent adverse event is one that extends continuously, without resolution, between patient evaluation timepoints. Such events should only be recorded once on the Adverse Event eCRF. The initial severity (intensity or grade) of the event will be recorded at the time the event is first reported. If a persistent adverse event becomes more severe, the most extreme severity should also be recorded on the Adverse Event eCRF. Details regarding any increases or decreases in severity will be captured on the Adverse Event Intensity or Grade Changes eCRF. If the event becomes serious, it should be reported to the Sponsor immediately (i.e., no more than 24 hours after learning that the event became serious; see Section 5.4.2 for reporting instructions). The Adverse Event eCRF should be updated by changing the event from "non-serious"

to "serious," providing the date that the event became serious, and completing all data fields related to serious adverse events.

A recurrent adverse event is one that resolves between patient evaluation timepoints and subsequently recurs. Each recurrence of an adverse event should be recorded as a separate event on the Adverse Event eCRF.

5.3.5.4 Abnormal Laboratory Values

Not every laboratory abnormality qualifies as an adverse event. A laboratory test result must be reported as an adverse event if it meets any of the following criteria:

- Is accompanied by clinical symptoms
- Results in a change in study treatment (e.g., dosage modification, treatment interruption, or treatment discontinuation)
- Results in a medical intervention (e.g., potassium supplementation for hypokalemia) or a change in concomitant therapy
- Is clinically significant in the investigator's judgment

    Note: For oncology trials, certain abnormal values may not qualify as adverse events.

It is the investigator's responsibility to review all laboratory findings. Medical and scientific judgment should be exercised in deciding whether an isolated laboratory abnormality should be classified as an adverse event.

If a clinically significant laboratory abnormality is a sign of a disease or syndrome (e.g., alkaline phosphatase and bilirubin $5 \times$ ULN associated with cholestasis), only the diagnosis (i.e., cholestasis) should be recorded on the Adverse Event eCRF.

If a clinically significant laboratory abnormality is not a sign of a disease or syndrome, the abnormality itself should be recorded on the Adverse Event eCRF, along with a descriptor indicating whether the test result is above or below the normal range (e.g., "elevated potassium," as opposed to "abnormal potassium"). If the laboratory abnormality can be characterized by a precise clinical term per standard definitions, the clinical term should be recorded as the adverse event. For example, an elevated serum potassium level of 7.0 mEq/L should be recorded as "hyperkalemia."

Observations of the same clinically significant laboratory abnormality from visit to visit should only be recorded once on the Adverse Event eCRF (see Section 5.3.5.4 for details on recording persistent adverse events).

5.3.5.5 Abnormal Vital Sign Values

Not every vital sign abnormality qualifies as an adverse event. A vital sign result must be reported as an adverse event if it meets any of the following criteria:

- Is accompanied by clinical symptoms

- Results in a change in study treatment (e.g., dosage modification, treatment interruption, or treatment discontinuation)
- Results in a medical intervention or a change in concomitant therapy
- Is clinically significant in the investigator's judgment

It is the investigator's responsibility to review all vital sign findings. Medical and scientific judgment should be exercised in deciding whether an isolated vital sign abnormality should be classified as an adverse event.

If a clinically significant vital sign abnormality is a sign of a disease or syndrome (e.g., high blood pressure), only the diagnosis (i.e., hypertension) should be recorded on the Adverse Event eCRF.

Observations of the same clinically significant vital sign abnormality from visit to visit should only be recorded once on the Adverse Event eCRF (see Section 5.3.5.4 for details on recording persistent adverse events).

5.3.5.6 Abnormal Liver Function Tests

The finding of an elevated ALT or AST ($>3\times$ baseline value) in combination with either an elevated total bilirubin ($>2\times$ ULN) or clinical jaundice in the absence of cholestasis or other causes of hyperbilirubinemia is considered to be an indicator of severe liver injury (as defined by Hy's Law). Therefore, investigators must report as an adverse event the occurrence of either of the following:

- Treatment-emergent ALT or AST $>3\times$ baseline value in combination with total bilirubin $>2\times$ ULN (of which $\geq$35% is direct bilirubin)
- Treatment-emergent ALT or AST $>3\times$ baseline value in combination with clinical jaundice

The most appropriate diagnosis or (if a diagnosis cannot be established) the abnormal laboratory values should be recorded on the Adverse Event eCRF (see Section 5.3.5.1) and reported to the Sponsor immediately (i.e., no more than 24 hours after learning of the event), either as a serious adverse event or an adverse event of special interest (see Section 5.4.2).

5.3.5.7 Deaths

All deaths that occur during the protocol-specified adverse event reporting period (see Section 5.3.1), regardless of relationship to study drug, must be recorded on the Adverse Event eCRF and immediately reported to the Sponsor (see Section 5.4.2). This includes death attributed to progression of *KRAS G12C*–positive cancer.

Death should be considered an outcome and not a distinct event. The event or condition that caused or contributed to the fatal outcome should be recorded as the single medical concept on the Adverse Event eCRF. Generally, only one such event should be reported. If the cause of death is unknown and cannot be ascertained at the time of reporting, **"unexplained death"** should be recorded on the Adverse Event eCRF. If the

cause of death later becomes available (e.g., after autopsy), "unexplained death" should be replaced by the established cause of death. The term **"sudden death"** should not be used unless combined with the presumed cause of death (e.g., "sudden cardiac death").

If the death is attributed solely to progression of *KRAS G12C*–positive cancer, the "identified cancer progression" (e.g., NSCLC, CRC, or other type of cancer progression) should be recorded on the Adverse Event eCRF.

Deaths that occur after the adverse event reporting period should be reported as described in Section 5.6.

5.3.5.8 Preexisting Medical Conditions

A preexisting medical condition is one that is present at the screening visit for this study. Such conditions should be recorded on the General Medical History and Baseline Conditions eCRF.

A preexisting medical condition should be recorded as an adverse event only if the frequency, severity, or character of the condition worsens during the study. When recording such events on the Adverse Event eCRF, it is important to convey the concept that the preexisting condition has changed by including applicable descriptors (e.g., "more frequent headaches").

5.3.5.9 Lack of Efficacy or Worsening of *KRAS G12C*–Positive Cancer

Events that are clearly consistent with the expected pattern of progression of the underlying disease should not be recorded as adverse events. These data will be captured as efficacy assessment data only. In most cases, the expected pattern of progression will be based on RECIST. In rare cases, the determination of clinical progression will be based on symptomatic deterioration. However, every effort should be made to document progression through use of objective criteria. If there is any uncertainty as to whether an event is due to disease progression, it should be reported as an adverse event.

5.3.5.10 Hospitalization or Prolonged Hospitalization

Any adverse event that results in hospitalization (i.e., inpatient admission to a hospital) or prolonged hospitalization should be documented and reported as a serious adverse event (per the definition of serious adverse event in Section 5.2.2), except as outlined below.

An event that leads to hospitalization under the following circumstances should not be reported as an adverse event or a serious adverse event:

- Hospitalization for respite care
- Planned hospitalization required by the protocol (e.g., for study drug administration or insertion of access device for study drug administration)

- Hospitalization for a preexisting condition, provided that all of the following criteria are met:

  The hospitalization was planned prior to the study or was scheduled during the study when elective surgery became necessary because of the expected normal progression of the disease

  The patient has not experienced an adverse event
- Hospitalization due solely to progression of the underlying cancer

An event that leads to hospitalization under the following circumstances is not considered to be a serious adverse event, but should be reported as an adverse event instead:

- Hospitalization that was necessary because of patient requirement for outpatient care outside of normal outpatient clinic operating hours

5.3.5.11 Cases of Overdose, Medication Error, Drug Abuse, or Drug Misuse

Overdose (accidental or intentional), medication error, drug abuse, and drug misuse (hereafter collectively referred to as "special situations"), are defined as follows:

- Accidental overdose: accidental administration of a drug in a quantity that is higher than the assigned dose
- Intentional overdose: intentional administration of a drug in a quantity that is higher than the assigned dose
- Medication error: accidental deviation in the administration of a drug (e.g., wrong dose administered, expired drug administered)

  In some cases, a medication error may be intercepted prior to administration of the drug.
- Drug abuse: intentional excessive use of a drug that may lead to addiction or dependence, physical harm, and/or psychological harm
- Drug misuse: intentional deviation in the administration of a drug that does not qualify as drug abuse

  In cases where drug is to be self-administered by the patient, drug misuse could involve the drug being administered to someone other than the patient.

Special situations are not in themselves adverse events, but may result in adverse events. Each adverse event associated with a special situation should be recorded separately on the Adverse Event eCRF. If the associated adverse event fulfills seriousness criteria *or qualifies as an adverse event of special interest*, the event should be reported to the Sponsor immediately (i.e., no more than 24 hours after learning of the event; see Section 5.4.2). For GDC-6036, adverse events associated with special situations should be recorded as described below for each situation:

- Accidental overdose: Enter the adverse event term. Check the "Accidental overdose" and "Medication error" boxes.

- Intentional overdose: Enter the adverse event term. Check the "Intentional overdose" box. If drug abuse is suspected, check the "Drug abuse" box. If drug abuse is not suspected, check the "Drug misuse" box.
- Medication error that does not qualify as an overdose: Enter the adverse event term. Check the "Medication error" box.
- Medication error that qualifies as an overdose: Enter the adverse event term. Check the "Accidental overdose" and "Medication error" boxes.
- Drug abuse that does not qualify as an overdose: Enter the adverse event term. Check the "Drug abuse" box.
- Drug abuse that qualifies as an overdose: Enter the adverse event term. Check the "Intentional overdose" and "Drug abuse" boxes.
- Drug misuse that does not qualify as an overdose: Enter the adverse event term. Check the "Drug misuse" box.
- Drug misuse that qualifies as an overdose: Enter the adverse event term. Check the "Intentional overdose" and "Drug misuse" boxes.

In addition, all special situations associated with GDC-6036, regardless of whether they result in an adverse event, should be recorded on the eCRF as described below:

- Accidental overdose: Enter the drug name and "accidental overdose" as the event term. Check the "Accidental overdose" and "Medication error" boxes.
- Intentional overdose: Enter the drug name and "intentional overdose" as the event term. Check the "Intentional overdose" box. If drug abuse is suspected, check the "Drug abuse" box. If drug abuse is not suspected, check the "Drug misuse" box.
- Medication error that does not qualify as an overdose: Enter the name of the drug administered and a description of the error (e.g., wrong dose administered incorrect route of administration, wrong drug, expired drug administered) as the event term. Check the "Medication error" box.
- Medication error that qualifies as an overdose: Enter the drug name and "accidental overdose" as the event term. Check the "Accidental overdose" and "Medication error" boxes. Enter a description of the error in the additional case details.
- Intercepted medication error: Enter the drug name and "intercepted medication error" as the event term. Check the "Medication error" box. Enter a description of the error in the additional case details.
- Drug abuse that does not qualify as an overdose: Enter the drug name and "drug abuse" as the event term. Check the "Drug abuse" box.
- Drug abuse that qualifies as an overdose: Enter the drug name and "intentional overdose" as the event term. Check the "Intentional overdose" and "Drug abuse" boxes.

- Drug misuse that does not qualify as an overdose: Enter the drug name and "drug misuse" as the event term. Check the "Drug misuse" box.
- Drug misuse that qualifies as an overdose: Enter the drug name and "intentional overdose" as the event term. Check the "Intentional overdose" and "Drug misuse" boxes.
- Drug administered to someone other than the patient: Enter the drug name and "patient supplied drug to third party" as the event term. Check the "Drug misuse" box.

As an example, an accidental overdose that resulted in a headache would require two entries on the Adverse Event eCRF, one entry to report the accidental overdose and one entry to report the headache. The "Accidental overdose" and "Medication error" boxes would need to be checked for both entries.

5.3.5.12 Safety Biomarker Data

Adverse event reports will not be derived from exploratory safety biomarker data by the Sponsor, and exploratory safety biomarker data will not be included in the formal safety analyses for this study. In addition, safety biomarker data will not inform decisions on patient management.

5.4 IMMEDIATE REPORTING REQUIREMENTS FROM INVESTIGATOR TO SPONSOR

Certain events require immediate reporting to allow the Sponsor to take appropriate measures to address potential new risks in a clinical trial. The investigator must report such events to the Sponsor immediately; under no circumstances should reporting take place more than 24 hours after the investigator learns of the event. The following is a list of events that the investigator must report to the Sponsor within 24 hours after learning of the event, regardless of relationship to study drug:

- Serious adverse events (defined in Section 5.2.2; see Section 5.4.2 for details on reporting requirements)
- Adverse events of special interest (defined in Section 5.2.3; see Section 5.4.2 for details on reporting requirements)
- DLTs during the DLT assessment window (defined in Section 5.2.4; see Section 5.4.2 for details on reporting requirements)
- Pregnancies (see Section 5.4.3 for details on reporting requirements)

For serious adverse events and adverse events of special interest, the investigator must report new significant follow-up information to the Sponsor immediately (i.e., no more than 24 hours after becoming aware of the information). New significant information includes the following:

- New signs or symptoms or a change in the diagnosis
- Significant new diagnostic test results

- Change in causality based on new information
- Change in the event's outcome, including recovery
- Additional narrative information on the clinical course of the event

Investigators must also comply with local requirements for reporting serious adverse events to the local health authority and IRB/EC.

5.4.1 Emergency Medical Contacts

Medical Monitor Contact Information

Genentech Medical Monitor contact information:

Medical Monitor: [redacted]

Telephone Nos.: [redacted]

Alternate Medical Monitor contact information for all sites:

Telephone Nos.: [redacted]

5.4.2 Reporting Requirements for Serious Adverse Events, Adverse Events of Special Interest, and Dose-Limiting Toxicities

5.4.2.1 Events That Occur prior to Study Drug Initiation

After informed consent has been obtained but prior to initiation of study drug, only serious adverse events caused by a protocol-mandated intervention should be reported. The paper Clinical Trial Serious Adverse Event/Adverse Event of Special Interest Reporting Form provided to investigators should be completed and submitted to the Sponsor or its designee immediately (i.e., no more than 24 hours after learning of the event), either by faxing or by scanning and emailing the form using the fax number or email address provided to investigators.

5.4.2.2 Events That Occur after Study Drug Initiation

After initiation of study drug, serious adverse events and adverse events of special interest will be reported for a treatment-specific period after the final dose of study drug or until they receive another anti-cancer therapy, whichever occurs first (see Section 5.3.1). DLTs will be reported during the DLT assessment window. Investigators should record all case details that can be gathered immediately (i.e., within 24 hours after learning of the event) on the Adverse Event eCRF and submit the report via the electronic data capture (EDC) system. A report will be generated and sent to Safety Risk Management by the EDC system.

In the event that the EDC system is unavailable, the paper Clinical Trial Serious Adverse Event/Adverse Event of Special Interest Reporting Form provided to investigators should be completed and submitted to the Sponsor or its designee immediately (i.e., no more

than 24 hours after learning of the event), either by faxing or by scanning and emailing the form using the fax number or email address provided to investigators. Once the EDC system is available, all information will need to be entered and submitted via the EDC system.

Instructions for reporting serious adverse events that occur after the treatment-specific period after the final dose of study treatment or until they receive another anti-cancer therapy, whichever occurs first (see Section 5.3.1), are provided in Section 5.6.

5.4.3 Reporting Requirements for Pregnancies

5.4.3.1 Pregnancies in Female Patients

Female patients of childbearing potential will be instructed *through the Informed Consent Form* to immediately inform the investigator if they become pregnant during the study or within 6 months after the final dose of study drug. A paper Clinical Trial Pregnancy Reporting Form should be completed and submitted to the Sponsor or its designee immediately (i.e., no more than 24 hours after learning of the pregnancy), either by faxing or by scanning and emailing the form using the fax number or email address provided to investigators. Pregnancy should not be recorded on the Adverse Event eCRF. The investigator should discontinue study drug and counsel the patient, discussing the risks of the pregnancy and the possible effects on the fetus. Monitoring of the patient should continue until conclusion of the pregnancy. Any serious adverse events associated with the pregnancy (e.g., an event in the fetus, an event in the mother during or after the pregnancy, or a congenital anomaly/birth defect in the child) should be reported on the Adverse Event eCRF. In addition, the investigator will submit a Clinical Trial Pregnancy Reporting Form when updated information on the course and outcome of the pregnancy becomes available.

5.4.3.2 Pregnancies in Female Partners of Male Patients

Male patients will be instructed through the Informed Consent Form to immediately inform the investigator if their partner becomes pregnant during the study or within 4 months after the final dose of study drug. *The investigator should report the pregnancy on the* paper Clinical Trial Pregnancy Reporting Form and *submit the form to* the Sponsor or its designee immediately (i.e., no more than 24 hours after learning of the pregnancy), either by faxing or by scanning and emailing the form using the fax number or email address provided to investigators. Attempts should be made to collect and report details of the course and outcome of any pregnancy in the partner of a male patient exposed to study drug. When permitted by the site, the pregnant partner would need to sign an Authorization for Use and Disclosure of Pregnancy Health Information to allow for follow-up on her pregnancy. If the authorization has been signed, the investigator should submit a Clinical Trial Pregnancy Reporting Form *with additional* information on the *pregnant partner and the* course and outcome of the pregnancy *as it* becomes available. An investigator who is contacted by the male patient or his pregnant partner may provide information on the risks of the pregnancy and the possible effects

on the fetus, to support an informed decision in cooperation with the treating physician and/or obstetrician.

5.4.3.3 Abortions

A spontaneous abortion should be classified as a serious adverse event (as the Sponsor considers abortions to be medically significant), recorded on the Adverse Event eCRF, and reported to the Sponsor immediately (i.e., no more than 24 hours after learning of the event; see Section 5.4.2).

If a therapeutic or elective abortion was performed because of an underlying maternal or embryofetal toxicity, the toxicity should be classified as a serious adverse event, recorded on the Adverse Event eCRF, and reported to the Sponsor immediately (i.e., no more than 24 hours after learning of the event; see Section 5.4.2). A therapeutic or elective abortion performed for reasons other than an underlying maternal or embryofetal toxicity is not considered an adverse event.

All abortions should be reported as pregnancy outcomes on the paper Clinical Trial Pregnancy Reporting Form.

5.4.3.4 Congenital Anomalies/Birth Defects

Any congenital anomaly/birth defect in a child born to a female patient exposed to study drug or the female partner of a male patient exposed to study drug should be classified as a serious adverse event, recorded on the Adverse Event eCRF, and reported to the Sponsor immediately (i.e., no more than 24 hours after learning of the event; see Section 5.4.2).

5.5 FOLLOW-UP OF PATIENTS AFTER ADVERSE EVENTS

5.5.1 Investigator Follow-Up

The investigator should follow each adverse event until the event has resolved to baseline grade or better, the event is assessed as stable by the investigator, the patient is lost to follow-up, or the patient withdraws consent. Every effort should be made to follow all serious adverse events considered to be related to study drug or trial-related procedures until a final outcome can be reported.

During the study period, resolution of adverse events (with dates) should be documented on the Adverse Event eCRF and in the patient's medical record to facilitate source data verification.

All pregnancies reported during the study should be followed until pregnancy outcome.

5.5.2 Sponsor Follow-Up

For serious adverse events, adverse events of special interest, and pregnancies, the Sponsor or a designee may follow up by telephone, fax, email, and/or a monitoring visit to obtain additional case details and outcome information (e.g., from hospital discharge

summaries, consultant reports, autopsy reports) in order to perform an independent medical assessment of the reported case.

5.6 ADVERSE EVENTS THAT OCCUR AFTER THE ADVERSE EVENT REPORTING PERIOD

The Sponsor should be notified if the investigator becomes aware of any serious adverse event that occurs after the end of the adverse event reporting period (defined as a treatment-specific period after the final dose of study drug or until they receive another anti-cancer therapy, whichever occurs first [see Section 5.3.1]), if the event is believed to be related to prior study drug treatment. These events should be reported through use of the Adverse Event eCRF. However, if the EDC system is not available, the investigator should report these events directly to the Sponsor or its designee, either by faxing or by scanning and emailing the paper Clinical Trial Serious Adverse Event/Adverse Event of Special Interest Reporting Form using the fax number or email address provided to investigators.

5.7 EXPEDITED REPORTING TO HEALTH AUTHORITIES, INVESTIGATORS, INSTITUTIONAL REVIEW BOARDS, AND ETHICS COMMITTEES

The Sponsor will promptly evaluate all serious adverse events and adverse events of special interest against cumulative product experience to identify and expeditiously communicate possible new safety findings to investigators, IRBs, ECs, and applicable health authorities based on applicable legislation.

To determine reporting requirements for single adverse event cases, the Sponsor will assess the expectedness of these events through use of the reference safety information (RSI) in the documents listed below:

Drug	Document
GDC-6036	GDC-6036 Investigator's Brochure
[redacted]	[redacted]
Cetuximab	Cetuximab $E.U.$ SmPC
[redacted]	[redacted]
[redacted]	[redacted]
[redacted]	[redacted]

The Sponsor will compare the severity of each event and the cumulative event frequency reported for the study with the severity and frequency reported in the applicable reference document.

Reporting requirements will also be based on the investigator's assessment of causality and seriousness, with allowance for upgrading by the Sponsor as needed.

6. STATISTICAL CONSIDERATIONS AND ANALYSIS PLAN

Listings will be used in place of tables for small numbers of patients. All analyses will be based on the safety-evaluable population, defined as all patients who receive any amount of study drug.

Continuous variables will be summarized using means, standard deviations, median, and ranges; categorical variables will be presented using counts and percentages. All summaries will be calculated by dose and by cohort in all safety-evaluable patients.

6.1 DETERMINATION OF SAMPLE SIZE

This study is intended to obtain preliminary safety, PK, and activity information in the safety-evaluable population. The sample sizes do not reflect any explicit power and type I error considerations.

6.1.1 Dose Escalation (Stage I)

The sample size for this trial is based on the dose-escalation rules described in Section 3.1.1.4. The planned enrollment is [redacted] patients for the combination dose-escalation stage (Arms [redacted] C, [redacted]).

[redacted] Stage I dose-escalation arms in combination with other anti-cancer therapies will follow a 3+3 design based on the criteria described in Section 3.1.1.2. Table 6 provides probabilities of observing no DLT in 3 patients or observing ≤1 DLT in 6 patients, given different underlying DLT rates.

Table 6 Probability of Observing DLTs with Different Underlying DLT Rates

True Underlying DLT Rate	Probability of Observing no DLT in 3 Patients	Probability of Observing ≤1 DLT in 6 Patients
0.10	0.73	0.89
0.20	0.51	0.66
0.33	0.30	0.36
0.40	0.22	0.23
0.50	0.13	0.11
0.60	0.06	0.04

6.1.2 Dose Expansion (Stage II)

The planned enrollment for the expansion stage of this study [redacted] Arm C, [redacted]) is up to approximately [redacted] patients.

Approximately 20 patients each will be enrolled in [redacted] CRC, [redacted] cohorts to assess the safety, tolerability, and preliminary evidence of anti-tumor activity of GDC-6036 administered at or below its MTD or MAD as a single agent or in combination with other anti-cancer therapies. Table 7 provides probabilities of observing at least one adverse event among 20 patients for probabilities ranging from 0.01 to 0.2 (i.e., adverse event frequencies of 1%–20%) *and illustrates that the planned sample size is sufficient to provide a meaningful likelihood of observing adverse events occurring at appreciable frequency (5% or higher). For a given adverse event with a true rate of 20%, 10%, or 5%, the probability of observing at least one adverse event in an expanded cohort of 20 patients is 0.99, 0.88, and 0.64, respectively.*

Table 7 Probability of Safety-Signal Detection with an Expansion Cohort of 20 Patients

True Underlying Probability of an Adverse Event	Probability of Observing ≥ 1 Adverse Event in 20 Patients
0.01	0.18
0.05	0.64
0.10	0.88
0.15	0.96
0.20	0.99

All available safety data will be evaluated to assess the tolerability of the treatment for a specific indication. Table 8 shows the two-sided Clopper-Pearson exact confidence interval for up to four observed adverse events.

Table 8 Two-Sided Exact Confidence Intervals for Observed Adverse Events in 20 Patients

Number of Observed Adverse Events	*Point Estimate (80% CI)*
0	*0% (0%–10.9%)*
1	*5.0% (0.5%–18.1%)*
2	*10.0% (2.7%–24.5%)*
3	*15.0% (5.6%–30.4%)*
4	*20.0% (9.0%–36.1%)*

CI = confidence interval.

In addition, the cohort size of 20 patients will enable a preliminary assessment of anti-tumor activity. The observed ORR will be used to evaluate the potential benefit

for patients. Table 9 shows the two-sided Clopper-Pearson exact confidence intervals for the observed ORR of 10%–50%.

Table 9 Two-Sided Exact Confidence Intervals for Observed Responses in 20 Patients

Number of Observed Responses	*Point Estimate (80% CI)*
2	10.0% (2.7%–24.5%)
3	15.0% (5.6%–30.4%)
4	20.0% (9.0%–36.1%)
5	25.0% (12.7%–41.5%)
6	30.0% (16.6%–46.7%)
7	35.0% (20.7%–51.8%)
8	40.0% (24.9%–56.7%)
9	45.0% (29.3%–61.5%)
10	50.0% (33.8%–66.2%)

CI = confidence interval.

6.2 SUMMARIES OF CONDUCT OF STUDY

The number of patients who enroll, discontinue, or complete the study will be summarized. Reasons for premature study discontinuation will be listed and summarized. Enrollment and major protocol deviations will be listed and evaluated for their potential effects on the interpretation of study results.

6.3 SUMMARIES OF DEMOGRAPHIC AND BASELINE CHARACTERISTICS

Demographic and baseline characteristics (including age, sex, and race/ethnicity) will be summarized using means, standard deviations, medians, and ranges for continuous variables and proportions for categorical variables, as appropriate. Summaries will be presented overall and by dose level and cancer type.

6.4 SAFETY ANALYSES

The safety analysis population will consist of all patients who received at least one dose of study drug, with patients grouped according to treatment received.

Safety will be assessed through summaries of exposure to study treatment, adverse events, changes in laboratory test results, and changes in vital signs and ECGs.

Study treatment exposure (such as treatment duration, total dose received, and number of cycles and dose modifications) will be summarized with descriptive statistics.

All verbatim adverse event terms will be mapped to Medical Dictionary for Regulatory Activities (MedDRA) thesaurus terms, and adverse event severity will be graded

according to NCI CTCAE v5.0. All adverse events, serious adverse events, adverse events leading to death, adverse events of special interest, and adverse events leading to study treatment discontinuation that occur on or after the first dose of study treatment (i.e., treatment emergent adverse events) will be summarized by mapped term, appropriate thesaurus level, and severity grade. For events of varying severity, the highest grade will be used in the summaries. Deaths and cause of death will be summarized.

Relevant laboratory, vital sign (pulse rate, respiratory rate, blood pressure, pulse oximetry, and temperature; oxygen saturation at screening), and ECG data will be displayed by time, with grades identified where appropriate. Additionally, a shift table of selected laboratory tests will be used to summarize the baseline and maximum postbaseline severity grade. Changes in vital signs and ECGs will be summarized.

6.5 PHARMACOKINETIC ANALYSES

The following primary PK parameters will be derived from the plasma concentration–time profile of GDC-6036 (all arms), [redacted] following administration of single and/or multiple doses, when appropriate as data allow:

- AUC_{inf} after single dose and $AUC_{0-\tau}$ after single and multiple doses
- Maximum plasma concentration (C_{max})
- Minimum plasma concentration (C_{min})
- Time to maximum plasma concentration (t_{max})
- Apparent terminal half-life ($t_{1/2}$)
- Apparent clearance (CL/F) after single and multiple doses
- Accumulation ratio (AR) at steady-state

The following secondary PK parameters may also be derived, when appropriate data allow:

- PK-dose proportionality as assessed with C_{max} and AUC
- The time to achieve steady state as assessed with trough (predose) concentrations

Additional PK analyses will be conducted as appropriate. The C_{max} and C_{min} will be determined for [redacted] cetuximab (Arm C), [redacted]

[redacted]

6.7 ACTIVITY ANALYSES

Objective response, DOR, and PFS will be summarized for safety evaluable patients by dose level*, cohort and cancer type.*

Response assessment *data will be reported as the proportion of responders with Clopper-Pearson exact binomial confidence intervals. Median DOR and median PFS will be estimated using Kaplan-Meier methodology. The 95% confidence interval of median DOR and median PFS will be estimated using Brookmeyer and Crowley method. If median DOR or median PFS cannot be estimated, the 12-month DOR rate or the 12-month PFS rate will be estimated. The 95% confidence interval of the DOR rate and the PFS rate will be estimated using the standard error derived from Greenwood's formula.*

6.7.1 *Objective Response Rate*

Response assessment will be summarized as complete response (CR), partial response (PR), stable disease (SD), non-CR/non-PD, and progressive disease (PD). Objective response is defined as a CR or PR as determined by investigator assessment per RECIST v1.1. Patients with missing or no response assessments will be classified as non-responders. ORR will be estimated for all patients and by cohort and cancer type. Both confirmed response and unconfirmed response will be summarized.

6.7.2 *Duration of Response*

Among patients with an objective response, DOR will be defined as the time from the initial CR or PR to the time of disease progression or death. If a patient does not experience disease progression or death before the end of the study, DOR will be censored at the day of the last tumor assessment.

6.7.3 *Progression-Free Survival*

The analyses of PFS will include patients who have received any amount of study treatment. PFS is defined as the time from first treatment at Day 1 of Cycle 1 to the first occurrence of disease progression or death from any cause during the study

(whichever occurs first), as determined by the investigator according to RECIST v1.1. If a patient does not experience disease progression or death before the end of the study, PFS will be censored at the day of the last tumor assessment.

6.8 BIOMARKER ANALYSES

Although no formal statistical analysis of exploratory biomarkers will be performed, data may be analyzed in the context of this study and in aggregate with data from other studies.

Exploratory biomarker analyses may be performed in an effort to understand the association of these markers with study treatment response. The biomarker analysis will be descriptive and exploratory in nature. Trends and variability will be summarized with confidence intervals. Results will be presented in a separate report.

The exploratory biomarker outcome measures for this study are as follows:

- Alkylation of KRAS G12C by GDC-6036
- Assessment of ctDNA biomarkers (e.g., *KRAS G12C*) from peripheral blood
- Alterations in DNA, RNA, and protein, including DNA mutation status and copy number; RNA expression levels, localization and splicing; and protein expression (e.g., PD-L1)
- Modulation of KRAS/MAPK target genes (e.g., *DUSP6*, *SPRY4*), *RTK/MAPK.* pathway components (e.g., pERK, pS6 [redacted]), and related biomarkers (e.g., Ki67) through analysis of paired pre-treatment and on-treatment fresh tumor biopsies
- Biomarker profiling of progression tumor biopsies to identify mechanisms of resistance to *the KRAS G12C inhibitor (e.g.,* GDC-6036*)*
- Relationship between biomarkers in blood and tumor tissue (including germline and somatic mutations) and safety, PK, activity, or other biomarker endpoints

7. DATA COLLECTION AND MANAGEMENT

7.1 DATA QUALITY ASSURANCE

The Sponsor will be responsible for data management of this study, including quality checking of the data. Data entered manually will be collected via EDC through use of eCRFs. Sites will be responsible for data entry into the EDC system. In the event of discrepant data, the Sponsor will request data clarification from the sites, which the sites will resolve electronically in the EDC system.

The Sponsor will produce an EDC Study Specification document that describes the quality checking to be performed on the data. Central laboratory data, pharmacokinetic, biomarker, and imaging results will be sent directly to the Sponsor, using the Sponsor's standard procedures to handle and process the electronic transfer of these data.

eCRFs and correction documentation will be maintained in the EDC system's audit trail. System backups for data stored by the Sponsor and records retention for the study data will be consistent with the Sponsor's standard procedures.

7.2 ELECTRONIC CASE REPORT FORMS

eCRFs are to be completed through use of a Sponsor-designated EDC system. Sites will receive training and have access to a manual for appropriate eCRF completion. eCRFs will be submitted electronically to the Sponsor and should be handled in accordance with instructions from the Sponsor.

All eCRFs should be completed by designated, trained site staff. eCRFs should be reviewed and electronically signed and dated by the investigator or a designee.

At the end of the study, the investigator will receive patient data for his or her site in a readable format that must be kept with the study records. Acknowledgement of receipt of the data is required.

7.3 SOURCE DATA DOCUMENTATION

Study monitors will perform ongoing source data verification and review to confirm that critical protocol data (i.e., source data) entered into the eCRFs by authorized site personnel are accurate, complete, and verifiable from source documents.

Source documents (paper or electronic) are those in which patient data are recorded and documented for the first time. They include, but are not limited to, hospital records, clinical and office charts, laboratory notes, memoranda, patient-reported outcomes, evaluation checklists, pharmacy dispensing records, recorded data from automated instruments, copies of transcriptions that are certified after verification as being accurate and complete, microfiche, photographic negatives, microfilm or magnetic media, X-rays, patient files, and records kept at pharmacies, laboratories, and medico-technical departments involved in a clinical trial.

Before study initiation, the types of source documents that are to be generated will be clearly defined in the Trial Monitoring Plan. This includes any protocol data to be entered directly into the eCRFs (i.e., no prior written or electronic record of the data) and considered source data.

Source documents that are required to verify the validity and completeness of data entered into the eCRFs must not be obliterated or destroyed and must be retained per the policy for retention of records described in Section 7.5.

To facilitate source data verification and review, the investigators and institutions must provide the Sponsor direct access to applicable source documents and reports for trial-related monitoring, Sponsor audits, and IRB/EC review. The study site must also allow inspection by applicable health authorities.

7.4 USE OF COMPUTERIZED SYSTEMS

When clinical observations are entered directly into a study site's computerized medical record system (i.e., in lieu of original hardcopy records), the electronic record can serve as the source document if the system has been validated in accordance with health authority requirements pertaining to computerized systems used in clinical research. An acceptable computerized data collection system allows preservation of the original entry of data. If original data are modified, the system should maintain a viewable audit trail that shows the original data as well as the reason for the change, name of the person making the change, and date of the change.

7.5 RETENTION OF RECORDS

Records and documents pertaining to the conduct of this study and the distribution of IMP, including eCRFs, electronic or paper PRO data (if applicable), Informed Consent Forms, laboratory test results, and medication inventory records, must be retained by the Principal Investigator for 15 years after completion or discontinuation of the study or for the length of time required by relevant national or local health authorities, whichever is longer. After that period of time, the documents may be destroyed, subject to local regulations.

No records may be disposed of without the written approval of the Sponsor. Written notification should be provided to the Sponsor prior to transferring any records to another party or moving them to another location.

Roche will retain study data for 25 years after the final study results have been reported or for the length of time required by relevant national or local health authorities, whichever is longer.

8. ETHICAL CONSIDERATIONS

8.1 COMPLIANCE WITH LAWS AND REGULATIONS

This study will be conducted in full conformance with the ICH E6 guideline for Good Clinical Practice and the principles of the Declaration of Helsinki, or the applicable laws and regulations of the country in which the research is conducted, whichever affords the greater protection to the individual. The study will comply with the requirements of the ICH E2A guideline (Clinical Safety Data Management: Definitions and Standards for Expedited Reporting). Studies conducted in the United States or under a U.S. Investigational New Drug (IND) Application will comply with U.S. FDA regulations and applicable local, state, and federal laws. Studies conducted in the European Union or European Economic Area will comply with the E.U. Clinical Trial Directive (2001/20/EC) and applicable local, regional, and national laws.

8.2 INFORMED CONSENT

The Sponsor's sample Informed Consent Form (and ancillary sample Informed Consent Forms such as an Assent Form or Mobile Nursing Informed Consent Form, if applicable)

will be provided to each site. If applicable, it will be provided in a certified translation of the local language. The Sponsor or its designee must review and approve any proposed deviations from the Sponsor's sample Informed Consent Forms or any alternate consent forms proposed by the site (collectively, the "Consent Forms") before IRB/EC submission. The final IRB/EC–approved Consent Forms must be provided to the Sponsor for health authority submission purposes according to local requirements.

If applicable, the Informed Consent Form will contain separate sections for any optional procedures. The investigator or authorized designee will explain to each patient the objectives, methods, and potential risks associated with each optional procedure. Patients will be told that they are free to refuse to participate and may withdraw their consent at any time for any reason. A separate, specific signature will be required to document a patient's agreement to participate in optional procedures. Patients who decline to participate will not provide a separate signature.

The Consent Forms must be signed and dated by the patient or the patient's legally authorized representative before his or her participation in the study. The case history or clinical records for each patient shall document the informed consent process and that written informed consent was obtained prior to participation in the study.

The Consent Forms should be revised whenever there are changes to study procedures or when new information becomes available that may affect the willingness of the patient to participate. The final revised IRB/EC-approved Consent Forms must be provided to the Sponsor for health authority submission purposes.

If the Consent Forms are revised (through an amendment or an addendum) while a patient is participating in the study, the patient or a legally authorized representative must re-consent by signing the most current version of the Consent Forms or the addendum, in accordance with applicable laws and IRB/EC policy. For any updated or revised Consent Forms, the case history or clinical records for each patient shall document the informed consent process and that written informed consent was obtained using the updated/revised Consent Forms for continued participation in the study.

A copy of each signed Consent Form must be provided to the patient or the patient's legally authorized representative. All signed and dated Consent Forms must remain in each patient's study file or in the site file and must be available for verification by study monitors at any time.

For sites in the United States, each Consent Form may also include patient authorization to allow use and disclosure of personal health information in compliance with the U.S. Health Insurance Portability and Accountability Act (HIPAA) of 1996. If the site utilizes a separate Authorization Form for patient authorization for use and disclosure of personal health information under the HIPAA regulations, the review, approval, and other

processes outlined above apply except that IRB review and approval may not be required per study site policies.

8.3 INSTITUTIONAL REVIEW BOARD OR ETHICS COMMITTEE

This protocol, the Informed Consent Forms, any information to be given to the patient, and relevant supporting information must be submitted to the IRB/EC by the Principal Investigator and reviewed and approved by the IRB/EC before the study is initiated. In addition, any patient recruitment materials must be approved by the IRB/EC.

The Principal Investigator is responsible for providing written summaries of the status of the study to the IRB/EC annually or more frequently in accordance with the requirements, policies, and procedures established by the IRB/EC. Investigators are also responsible for promptly informing the IRB/EC of any protocol amendments (see Section 9.6).

In addition to the requirements for reporting all adverse events to the Sponsor, investigators must comply with requirements for reporting serious adverse events to the local health authority and IRB/EC. Investigators may receive written IND safety reports or other safety-related communications from the Sponsor. Investigators are responsible for ensuring that such reports are reviewed and processed in accordance with health authority requirements and the policies and procedures established by their IRB/EC, and archived in the site's study file.

8.4 CONFIDENTIALITY

The Sponsor maintains confidentiality standards by coding each patient enrolled in the study through assignment of a unique patient identification number. This means that patient names are not included in data sets that are transmitted to any Sponsor location.

Patient medical information obtained by this study is confidential and may be disclosed to third parties only as permitted by the Informed Consent Form (or separate authorization for use and disclosure of personal health information) signed by the patient, unless permitted or required by law.

Medical information may be given to a patient's personal physician or other appropriate medical personnel responsible for the patient's welfare, for treatment purposes.

Given the complexity and exploratory nature of exploratory biomarker analyses, data derived from these analyses will generally not be provided to study investigators or patients unless required by law (with the exception of the report from Foundation Medicine). The aggregate results of any conducted research will be available in accordance with the effective Sponsor policy on study data publication (see Section 9.5).

Data generated by this study must be available for inspection upon request by representatives of national and local health authorities, Sponsor monitors, representatives, and collaborators, and the IRB/EC for each study site, as appropriate.

Study data, which may include data on genomic variants and imaging data, may be submitted to government or other health research databases or shared with researchers, government agencies, companies, or other groups that are not participating in this study. These data may be combined with or linked to other data and used for research purposes, to advance science and public health, or for analysis, development, and commercialization of products to treat and diagnose disease. In addition, redacted Clinical Study Reports and other summary reports will be provided upon request (see Section 9.5).

8.5 FINANCIAL DISCLOSURE

Investigators will provide the Sponsor with sufficient, accurate financial information in accordance with local regulations to allow the Sponsor to submit complete and accurate financial certification or disclosure statements to the appropriate health authorities. Investigators are responsible for providing information on financial interests during the course of the study and for 1 year after completion of the study (see definition of end of study in Section 3.3).

9. STUDY DOCUMENTATION, MONITORING, AND ADMINISTRATION

9.1 STUDY DOCUMENTATION

The investigator must maintain adequate and accurate records to enable the conduct of the study to be fully documented, including, but not limited to, the protocol, protocol amendments, Informed Consent Forms, and documentation of IRB/EC and governmental approval. In addition, at the end of the study, the investigator will receive the patient data, including an audit trail containing a complete record of all changes to data.

9.2 PROTOCOL DEVIATIONS

The investigator should document and explain any protocol deviations. The investigator should promptly report any deviations that might have an impact on patient safety and data integrity to the Sponsor and to the IRB/EC in accordance with established IRB/EC policies and procedures. The Sponsor will review all protocol deviations and assess whether any represent a serious breach of Good Clinical Practice guidelines and require reporting to health authorities. As per the Sponsor's standard operating procedures, prospective requests to deviate from the protocol, including requests to waive protocol eligibility criteria, are not allowed.

9.3 SITE INSPECTIONS

Site visits will be conducted by the Sponsor or an authorized representative for inspection of study data, patients' medical records, and eCRFs. The investigator will permit national and local health authorities; Sponsor monitors, representatives, and collaborators; and the IRBs/ECs to inspect facilities and records relevant to this study.

9.4 ADMINISTRATIVE STRUCTURE

This trial will be sponsored and managed by Genentech, Inc. The Sponsor will provide clinical operations management, data management, and medical monitoring.

Approximately [redacted] sites globally will participate to enroll approximately [redacted] patients. Enrollment will occur through an IxRS.

Data will be recorded via an EDC system from Medidata Solutions (New York, NY) using eCRFs; see Section 7.2. Central laboratories will be used for the analyses of and/or management of PK, PD, genotyping, and tissue samples. An independent ECG facility will be used for central analyses of ECGs; however, patient eligibility and patient treatment decisions will be based on local ECG readings and not on the central analyses.

9.5 DISSEMINATION OF DATA AND PROTECTION OF TRADE SECRETS

Regardless of the outcome of a trial, the Sponsor is dedicated to openly providing information on the trial to healthcare professionals and to the public, at scientific congresses, in clinical trial registries, and in peer-reviewed journals. The Sponsor will comply with all requirements for publication of study results. Study data may be shared with others who are not participating in this study (see Section 8.4 for details), and redacted Clinical Study Reports and other summary reports will be made available upon request. For more information, refer to the Roche Global Policy on Sharing of Clinical *Study Information* at the following website:

www.roche.com/roche_global_policy_on_sharing_of_clinical_study_information.pdf

The results of this study may be published or presented at scientific congresses. For all clinical trials in patients involving an IMP for which a marketing authorization application has been filed or approved in any country, the Sponsor aims to submit a journal manuscript reporting primary clinical trial results within 6 months after the availability of the respective Clinical Study Report. In addition, for all clinical trials in patients involving an IMP for which a marketing authorization application has been filed or approved in any country, the Sponsor aims to publish results from analyses of additional endpoints and exploratory data that are clinically meaningful and statistically sound.

The investigator must agree to submit all manuscripts or abstracts to the Sponsor prior to submission for publication or presentation. This allows the Sponsor to protect proprietary information and to provide comments based on information from other studies that may not yet be available to the investigator.

In accordance with standard editorial and ethical practice, the Sponsor will generally support publication of multicenter trials only in their entirety and not as individual center data. In this case, a coordinating investigator will be designated by mutual agreement.

Authorship will be determined by mutual agreement and in line with International Committee of Medical Journal Editors authorship requirements. Any formal publication of the study in which contribution of Sponsor personnel exceeded that of conventional monitoring will be considered as a joint publication by the investigator and the appropriate Sponsor personnel.

Any inventions and resulting patents, improvements, and/or know-how originating from the use of data from this study will become and remain the exclusive and unburdened property of the Sponsor, except where agreed otherwise.

9.6 PROTOCOL AMENDMENTS

Any protocol amendments will be prepared by the Sponsor. Protocol amendments will be submitted to the IRB/EC and to regulatory authorities in accordance with local regulatory requirements.

Approval must be obtained from the IRB/EC and regulatory authorities (as locally required) before implementation of any changes, except for changes necessary to eliminate an immediate hazard to patients or changes that involve logistical or administrative aspects only (e.g., change in Medical Monitor or contact information).

10. REFERENCES

[redacted]

Amodio V, Yaeger R, Arcella P, et al. EGFR blockade reverts resistance to KRAS ^{G12C} inhibition in colorectal cancer. Cancer Disc 2020;10:1129–39.

[redacted]

Bonner JA, Harari PM, Giralt J, et al. Radiotherapy plus cetuximab for squamous-cell carcinoma of the head and neck. N Eng J Med 2006;354:567–78.

[redacted]

Capon DJ, Seeburg PH, McGrath JP, et al. Activation of Ki-ras2 gene in human colon and lung carcinomas by two different point mutations. Nature 1983;304:507–13.

[redacted]

[redacted]

[FDA] U.S. Food and Drug Administration. List of Cleared or Approved Companion Diagnostic Devices (In Vitro and Imaging Tools). [resource on the Internet]. 2020 [updated: 8 January 2020; cited: 8 March 2020]. Available from: https://www.fda.gov/medical-devices/vitro-diagnostics/list-cleared-or-approved-companion-diagnostic-devices-vitro-and-imaging-tools

[redacted]

Giannakis M, Mu XJ, Shukla SA, et al. Genomic correlates of immune-cell infiltrates in colorectal carcinoma. Cell Reports 2016;15:857–65.

[redacted]

Hartmaier RJ, Charo J, Fabrizio D, et al. https://cancerres.aacrjournals.org/content/77/9/2464 Additional analysis was performed using the Q3 2019 freeze of the Foundation Medicine database.

[redacted]

[redacted]

Karapetis CS, Khambata-Ford S, Jonker DJ, et al. K-ras Mutations and benefit from cetuximab in advanced colorectal cancer. N Engl J Med 2008;359:1757–65.

[redacted]

Kopetz S, Grothey A, Yaeger R, et al. Encorafenib, binimetinib, and cetuximab in BRAF V600E–mutated colorectal cancer. New Eng J Med 2019;381:1632–43.

Krishnan G, D'Silva K, Al-Janadi A.I. Cetuximab-related tumor lysis syndrome in metastatic colon carcinoma. J Clin Oncol 2008;26:2406–8.

[redacted]

McCormick F. Progress in targeting RAS with small molecule drugs. Biochemical J 2019;476:365–74.

Merad M, Martin JC. Pathological inflammation in patients with COVID-19: a key role for monocytes and macrophages. Nat Rev Immunol 2020;20:355–62.

Misale S, Yaegner R, Hobor S, et al. Emergence of KRAS mutations and acquired resistance to anti-EGFR therapy in colorectal cancer. Nature 2012;486:532–36.

[redacted]

NCCN Guidelines Version 1.2020 (b). Colon Cancer.

[redacted]

Ostrem JM, Peters U, Sos ML, et al. K-Ras(G12C) inhibitors allosterically control GTP affinity and effector interactions. Nature 2013;503:548–51.

[redacted]

Parada LF, Tabin CJ, Shih C, et al. Human EJ bladder carcinoma oncogene is homologue of Harvey sarcoma virus ras gene. Nature 1982;297:474–8.

[redacted]

[redacted]

Ryan MB, de la Cruz FF, Phat S, et al. Vertical pathway inhibition overcomes adaptive feedback resistance to KRASG12C inhibition. Clin Cancer Res 2020;26:1633–43.

Santos E, Tronick SR, Aaronson SA, et al. T24 human bladder carcinoma oncogene is an activated form of the normal human homologue of BALB- and Harvey-MSV transforming genes. Nature 1982;298:343–7.

[redacted]

Taparowsky E, Suard Y, Fasano O, et al. Activation of the T24 bladder carcinoma transforming gene is linked to a single amino acid change. Nature 1982;300:762–5.

Van Cutsem EV, Kohne C, Hitre E, et al. Cetuximab and chemotherapy as initial treatment for metastatic colorectal cancer. N Engl J Med 2009;360:1408–17.

[redacted]

[redacted]

Wan JCM, Massie C, Garcia-Corbacho J, et al. Liquid biopsies come of age: towards implementation of circulating tumour DNA. Nat Rev Cancer 2017;17:223–38.

Wan XB, Wang AQ, Cao J, et al. Relationships among KRAS mutation status, expression of RAS pathway signaling molecules, and clinicopathological features and prognosis of patients with colorectal cancer. World J Gastroenterol 2019;25:808–23.

[redacted]

[redacted]

[redacted]

[redacted]

[redacted]

[redacted]

Appendix 2
Schedule of Activities: Stage I and Stage II for Combination Arms [redacted] C, [redacted] [redacted]

	Screening [a]		Cycle 1				Cycles 2–6		Cycles ≥7	Study Treatment Discontinuation [b]
**Cycle Day (Window)**	**–28 to –1**	**–14 to –1**	**1**	**2**	**8 (±1)**	**15 (±1)**	**1 (±1)**	**15 (±1)**	**1 (±3)**	**Within 28 days of final dose**
Informed consent [c]	x									
Demographic data [d]	x									
Medical history and baseline conditions [e]	x									
ECOG performance status	x		x		x		x		x	x
Vital signs [f]	x		x		x	x	x	x	x	x
Weight and height (height at screening only)	x		x		x	x	x	x	x	x
Complete physical examination [g]	x		x				x		x	
Limited physical examination [h]					x	x		x		x
Triplicate 12-lead ECG	Refer to Appendix 3									
Hematology [i]		x	x		x	x	x	x	x	x
Coagulation [j]		x	x		x		x		x	x
Chemistry panel [k]		x	x		x	x	x	x	x	x
Lipid profile [l]		x	x				x		x	x
Amylase, lipase		x	x		x		x		x	x
Pregnancy test [m]		x	*x*				x		x	x
[redacted]										
Tumor assessment [o]	x		Approximately every 6th week from Cycle 1 Day 1							x

	Screening [a]		Cycle 1				Cycles 2–6		Cycles ≥7	Study Treatment Discontinuation [b]
Cycle Day (Window)	–28 to –1	–14 to –1	1	2	8 (±1)	15 (±1)	1 (±1)	15 (±1)	1 (±3)	Within 28 days of final dose
OPTIONAL - FDG-PET [p]	x					x				
Blood sample for *KRAS G12C* mutation status	Refer to Appendix 3									
Plasma sample for biomarkers	Refer to Appendix 3									
Blood sample for biomarker NGS	Refer to Appendix 3									
Blood sample for WGS	Refer to Appendix 3									
[redacted]	[redacted]									
[redacted]	[redacted]									
Tumor tissue for biomarkers	Refer to Appendix 3									
Concomitant medications [q]	x	x	x	x	x	x	x	x	x	x
Adverse events [r]	x	x	x	x	x	x	x	x	x	x

[redacted]

Appendix 2: Schedule of Activities: Stage I and Stage II for Combination Arms [redacted] C, [redacted]

	Screening [a]		Cycle 1				Cycles 2–6		Cycles ≥7	Study Treatment Discontinuation [b]
Cycle Day (Window)	–28 to –1	–14 to –1	1	2	8 (±1)	15 (±1)	1 (±1)	15 (±1)	1 (±3)	Within 28 days of final dose
**ARM C (+ cetuximab)**										
GDC-6036 administration [s]			x	x	x	x	x	x	x	
Cetuximab administration [w]			Weekly starting at Cycle 1 Day 1 [f]							
PK and ADA samples	Refer to Appendix 3									

[redacted]

Appendix 2: Schedule of Activities: Stage I and Stage II for Combination Arms [redacted] C, [redacted]

	Screening [a]		Cycle 1				Cycles 2–6		Cycles ≥7	Study Treatment Discontinuation [b]
Cycle Day (Window)	–28 to –1	–14 to –1	1	2	8 (±1)	15 (±1)	1 (±1)	15 (±1)	1 (±3)	Within 28 days of final dose
[redacted]		[redacted]					[redacted]		[redacted]	[redacted]
PK samples	*Refer to Appendix 3*									

ADA = anti-drug antibody; ECOG = Eastern Cooperative Oncology Group; FFPE = formalin-fixed, paraffin-embedded; HBV = hepatitis B virus; HCV = hepatitis C virus; NGS = next-generation sequencing; PK = pharmacokinetic; RECIST v1.1 = Response Evaluation Criteria in Solid Tumors, Version 1.1; WGS = whole genome sequencing.

Notes: On treatment days, all assessments should be performed prior to dosing, unless otherwise specified. Some assessments may be done outside the window indicated to accommodate holidays and unforeseen scheduling issues, depending on the assessment, and after consultation with the Medical Monitor. The investigator should review local laboratory results prior to dosing.

[a] **SCREENING:** Perform within 28 days prior to Day 1, with the exception of laboratory assessments, which must be obtained within 14 days prior to Day 1. Results of standard-of-care tests or examinations performed prior to obtaining informed consent and within 28 days prior to Day 1 of Cycle 1 may be used for screening assessments rather than repeating such tests.

[b] **STUDY TREATMENT DISCONTINUATION:** Patients who discontinue study drug will return to the clinic for a treatment discontinuation visit within 28 days after their final dose of study drug. In addition, patients will be followed for safety outcomes for a treatment-specific period after their final dose of study drug or until they receive another anti-cancer therapy, whichever occurs first (see Section 5.3.1).

[c] **INFORMED CONSENT:** Informed consent must be documented before any study-specific screening procedure is performed, and may be obtained up to 28 days before initiation of study treatment.

[d] **DEMOGRAPHIC DATA:** Demographic data include age, sex, and self-reported race/ethnicity.

[e] **MEDICAL HISTORY AND BASELINE CONDITIONS:** Refer to Section 4.5.2 for the baseline conditions to be recorded.

[f] **VITAL SIGNS**: Vital signs include respiratory rate, pulse rate, and systolic and diastolic blood pressure while the patient is in a seated position, and temperature. In addition, oxygen saturation will be measured at the screening visit only. Obtain vital signs with PK sample collection (see Appendix 3). Additionally, for patients in the cetuximab arm, vital signs should be measured weekly on the day of cetuximab administration.

[g] **COMPLETE PHYSICAL EXAMINATION:** Complete physical examination includes evaluation of the head, eyes, ears, nose, and throat, and the cardiovascular, dermatologic, musculoskeletal, respiratory, gastrointestinal, genitourinary, and neurologic systems.

[h] **LIMITED PHYSICAL EXAMINATION:** Perform a limited, symptom-directed examination at specified timepoints or as clinically indicated.

[i] **HEMATOLOGY:** Hematology panel includes WBC count, RBC count, hemoglobin, hematocrit, reticulocyte count, platelet count, and differential count (neutrophils, eosinophils, basophils, monocytes, lymphocytes, other cells). Hematology panel may be obtained up to 1 day prior to scheduled clinic visit day.

[j] **COAGULATION:** Coagulation panel includes INR, aPTT, and PT. Coagulation panel may be obtained up to 1 day prior to scheduled clinic visit day.

[k] **CHEMISTRY PANEL:** Chemistry panel (serum or plasma) includes bicarbonate or total carbon dioxide (if considered standard of care for the region), sodium, potassium, chloride, glucose, BUN or urea, creatinine, total protein, albumin, phosphate, calcium, magnesium, total and direct bilirubin, ALP, ALT, and AST. Chemistry panel may be obtained up to 1 day prior to scheduled clinic visit day.

[l] **LIPIDS:** Lipid profile includes total cholesterol, HDL, LDL, and triglycerides. Lipid profile may be obtained up to 1 day prior to scheduled clinic visit day.

[m] **PREGNANCY TEST:** For women of childbearing potential only, a serum pregnancy test must be performed and documented as negative during screening. Serum or urine pregnancy tests for women of childbearing potential will be performed on Day 1 of *each cycle* and at study *treatment* discontinuation visit. If a urine pregnancy test is positive, GDC-6036 should be held until a confirmatory serum pregnancy test is performed. *Following study treatment discontinuation, patients will undergo monthly pregnancy testing for a treatment-specific period after the final dose of study drug (1 month for GDC-6036,* [redacted] *2 months for cetuximab,* [redacted] *or initiation of another anti-cancer therapy, whichever occurs first (see Section 4.5.6).*

[n] **URINALYSIS:** Urinalysis includes dipstick (pH, specific gravity, glucose, protein, ketones, blood, leukocyte esterase, nitrite) and microscopic examination (sediment, RBCs, WBCs, casts, crystals, epithelial cells, bacteria). [redacted]

[o] **TUMOR ASSESSMENTS:** Assess all sites of disease per RECIST v1.1 at screening, every 6th week from Cycle 1 Day 1, and at the study treatment discontinuation visit. The same imaging method used at screening must be used throughout the study. See Section 4.5.5. Patients who continue on study treatment for greater than 12 months may undergo tumor assessments at intervals per institutional standard of care with Medical Monitor approval. For the discontinuation visit, tumor assessment scans performed within 6 weeks prior to the treatment discontinuation visit do not need to be repeated.

[p] **OPTIONAL FDG-PET SCANS:** For patients who have consented to the optional FDG-PET imaging assessment, FDG-PET will be performed at baseline (up to 28 days prior to Cycle 1 Day 1) and between Days 10-16 of Cycle 1, prior to biopsy procedures whenever possible, if applicable. Refer to the imaging manual for more details.

[q] **CONCOMITANT MEDICATIONS:** Record all medications (e.g., prescription drugs, over-the-counter drugs, vaccines, herbal or homeopathic remedies, nutritional supplements) used by patients within 7 days prior to initiation of study drug.

[r] **ADVERSE EVENTS:** After informed consent has been obtained but prior to initiation of study drug, only serious adverse events caused by a protocol-mandated intervention should be reported. After initiation of study drug, all adverse events will be reported for a treatment-specific period after the final dose of study drug or initiation of another anti-cancer therapy, whichever occurs first (see Section 5.3.1). In addition, the Sponsor should be notified if the investigator becomes aware of any serious adverse event that is believed to be related to prior study drug treatment (see Section 5.6).

[s] **GDC-6036 ADMINISTRATION:** On clinic visit days, administer the dose of GDC-6036 in the clinic. On Day 1, dispense a sufficient number of capsules [redacted] to the patient to last until the next visit or, at the investigator's discretion, until the next cycle. Instruct the patient on GDC-6036 dosing procedures and diary completion (see Section 4.3.2). Dispense diary and review at subsequent visits and perform GDC-6036 accountability.

[t] [redacted]

[redacted]

v [redacted]

w **CETUXIMAB ADMINISTRATION (ARM C):** Cetuximab will be administered at an initial dose of 400 mg/m2 as a 120-minute IV infusion on Day 1 followed by 250 mg/m2 as a 60-minute IV infusion weekly, in 21-day cycles. The maximum infusion rate must not exceed *5 mg/min for the initial administration and 10 mg/min for the subsequent administrations.* Cetuximab should be administered following administration of GDC-6036. On PK collection days (see Appendix 3), the start of the cetuximab administration should be 30 minutes after the administration of GDC-6036.

x [redacted]

[redacted]

[redacted]

[redacted]

Appendix 3
Pharmacokinetic, [redacted], Biomarker, and ECG Schedule: Stages I and II (All Arms)

	Screening (Days –28 to –1)	C1												C2, D1 (±1 D)								C3 to C6, D1 (±1 D), and C7 and Later Cycles, D1 (±3 D)	Tx Discon (±1 day)
		D1								D2	D3	D8 (±1 D)	D15 (±1 D)										
		Pre-dose	0	Postdose (hr)										Pre-dose	0	Postdose (hr)							
				0.5	1	2	3	4	8	Pre-dose	Pre-dose	Pre-dose	Pre-dose			0.5	1	2	3	4	8	Pre-dose	
**All Arms**																							
Blood for *KRAS G12C* mutation status [a]	x																						
Plasma for biomarkers		x [b]											x									Odd Cycles (C3, C5, etc.)	x
Blood for NGS		x [b]																					
Blood for WGS [c]		x [b]																					

[redacted]

Appendix 3: Pharmacokinetic, [redacted] Biomarker, and ECG Schedule: Stages I and II (All Arms)

[redacted]

Appendix 3: Pharmacokinetic, [redacted] Biomarker, and ECG Schedule: Stages I and II (All Arms)

[redacted]

Appendix 3: Pharmacokinetic, [redacted], Biomarker, and ECG Schedule: Stages I and II (All Arms)

[redacted]

	Screening (Days –28 to –1)	C1												C2, D1 (±1 D)								C3 to C6, D1 (±1 D), and C7 and Later Cycles, D1 (±3 D)	Tx Discon (±1 day)
		D1								D2	D3	D8 (±1 D)	D15 (±1 D)			Postdose (hr)							
				Postdose (hr)																			
		Pre-dose	0	0.5	1	2	3	4	8	Pre-dose	Pre-dose	Pre-dose	Pre-dose	Pre-dose	0	0.5	1	2	3	4	8	Pre-dose	
[redacted]																							
**Arm C (+ cetuximab)**																							
Tumor tissue for biomarkers	x [e, k]												x [f] (Opt.)										x [g] (Opt.)
Triplicate 12-lead ECG [h]	x	x			x	x	x	x	x	x				x			x	x	x	x	x	*x (single ECG)*	x
Plasma PK sample (*GDC-6036*) [i]		x		x	x	x	x	x	x	x		x	x	x		x	x	x	x	x	x	Odd Cycles	
Serum PK sample (cetuximab)		x		x								x	x	x								C3, C4, C8, C12, and C16	x
Serum ADA sample (cetuximab)		x												x								C3, C4, C8, C12, and C16	x
GDC-6036 administration			x							x	x	x	x		x							QD	
Cetuximab administration			x									x	x		x							QW	

[redacted]

[redacted]

[redacted]

[redacted]

ADA = anti-drug antibody; C = cycle; D = day; NGS = next-generation sequencing; Opt. = optional; PK = pharmacokinetic; WGS = whole genome sequencing.

Notes: Except where noted, all sample draw times are ± 10 minutes. For the PK samples, the plasma will be split into two equal aliquots: a primary sample and a backup sample.

Unless otherwise instructed, GDC-6036 [redacted] should be taken on an empty stomach (i.e., food should be avoided for at least 2 hours before and at least 1 hour post dose). However, water is allowed both before and after dose, as needed. Importantly, GDC-6036 capsules [redacted] will be swallowed whole (not chewed) with at least 240 mL (8 fl oz) of water. On the day of GDC-6036 dosing in the clinic, patients may receive a standard low-fat meal at 1 hour post dose.

[a] **BLOOD SAMPLE for *KRAS G12C* MUTATION STATUS:** Fresh blood sample will be collected at screening from all patients for submission to Foundation Medicine, Inc. Results are required prior to enrollment for patients without prior confirmed *KRAS G12C* status.

[b] **BLOOD SAMPLE FOR NGS, WGS; PLASMA FOR BIOMARKERS; OPTIONAL** [redacted] Can be collected on Day 1 or within 1 week prior to of Day 1.

[c] **BLOOD SAMPLE FOR WGS:** Not applicable for a site that has not been granted approval for WGS.

[d] [redacted]

[e] **TUMOR TISSUE FOR BIOMARKERS (SCREENING):** Archival or fresh pre-treatment FFPE tumor sample. Confirmation of availability is required for Stage I, and confirmation of shipment is required for Stage II. If fresh pre-treatment sample is collected, fresh frozen sample is also requested, if feasible.

[f] **FRESH TUMOR TISSUE FOR BIOMARKERS (ON-TREATMENT):** The on-treatment biopsy will be collected for patients enrolled to [redacted], and for patients in other cohorts who sign the consent for optional on-treatment biopsy. The tumor biopsy (fresh frozen and FFPE tumor samples) should be collected during Cycle 1 between Days 10–16, approximately 24 hours after taking study drug and before the next daily dosing. Refer to the laboratory manual for more details.

[g] **OPTIONAL FRESH TUMOR TISSUE FOR BIOMARKERS AT PROGRESSION:** For patients who sign the Informed Consent Form for the optional biopsy at disease progression, a tumor biopsy will be collected upon disease progression. *Whenever* possible, the biopsy at disease progression should be collected within 24 hours *and up to 2 weeks after* the patient's last dose of GDC-6036.

[h] **ECG:** Submit all ECGs (triplicate *or single* digital 12-lead) to the diagnostic facility for central review. *Triplicate digital 12-lead ECGs will be collected at screening, during Cycle 1 and Cycle 2, and at study treatment discontinuation.* At screening, at least 2–3 triplicate digital 12-lead ECGs should be performed at times corresponding to the anticipated postdose collection times whenever possible to minimize variability due to circadian effects. Alternatively, screening matched timepoint ECG may be obtained by extracting data from 24-hour ambulatory telemetry recording, if available. A ±15-minute window is acceptable for timepoints less than 8 hours and a 30-minute window is acceptable for all timepoints ≥ 8 hours. *On Day 1 of each cycle starting at Cycle 3, a predose single digital 12-lead ECG will be collected.* If QTc prolongation >500 msec and/or 60 msec longer than baseline value is noted *at any time*, repeat ECG until the prolongation is reversed or stabilized, evaluate for causes of QT prolongation, such as electrolyte imbalances and/or concomitant medications known to prolong QT interval, and notify the Medical Monitor. See ECG manual and required ECG Information Form completion.

[i] **PK SAMPLES:** Predose plasma PK samples should be collected within 5 minutes prior to dosing but may be obtained up to 2 hours prior to dosing. When the predose PK sample is on a day following GDC-6036 dosing, the exact time of dosing as well as PK draw (clock-time) should be noted. *All post-dose draw times are ±10 minutes.*

[j] [redacted]

[k] **TUMOR TISSUE FOR BIOMARKERS (SCREENING):** For patients in Arm C (cetuximab), [redacted] *(GDC-1971)* who have been previously treated with a *KRAS G12C* inhibitor, an archival tumor tissue specimen collected prior to treatment with the KRAS G12C inhibitor (e.g., diagnostic specimen) and a recently acquired tumor specimen after completion of the last *KRAS G12C* inhibitor treatment are required.

[l] [redacted]

[m] [redacted]

[redacted]

[redacted]

Appendix 4
Eastern Cooperative Oncology Group Performance Status Scale

Grade	Description
0	Fully active, able to carry on all pre-disease performance without restriction
1	Restricted in physically strenuous activity but ambulatory and able to carry out work of a light or sedentary nature; e.g., light housework or office work
2	Ambulatory and capable of all self-care but unable to carry out any work activities; up and about > 50% of waking hours
3	Capable of only limited self-care, confined to a bed or chair > 50% of waking hours
4	Completely disabled; cannot carry on any self-care; totally confined to bed or chair
5	Dead

Appendix 5
Response Evaluation Criteria in Solid Tumors, Version 1.1 (RECIST v1.1)

Selected sections from the Response Evaluation Criteria in Solid Tumors, Version 1.1 (RECIST v1.1), (Eisenhauer et al. 2009) are presented below, with slight modifications from the original publication and the addition of explanatory text as needed for clarity.[1]

TUMOR MEASURABILITY

At baseline, tumor lesions/lymph nodes will be categorized as measurable or non-measurable as described below. All measurable and non-measurable lesions should be assessed at screening and at subsequent protocol-specified tumor assessment timepoints. Additional assessments may be performed as clinically indicated for suspicion of progression.

DEFINITION OF MEASURABLE LESIONS

Tumor Lesions

Tumor lesions must be accurately measured in at least one dimension (longest diameter in the plane of measurement is to be recorded) with a minimum size as follows:

- 10 mm by computed tomography (CT) or magnetic resonance imaging (MRI) scan (CT/MRI scan slice thickness/interval $\leq$5 mm)
- 10-mm caliper measurement by clinical examination (lesions that cannot be accurately measured with calipers should be recorded as non-measurable)
- 20 mm by chest X-ray

Malignant Lymph Nodes

To be considered pathologically enlarged and measurable, a lymph node must be $\geq$15 mm in the short axis when assessed by CT scan (CT scan slice thickness recommended to be $\leq$5 mm). At baseline and follow-up, only the short axis will be measured and followed. Additional information on lymph node measurement is provided below (see "Identification of Target and Non-Target Lesions" and "Calculation of Sum of Diameters").

[1] For consistency within this document, the section numbers and cross-references to other sections within the article have been deleted and minor formatting changes have been made.

DEFINITION OF NON-MEASURABLE LESIONS

Non-measurable tumor lesions encompass small lesions (longest diameter $<$ 10 mm or pathological lymph nodes with short axis $\geq$ 10 mm but $<$ 15 mm) as well as truly non-measurable lesions. Lesions considered truly non-measurable include leptomeningeal disease, ascites, pleural or pericardial effusion, inflammatory breast disease, lymphangitic involvement of skin or lung, peritoneal spread, and abdominal mass/abdominal organomegaly identified by physical examination that is not measurable by reproducible imaging techniques.

SPECIAL CONSIDERATIONS REGARDING LESION MEASURABILITY

Bone lesions, cystic lesions, and lesions previously treated with local therapy require particular comment, as outlined below.

Bone Lesions:

- Technetium-99m bone scans, sodium fluoride positron emission tomography scans, and plain films are not considered adequate imaging techniques for measuring bone lesions. However, these techniques can be used to confirm the presence or disappearance of bone lesions.
- Lytic bone lesions or mixed lytic-blastic lesions with identifiable soft tissue components that can be evaluated by cross-sectional imaging techniques such as CT or MRI can be considered measurable lesions if the soft tissue component meets the definition of measurability described above.
- Blastic bone lesions are non-measurable.

Cystic Lesions:

- Lesions that meet the criteria for radiographically defined simple cysts should not be considered malignant lesions (neither measurable nor non-measurable) since they are, by definition, simple cysts.
- Cystic lesions thought to represent cystic metastases can be considered measurable lesions if they meet the definition of measurability described above. However, if non-cystic lesions are present in the same patient, these are preferred for selection as target lesions.

Lesions with Prior Local Treatment:

- Tumor lesions situated in a previously irradiated area or in an area subjected to other loco-regional therapy are usually not considered measurable unless there has been demonstrated progression in the lesion.

METHODS FOR ASSESSING LESIONS

All measurements should be recorded in metric notation, using calipers if clinically assessed. All baseline evaluations should be performed as close as possible to the treatment start and never more than 4 weeks before the beginning of the treatment.

The same method of assessment and the same technique should be used to characterize each identified and reported lesion at baseline and during the study. Imaging-based evaluation should always be the preferred option.

CLINICAL LESIONS

Clinical lesions will only be considered measurable when they are superficial and $\geq$10 mm in diameter as assessed using calipers (e.g., skin nodules). For the case of skin lesions, documentation by color photography, including a ruler to estimate the size of the lesion, is suggested.

CHEST X-RAY

Chest CT is preferred over chest X-ray, particularly when progression is an important endpoint, since CT is more sensitive than X-ray, particularly in identifying new lesions. However, lesions on chest X-ray may be considered measurable if they are clearly defined and surrounded by aerated lung.

CT AND MRI SCANS

CT is the best currently available and reproducible method to measure lesions selected for response assessment. In this guideline, the definition of measurability of lesions on CT scan is based on the assumption that CT slice thickness is $\leq$5 mm. When CT scans have slice thickness of $>$5 mm, the minimum size for a measurable lesion should be twice the slice thickness. MRI is also acceptable.

If prior to enrollment it is known that a patient is unable to undergo CT scans with intravenous (IV) contrast because of allergy or renal insufficiency, the decision as to whether a non-contrast CT or MRI (without IV contrast) will be used to evaluate the patient at baseline and during the study should be guided by the tumor type under investigation and the anatomic location of the disease. For patients who develop contraindications to contrast after baseline contrast CT is done, the decision as to whether non-contrast CT or MRI (enhanced or non-enhanced) will be performed should also be based on the tumor type and the anatomic location of the disease, and should be optimized to allow for comparison with prior studies, if possible. Each case should be discussed with the radiologist to determine if substitution of these other approaches is possible and, if not, the patient should be considered not evaluable from that point forward. Care must be taken in measurement of target lesions and interpretation of

non-target disease or new lesions on a different modality, since the same lesion may appear to have a different size using a new modality.

ENDOSCOPY, LAPAROSCOPY, ULTRASOUND, TUMOR MARKERS, CYTOLOGY, HISTOLOGY

Endoscopy, laparoscopy, ultrasound, tumor markers, cytology, and histology cannot be utilized for objective tumor evaluation.

ASSESSMENT OF TUMOR BURDEN

To assess objective response or future progression, it is necessary to estimate the overall tumor burden at baseline and use this as a comparator for subsequent measurements.

IDENTIFICATION OF TARGET AND NON-TARGET LESIONS

When more than one measurable lesion is present at baseline, all lesions up to a maximum of five lesions total (and a maximum of two lesions per organ) representative of all involved organs should be identified as target lesions and will be recorded and measured at baseline. This means that, for instances in which patients have only one or two organ sites involved, a maximum of two lesions (one site) and four lesions (two sites), respectively, will be recorded. Other lesions (albeit measurable) in those organs will be considered non-target lesions.

Target lesions should be selected on the basis of their size (lesions with the longest diameter) and be representative of all involved organs, but in addition should lend themselves to reproducible repeated measurements. It may be the case that, on occasion, the largest lesion does not lend itself to reproducible measurement, in which circumstance the next largest lesion that can be measured reproducibly should be selected.

Lymph nodes merit special mention since they are normal anatomical structures that may be visible by imaging even if not involved by tumor. As noted above, pathological nodes that are defined as measurable and may be identified as target lesions must meet the criterion of a short axis of $\geq$15 mm by CT scan. Only the short axis of these nodes will contribute to the baseline sum. The short axis of the node is the diameter normally used by radiologists to judge if a node is involved by solid tumor. Lymph node size is normally reported as two dimensions in the plane in which the image is obtained (for CT, this is almost always the axial plane; for MRI, the plane of acquisition may be axial, sagittal, or coronal). The smaller of these measures is the short axis. For example, an abdominal node that is reported as being 20 mm $\times$ 30 mm has a short axis of 20 mm and qualifies as a malignant, measurable node. In this example, 20 mm should be recorded as the node measurement. All other pathological nodes (those with short axis $\geq$10 mm

but < 15 mm) should be considered non-target lesions. Nodes that have a short axis of < 10 mm are considered non-pathological and should not be recorded or followed.

All lesions (or sites of disease) not selected as target lesions (measurable or non-measurable), including pathological lymph nodes, should be identified as non-target lesions and should also be recorded at baseline. Measurements are not required. It is possible to record multiple non-target lesions involving the same organ as a single item on the Case Report Form (CRF) (e.g., "multiple enlarged pelvic lymph nodes" or "multiple liver metastases").

CALCULATION OF SUM OF DIAMETERS

A sum of the diameters (longest diameter for non–lymph node lesions, short axis for lymph node lesions) will be calculated for all target lesions at baseline and at each subsequent tumor assessment as a measure of tumor burden.

Measuring Lymph Nodes

Lymph nodes identified as target lesions should always have the actual short axis measurement recorded (measured in the same anatomical plane as the baseline examination), even if the node regresses to < 10 mm during the study. Thus, when lymph nodes are included as target lesions, the sum of diameters may not be zero even if complete response criteria are met, since a normal lymph node is defined as having a short axis of < 10 mm.

Measuring Lesions That Become Too Small to Measure

During the study, all target lesions (lymph node and non–lymph node) recorded at baseline should have their actual measurements recorded at each subsequent evaluation, even when very small (e.g., 2 mm). However, sometimes lesions or lymph nodes that are recorded as target lesions at baseline become so faint on CT scan that the radiologist may not feel comfortable assigning an exact measurement and may report them as being too small to measure. When this occurs, it is important that a value be recorded on the CRF, as follows:

- If it is the opinion of the radiologist that the lesion has likely disappeared, the measurement should be recorded as 0 mm.
- If the lesion is believed to be present and is faintly seen but too small to measure, a default value of 5 mm should be assigned and "too small to measure" should be ticked. (Note: It is less likely that this rule will be used for lymph nodes since they usually have a definable size when normal and are frequently surrounded by fat such as in the retroperitoneum; however, if a lymph node is believed to be present and is faintly seen but too small to measure, a default value of 5 mm should be assigned in this circumstance as well and "too small to measure" should also be ticked).

To reiterate, however, if the radiologist is able to provide an actual measurement, that should be recorded, even if it is <5 mm, and in that case "too small to measure" should not be ticked.

Measuring Lesions That Split or Coalesce on Treatment

When non–lymph node lesions fragment, the longest diameters of the fragmented portions should be added together to calculate the sum of diameters. Similarly, as lesions coalesce, a plane between them may be maintained that would aid in obtaining maximal diameter measurements of each individual lesion. If the lesions have truly coalesced such that they are no longer separable, the vector of the longest diameter in this instance should be the maximum longest diameter for the coalesced lesion.

EVALUATION OF NON-TARGET LESIONS

Measurements are not required for non-target lesions, except that malignant lymph node non-target lesions should be monitored for reduction to <10 mm in short axis. Non-target lesions should be noted at baseline and should be identified as "present" or "absent" and (in rare cases) may be noted as "indicative of progression" at subsequent evaluations. In addition, if a lymph node lesion shrinks to a non-malignant size (short axis <10 mm), this should be captured on the CRF as part of the assessment of non-target lesions.

RESPONSE CRITERIA

CRITERIA FOR TARGET LESIONS

Definitions of the criteria used to determine objective tumor response for target lesions are provided below:

- Complete response (CR): Disappearance of all target lesions

  Any pathological lymph nodes must have reduction in short axis to <10 mm.

- Partial response (PR): At least a 30% decrease in the sum of diameters of all target lesions, taking as reference the baseline sum of diameters, in the absence of CR
- Progressive disease (PD): At least a 20% increase in the sum of diameters of target lesions, taking as reference the smallest sum of diameters at prior timepoints (including baseline)

  In addition to the relative increase of 20%, the sum of diameters must also demonstrate an absolute increase of ≥ 5 mm.

- Stable disease (SD): Neither sufficient shrinkage to qualify for CR or PR nor sufficient increase to qualify for PD

CRITERIA FOR NON-TARGET LESIONS

Definitions of the criteria used to determine the tumor response for the group of non-target lesions are provided below. While some non-target lesions may actually be measurable, they need not be measured and instead should be assessed only qualitatively at the timepoints specified in the schedule of activities.

- CR: Disappearance of all non-target lesions and (if applicable) normalization of tumor marker level

  All lymph nodes must be non-pathological in size (< 10 mm short axis).

- Non-CR/Non-PD: Persistence of one or more non-target lesions and/or (if applicable) maintenance of tumor marker level above the normal limits
- PD: Unequivocal progression of existing non-target lesions

SPECIAL NOTES ON ASSESSMENT OF PROGRESSION OF NON-TARGET LESIONS

Patients with Measurable and Non-Measurable Disease

For patients with both measurable and non-measurable disease to achieve unequivocal progression on the basis of the non-target lesions, there must be an overall level of substantial worsening in non-target lesions in a magnitude that, even in the presence of SD or PR in target lesions, the overall tumor burden has increased sufficiently to merit discontinuation of therapy. A modest increase in the size of one or more non-target lesions is usually not sufficient to qualify for unequivocal progression status. The designation of overall progression solely on the basis of change in non-target lesions in the face of SD or PR in target lesions will therefore be extremely rare.

Patients with Non-Measurable Disease Only

For patients with non-measurable disease only, the same general concepts apply as noted above. However, in this instance there is no measurable disease assessment to factor into the interpretation of an increase in non-measurable disease burden. Because worsening in non-measurable disease cannot be easily quantified (by definition, if all lesions are truly non-measurable), a useful test that can be applied when assessing patients for unequivocal progression is to consider if the increase in overall disease burden based on the change in non-measurable disease is comparable in magnitude to the increase that would be required to declare PD for measurable disease, that is, an increase in tumor burden representing an additional 73% increase in volume (which is equivalent to a 20% increase in diameter in a measurable lesion). Examples include an increase in a pleural effusion from "trace" to "large" or an increase in lymphangitic disease from localized to widespread If unequivocal progression is seen, the patient should be considered to have had overall PD at that point. While it would be ideal to have objective criteria to apply to non-measurable disease, the very nature of that disease makes it impossible to do so; therefore, the increase must be substantial.

NEW LESIONS

The appearance of new malignant lesions denotes disease progression; therefore, some comments on detection of new lesions are important. There are no specific criteria for the identification of new radiographic lesions; however, the finding of a new lesion should be unequivocal, that is, not attributable to differences in scanning technique, change in imaging modality, or findings thought to represent something other than tumor (for example, some "new" bone lesions may be simply healing or flare of preexisting lesions). This is particularly important when the patient's baseline lesions show PR or CR. For example, necrosis of a liver lesion may be reported on a CT scan report as a "new" cystic lesion, which it is not.

A lesion identified during the study in an anatomical location that was not scanned at baseline is considered a new lesion and will indicate disease progression.

If a new lesion is equivocal, for example because of its small size, continued therapy and follow-up evaluation will clarify if it represents truly new disease. If repeat scans confirm there is definitely a new lesion, progression should be declared using the date of the initial scan.

CRITERIA FOR OVERALL RESPONSE AT A SINGLE TIMEPOINT

Table 1 provides a summary of the overall response status calculation at each response assessment timepoint for patients who have measurable disease at baseline.

When patients have non-measurable (therefore non-target) disease only, Table 2 is to be used.

Table 1 Criteria for Overall Response at a Single Timepoint: Patients with Target Lesions (with or without Non-Target Lesions)

Target Lesions	Non-Target Lesions	New Lesions	Overall Response
CR	CR	No	CR
CR	Non-CR/non-PD	No	PR
CR	Not all evaluated	No	PR
PR	Non-PD or not all evaluated	No	PR
SD	Non-PD or not all evaluated	No	SD
Not all evaluated	Non-PD	No	NE
PD	Any	Yes or no	PD
Any	PD	Yes or no	PD
Any	Any	Yes	PD

CR = complete response; NE = not evaluable; PD = progressive disease; PR = partial response; SD = stable disease.

Table 2 Criteria for Overall Response at a Single Timepoint: Patients with Non-Target Lesions Only

Non-Target Lesions	New Lesions	Overall Response
CR	No	CR
Non-CR/non-PD	No	Non-CR/non-PD [a]
Not all evaluated	No	NE
Unequivocal PD	Yes or no	PD
Any	Yes	PD

CR = complete response; NE = not evaluable; PD = progressive disease.

[a] "Non-CR/non-PD" is preferred over "stable disease" for non-target disease since stable disease is increasingly used as an endpoint for assessment of efficacy in some trials; thus, assigning "stable disease" when no lesions can be measured is not advised.

MISSING ASSESSMENTS AND NOT-EVALUABLE DESIGNATION

When no imaging/measurement is done at all at a particular timepoint, the patient is not evaluable at that timepoint. If measurements are made on only a subset of target lesions at a timepoint, usually the case is also considered not evaluable at that timepoint, unless a convincing argument can be made that the contribution of the individual missing lesions would not change the assigned timepoint response. This would be most likely to

happen in the case of PD. For example, if a patient had a baseline sum of 50 mm with three measured lesions and during the study only two lesions were assessed, but those gave a sum of 80 mm, the patient will have achieved PD status, regardless of the contribution of the missing lesion.

SPECIAL NOTES ON RESPONSE ASSESSMENT

Patients with a global deterioration in health status requiring discontinuation of treatment without objective evidence of disease progression at that time should be reported as having "symptomatic deterioration." Every effort should be made to document objective progression even after discontinuation of treatment. Symptomatic deterioration is not a descriptor of an objective response; it is a reason for stopping study therapy. The objective response status of such patients is to be determined by evaluation of target and non-target lesions as shown in Table 1 and Table 2.

For equivocal findings of progression (e.g., very small and uncertain new lesions; cystic changes or necrosis in existing lesions), treatment may continue until the next scheduled assessment. If at the next scheduled assessment, progression is confirmed, the date of progression should be the earlier date when progression was suspected.

REFERENCES

Eisenhauer EA, Therasse P, Bogaerts J, et al. New response evaluation criteria in solid tumors: revised RECIST guideline (version 1.1). Eur J Cancer 2009;45:228–47.

Appendix 6
New York Heart Association Functional Classification

NYHA Class	Patient Symptoms
I	No symptoms and no limitation in ordinary physical activity (e.g., shortness of breath when walking, climbing stairs)
II	Mild symptoms (mild shortness of breath and/or angina) and slight limitation during ordinary activity
III	Marked limitation in activity due to symptoms, even during less-than ordinary activity (e.g., walking short distances [20–100 m]); comfortable only at rest
IV	Severe limitations; experiences symptoms even while at rest; mostly bedbound patients

NYHA = New York Heart Association.

[redacted]

[redacted]

[redacted]		
[redacted]	[redacted]	[redacted]
[redacted]	[redacted]	[redacted]
[redacted]	[redacted]	[redacted]
[redacted]	[redacted]	[redacted]
[redacted]	[redacted]	[redacted]
[redacted]	[redacted]	[redacted]
[redacted]	[redacted]	[redacted]
[redacted]	[redacted]	[redacted]
[redacted]	[redacted]	[redacted]
[redacted]	[redacted]	[redacted]
[redacted]	[redacted]	[redacted]
[redacted]	[redacted]	[redacted]
[redacted]	[redacted]	[redacted]
[redacted]	[redacted]	[redacted]
[redacted]	[redacted]	[redacted]
[redacted]	[redacted]	[redacted]

[redacted]

[redacted]		
[redacted]	[redacted]	[redacted]
[redacted]	[redacted]	[redacted]
[redacted]	[redacted]	[redacted]
[redacted]	[redacted]	[redacted]
[redacted]	[redacted]	[redacted]
[redacted]	[redacted]	[redacted]
[redacted]	[redacted]	[redacted]
[redacted]	[redacted]	

[redacted]

[redacted]

[redacted]

[redacted]	[redacted]
[redacted]	[redacted]
[redacted]	[redacted]
[redacted]	[redacted]

[redacted]

- [redacted]
- [redacted]
- [redacted]

[redacted]

[redacted]

[redacted]

[redacted]

[redacted]

[redacted]	[redacted]
[redacted]	
[redacted]	[redacted]
[redacted]	[redacted]

[redacted]

- [redacted]
- [redacted]
- [redacted]

[redacted]

[redacted] [redacted]

[redacted]	[redacted]
[redacted]	
[redacted]	[redacted]

[redacted]

[redacted]

[redacted]

[redacted]

[redacted]	[redacted]
[redacted]	[redacted]
[redacted]	[redacted]
[redacted]	[redacted]

[redacted]

[redacted]

[redacted]	[redacted]
[redacted]	[redacted]

[redacted]

[redacted]

[redacted]

[redacted]

[redacted]

[redacted]	[redacted]
[redacted]	[redacted]
[redacted]	[redacted]
[redacted]	[redacted]
[redacted]	[redacted]

[redacted]

[redacted]

[redacted]

[redacted]	[redacted]
[redacted]	[redacted]
[redacted]	[redacted]
[redacted]	[redacted]

[redacted]

[redacted]

[redacted]	[redacted]
[redacted]	[redacted]
[redacted]	[redacted]

[redacted]

[redacted]

[redacted]

[redacted]

[redacted]	[redacted]
[redacted]	[redacted]
[redacted]	[redacted]
[redacted]	[redacted]

[redacted]

[redacted]

[redacted]

[redacted]

[redacted]

[redacted]

[redacted]	[redacted]
[redacted]	[redacted]
[redacted]	[redacted]

[redacted]

[redacted]

[redacted]

[redacted]

[redacted]

[redacted]

[redacted]

[redacted]

[redacted]	[redacted]
[redacted]	[redacted]
[redacted]	[redacted]

[redacted] [redacted]

[redacted]	[redacted]
[redacted]	[redacted]
[redacted]	[redacted]

[redacted]

[redacted]

[redacted]

[redacted]

[redacted]

[redacted] [redacted]

[redacted]	[redacted]
[redacted]	[redacted]
[redacted]	[redacted]

[redacted]

[redacted]

[redacted]	[redacted]
[redacted]	[redacted]
[redacted]	[redacted]

[redacted]

[redacted]

[redacted]

[redacted]

[redacted]

[redacted]	[redacted]
[redacted]	[redacted]
[redacted]	[redacted]
[redacted]	[redacted]
[redacted]	[redacted]
[redacted]	[redacted]

a [redacted]

[redacted]

[redacted]

[redacted]

[redacted]

[redacted]	[redacted]
[redacted]	[redacted]
[redacted]	[redacted]
[redacted]	[redacted]
[redacted]	[redacted]

[redacted]

[redacted]

[redacted]

[redacted]

[redacted]

[redacted]

[redacted]

[redacted]	[redacted]
[redacted]	[redacted]

[redacted]

[redacted]

[redacted]

[redacted]

[redacted]

[redacted]

[redacted]

[redacted]	[redacted]
[redacted]	[redacted]
[redacted]	[redacted]
[redacted]	[redacted]

[redacted]

[redacted]

[redacted]

[redacted]

[redacted]

[redacted]

[redacted]	[redacted]
[redacted]	[redacted]
[redacted]	[redacted]

[redacted]

[redacted]

[redacted]

[redacted]	[redacted]
[redacted]	[redacted]

[redacted]

[redacted]

[redacted]

[redacted]

[redacted]

[redacted]

[redacted]

[redacted]

[redacted]

[redacted]

[redacted]

[redacted]	[redacted]
[redacted]	[redacted]

[redacted]

[redacted]

[redacted]

[redacted]

[redacted]

[redacted]

[redacted]

[redacted]

[redacted]

[redacted]

[redacted]

[redacted]

[redacted]

Appendix 9
Guidelines for Management of Patients Who Experience Adverse Events with Cetuximab

The safety profile of cetuximab is presented in the U.S. Package Insert and E.U. SmPC.

Management guidelines for cetuximab based on the occurrence of cetuximab treatment-related AEs that are applicable to this study are provided.

See Appendix 13 for anaphylaxis precautions.

GI toxicities (diarrhea, nausea, vomiting) and elevated hepatic enzymes are potential overlapping toxicities for combination treatments with GDC-6036 and cetuximab. For patients who experience GI toxicities or elevated hepatic enzymes while receiving GDC-6036 in combination with cetuximab, please also see GDC-6036 management guidelines in Section 5.1.7.

CETUXIMAB INFUSION-RELATED REACTIONS AND CYTOKINE RELEASE SYNDROME

Administration of cetuximab will be performed in a monitored setting where there is immediate access to trained personnel and adequate equipment and medicine to manage potentially serious reactions. Special attention is recommended for patients with reduced performance status and preexisting cardiopulmonary disease. For anaphylaxis precautions, see *Appendix 13*. The investigator must ensure that the intended premedication is suitable for participants according to national prescribing information. Prior to the first infusion, participants must receive premedication with an antihistamine *with or without* a corticosteroid *based on local prescribing information*. This premedication is recommended prior to all subsequent infusions (see local prescribing information for cetuximab [Erbitux U.S. Package Insert; Erbitux E.U. SmPC; or equivalent country-specific document]). Close monitoring is required during the infusion and for at least 1 hour after the end of the infusion.

Mild or moderate IRRs are very common, comprising symptoms such as fever, chills, dizziness, or dyspnea that occur in a close temporal relationship mainly to the first cetuximab infusion. If the patient experiences a mild or moderate IRR, the infusion rate may be decreased. It is recommended to maintain this lower infusion rate in all subsequent infusions.

If during the first infusion with cetuximab an IRR occurs within the first 15 minutes, the infusion should be stopped. A careful benefit/risk assessment should be undertaken including consideration of whether the patient may have IgE antibodies before a subsequent infusion is given. If an IRR develops later during the infusion or at a subsequent infusion, further management will depend on its severity.

A cytokine-release syndrome (CRS) typically occurs within 1 hour after infusion and is less commonly associated with bronchospasm and urticaria. CRS is normally most severe in relation to the first infusion.

Management guidelines for cetuximab-related infusion-related reactions and CRS are described in Table 1.

Table 1 Management Guidelines for Cetuximab Related Infusion-Related Reactions and Cytokine-Release Syndrome

Event	Management
Grade 1 [a]<br>Fever [b] with or without constitutional symptoms	• Immediately interrupt cetuximab infusion.<br>• Upon symptom resolution, wait for 30 minutes and then restart infusion at half the rate being given at the time of event onset.<br>• If the infusion is tolerated at the reduced rate for 30 minutes, the infusion rate may be increased to the original rate.<br>• If symptoms recur, discontinue infusion of this dose.<br>• Administer symptomatic treatment, [c] including maintenance of IV fluids for hydration.<br>• In case of rapid decline or prolonged CRS (> 2 days) or in patients with significant symptoms and/or comorbidities, consider managing as per Grade 2.<br>• For subsequent infusions, consider administration of oral premedication with antihistamines, anti-pyretics, and/or analgesics, and monitor closely for IRRs and/or CRS.
Grade 2 [a]<br>Fever [b] with hypotension not requiring vasopressors<br>**and/or**<br>Hypoxia requiring low-flow oxygen [d] by nasal cannula or blow-by	• Immediately interrupt cetuximab infusion.<br>• Upon symptom resolution, wait for 30 minutes and then restart infusion at half the rate being given at the time of event onset.<br>• If symptoms recur, discontinue infusion of this dose.<br>• Administer symptomatic treatment. [c]<br>• For hypotension, administer IV fluid bolus as needed.<br>• Monitor cardiopulmonary and other organ function closely (in the ICU, if appropriate). Administer IV fluids as clinically indicated, and manage constitutional symptoms and organ toxicities as per institutional practice.<br>• Rule out other inflammatory conditions that can mimic CRS (e.g., sepsis). If no improvement within 24 hours, initiate workup and assess for signs and symptoms of HLH or MAS as described in this appendix.<br>• Consider IV corticosteroids (e.g., methylprednisolone 2 mg/kg/day or dexamethasone 10 mg every 6 hours).<br>• Consider anti-cytokine therapy. [e]<br>• Consider hospitalization until complete resolution of symptoms. If no improvement within 24 hours, manage as per Grade 3, that is, hospitalize patient (monitoring in the ICU is recommended), permanently discontinue cetuximab, and contact Medical Monitor.<br>• If symptoms do not resolve to Grade 1 or better for 3 consecutive days, contact Medical Monitor.

Table 1 Management Guidelines for Cetuximab Related Infusion-Related Reactions and Cytokine-Release Syndrome (cont.)

Event	Management
Grade 3 [a]<br>Fever [b] with hypotension requiring a vasopressor (with or without vasopressin)<br>**and/or**<br>Hypoxia requiring high-flow oxygen [d] by nasal cannula, face mask, non-rebreather mask, or Venturi mask	• Permanently discontinue cetuximab and contact Medical Monitor. [f]<br>• Administer symptomatic treatment. [c]<br>• For hypotension, administer IV fluid bolus and vasopressor as needed.<br>• Monitor cardiopulmonary and other organ function closely; monitoring in the ICU is recommended. Administer IV fluids as clinically indicated, and manage constitutional symptoms and organ toxicities as per institutional practice.<br>• Rule out other inflammatory conditions that can mimic CRS (e.g., sepsis). If no improvement within 24 hours, initiate workup and assess for signs and symptoms of HLH or MAS as described in this appendix.<br>• Administer IV corticosteroids (e.g., methylprednisolone 2 mg/kg/day or dexamethasone 10 mg every 6 hours).<br>• Consider anti-cytokine therapy. [e]<br>• Hospitalize patient until complete resolution of symptoms. If no improvement within 24 hours, manage as per Grade 4, that is, admit patient to ICU and initiate hemodynamic monitoring, mechanical ventilation, and/or IV fluids and vasopressors as needed; for patients who are refractory to anti-cytokine therapy, experimental treatments may be considered at the discretion of the investigator and in consultation with the Medical Monitor.
Grade 4 [a]<br>Fever [b] with hypotension requiring multiple vasopressors (excluding vasopressin)<br>**and/or**<br>Hypoxia requiring oxygen by positive pressure (e.g., CPAP, BiPAP, intubation and mechanical ventilation)	• Permanently discontinue cetuximab and contact Medical Monitor. [f]<br>• Administer symptomatic treatment. [c]<br>• Admit patient to ICU and initiate hemodynamic monitoring, mechanical ventilation, and/or IV fluids and vasopressors as needed. Monitor other organ function closely. Manage constitutional symptoms and organ toxicities as per institutional practice.<br>• Rule out other inflammatory conditions that can mimic CRS (e.g., sepsis). If no improvement within 24 hours, initiate workup and assess for signs and symptoms of HLH or MAS as described in this appendix.<br>• Administer IV corticosteroids (e.g., methylprednisolone 2 mg/kg/day or dexamethasone 10 mg every 6 hours).<br>• Consider anti-cytokine therapy. [e] For patients who are refractory to anti-cytokine therapy, experimental treatments [g] may be considered at the discretion of the investigator and in consultation with the Medical Monitor.<br>• Hospitalize patient until complete resolution of symptoms.

Table 1 Management Guidelines for Cetuximab Related Infusion-Related Reactions and Cytokine-Release Syndrome (cont.)

ASTCT = American Society for Transplantation and Cellular Therapy; BiPAP = bi-level positive airway pressure; CAR = chimeric antigen receptor; CPAP = continuous positive airway pressure; CRS = cytokine-release syndrome; CTCAE = Common Terminology Criteria for Adverse Events; eCRF = electronic Case Report Form; HLH = hemophagocytic lymphohistiocytosis; ICU = intensive care unit; IRR = infusion-related reaction; MAS = macrophage activation syndrome; NCCN = National Cancer Comprehensive Network; NCI = National Cancer Institute.

Note: The management guidelines have been adapted from NCCN guidelines for management of CAR T-cell–related toxicities (Version 2.2019).

[a] Grading system for management guidelines is based on ASTCT consensus grading for CRS. NCI CTCAE v5.0 should be used when reporting severity of IRRs, CRS, or organ toxicities associated with CRS on the Adverse Event eCRF. Organ toxicities associated with CRS should not influence overall CRS grading.

[b] Fever is defined as temperature ≥ 38°C not attributable to any other cause. In patients who develop CRS and then receive anti-pyretic, anti-cytokine, or corticosteroid therapy, fever is no longer required when subsequently determining event severity (grade). In this case, the grade is driven by the presence of hypotension and/or hypoxia.

[c] Symptomatic treatment may include oral or IV antihistamines, anti-pyretics, analgesics, bronchodilators, and/or oxygen. For bronchospasm, urticaria, or dyspnea, additional treatment may be administered as per institutional practice.

[d] Low flow is defined as oxygen delivered at ≤ 6 L/min, and high flow is defined as oxygen delivered at > 6 L/min.

[e] There are case reports where anti-cytokine therapy has been used for treatment of CRS with immune checkpoint inhibitors (Rotz et al. 2017; Adashek and Feldman 2019), but data are limited, and the role of such treatment in the setting of antibody-associated CRS has not been established.

[f] Resumption of cetuximab may be considered in patients who are deriving benefit and have fully recovered from the event. Patients can be re-challenged with cetuximab only after demonstrating absence of IgE antibodies against cetuximab and approval has been documented by both the investigator (or an appropriate delegate) and the Medical Monitor. For subsequent infusions, administer oral premedication with antihistamines, anti-pyretics, and/or analgesics, and monitor closely for IRRs and/or CRS. Premedication with corticosteroids and extending the infusion time may also be considered after consulting the Medical Monitor and considering the benefit–risk ratio.

[g] Refer to Riegler et al. (2019) for information on experimental treatments for CRS.

CETUXIMAB ASSOCIATED DERMATOLOGICAL EVENTS

For prevention of EGFR inhibitor-associated dermatologic toxicities, prophylactic antibiotic with doxycycline 100 mg twice daily and topical low potency corticosteroid cream (e.g., hydrocortisone 2.5%) applied twice daily to chest and face for 4-8 weeks starting on Study Day 1, or institutional standard practice, can be used for patients deemed appropriate by the treating physician (Lacouture et al. 2011).

Management guidelines for cetuximab-related dermatological events, including dose delays and dose modifications, are described in *Table 2*. A dose delay of up to 14 days (2 weeks) is allowed for resolution of toxicities.

Note that the dose modification recommendations have been adapted and may deviate from the information presented in local SmPC/package inserts.

Table 2 Management Guidelines for Patients Who Experience Dermatological Events Attributed to Cetuximab

Event	Action to Be Take n
Dermatologic event, Grade 1 or 2	• Initiate maximum supportive care (e.g., antihistamines, topical corticosteroids). Treat per institutional guidelines. Consider treatment with higher-potency topical corticosteroids if event does not improve.<br>• For Grade 2 rash, consider referral to dermatologist.<br><br>Acneiform rash:<br>• Consider topical corticosteroids (e.g., hydrocortisone 2.5%, alclometasone) and oral antibiotics (e.g., minocycline, doxycycline, or antibiotics covering skin flora) twice a day for at least 4 weeks.
Dermatologic event, Grade 3	• Withhold cetuximab.<br>• Refer patient to dermatologist. A biopsy should be performed if appropriate, and, if possible, photographs of the rash should be obtained and submitted to the Sponsor.<br>• If the event does not improve within 48-72 hours, consider treatment with 10 mg/day oral prednisone or equivalent, increasing dose to 1–2 mg/kg/day if event does not improve.<br>• First occurrence: Delay cetuximab administration for up to 14 days (2 weeks) until recovery to NCI CTCAE Grade ≤2 and then restart at 200 mg/m^2. Permanently discontinue if adverse event persists >14 days.<br>• Second occurrence: Delay cetuximab administration for up to 14 days (2 weeks) until recovery to NCI CTCAE Grade ≤ 2 and then restart at 150 mg/m^2. Permanently discontinue if adverse event persists > 14 days.<br>• Third occurrence: Delay cetuximab administration for up to 14 days (2 weeks) until recovery to NCI CTCAE Grade ≤ 2. Permanently discontinue cetuximab if adverse event persists > 14 days.<br><br>Acneiform rash:<br>• Consider continuation of topical corticosteroids (e.g., hydrocortisone 2.5%, alclometasone) and oral antibiotics (e.g., minocycline, doxycycline, or antibiotics covering skin flora) when restarting cetuximab.

Table 2 Management Guidelines for Patients Who Experience Dermatological Events Attributed to Cetuximab (cont.)

Event	Action to Be Taken
Dermatologic event, Grade 4	• Permanently discontinue cetuximab and contact Medical Monitor.

CETUXIMAB-ASSOCIATED PULMONARY EVENTS

Cetuximab can cause interstitial lung disease (ILD). Monitor patients for signs of acute onset of new or progressive unexplained pulmonary events (e.g., dyspnea, cough, fever, or decreased resting oxygen saturation).

All pulmonary events should be thoroughly evaluated for other commonly reported etiologies such as pneumonia or other infection, lymphangitic carcinomatosis, pulmonary embolism, heart failure, chronic obstructive pulmonary disease, or pulmonary hypertension.

Delay cetuximab dosing for up to 14 days pending diagnostic evaluation and resolution to Grade ≤ 1.

Permanently discontinue cetuximab and treat per institutional guideline if ILD is diagnosed. In case of resolution to Grade ≤ 1 within 14 days and ILD not diagnosed, resume dosing at current dose level.

Permanently discontinue cetuximab if Grade 2 adverse events do not improve to Grade 1 within 14 days.

REFERENCES

Adashek ML, Feldman M. Cytokine release syndrome resulting from anti–programmed death-1 antibody: raising awareness among community oncologist. J Oncol Practice 2019;15:502–4.

Lacouture ME, Anadkat MJ, Bensadoun RJ, et al. Clinical practice guidelines for the prevention and treatment of EGFR inhibitor-associated dermatologic toxicities. Support Care 2011;19:1079–95.

Riegler LL, Jones GP, Lee DW. Current approaches in the grading and management of cytokine release syndrome after chimeric antigen receptor T-cell therapy. Ther Clin Risk Manag 2019;15:323–35.

Rotz SJ, Leino D, Szabo S, et al. Severe cytokine release syndrome in a patient receiving PD-1-directed therapy. Pediatr Blood Cancer 2017;64:e26642.

[redacted]

[redacted]

[redacted]

[redacted]

[redacted]

[redacted]

[redacted] [redacted]

[redacted]	[redacted]
[redacted]	[redacted]
[redacted]	[redacted]
[redacted]	[redacted]

[redacted] [redacted]

[redacted]	[redacted]
[redacted]	[redacted]
[redacted]	[redacted]
[redacted]	[redacted]
[redacted]	[redacted]<br>[redacted]
[redacted]	[redacted]
[redacted]	[redacted]

[redacted]

[redacted] [redacted]

[redacted]	[redacted]
[redacted]	[redacted]
[redacted]	[redacted]
[redacted]	[redacted]

[redacted]

[redacted] [redacted]

[redacted]	[redacted]
[redacted]	[redacted]
[redacted]	[redacted]
[redacted]	[redacted]
[redacted]	[redacted]
[redacted]	[redacted]
[redacted]	[redacted]
[redacted]	[redacted]

[redacted]

[redacted]	[redacted]
[redacted]	[redacted]

[redacted]

[redacted]

[redacted]

[redacted]

[redacted]

[redacted]

[redacted] [redacted]

[redacted]	[redacted]
[redacted]	
[redacted]	• [redacted]<br>• [redacted]<br>• [redacted]
[redacted]	• [redacted]

[redacted]

[redacted] [redacted]

[redacted]	[redacted]
[redacted]	
[redacted]	[redacted]
[redacted]	[redacted]
[redacted]	[redacted]

[redacted]

[redacted]	
[redacted]	[redacted]<br>[redacted]
[redacted]	[redacted]<br>[redacted]<br>[redacted]
[redacted]	
[redacted]	[redacted]
[redacted]	[redacted]<br>[redacted]
[redacted]	[redacted]<br>[redacted]<br>[redacted]<br>[redacted]

[redacted] [redacted]

[redacted]	
[redacted]	[redacted] / [redacted] / [redacted] / [redacted]<br>• [redacted]<br>[redacted] / [redacted] / [redacted] / [redacted]<br>• [redacted]<br>• [redacted]<br>– [redacted]<br>• [redacted]<br>– [redacted] / [redacted] / [redacted]<br>• [redacted]

[redacted]	[redacted]

[redacted]

[redacted]

[redacted]	
[redacted]	[redacted]
[redacted]	
[redacted]	[redacted]
[redacted]	[redacted]
[redacted]	[redacted]
[redacted]	[redacted]

[redacted] [redacted]

[redacted]	
[redacted]	[redacted]
[redacted]	[redacted]
[redacted]	[redacted]
[redacted]	[redacted]
[redacted]	
[redacted]	[redacted]
[redacted]	[redacted]

[redacted]

[redacted]

[redacted]

[redacted]

[redacted]

- [redacted]
- [redacted]
- [redacted]
- [redacted]
- [redacted]
- [redacted]

[redacted]

[redacted]

[redacted] [redacted]
[redacted] [redacted]
[redacted] [redacted]
[redacted] [redacted]
[redacted] [redacted]
[redacted] [redacted]
