# Supplementary material for: Divarasib plus cetuximab in KRAS G12C-positive colorectal cancer: a phase 1b trial
Source: Nat Med. 2023 Dec 5;30(1):271–8. doi: 10.1038/s41591-023-02696-8 (PMC10803265; doi:10.1038/s41591-023-02696-8)
Supplement: Supplementary file 2 — Reporting Summary [file 41591_2023_2696_MOESM2_ESM.pdf]

nature portfolio

Corresponding author(s): Jayesh Desai, Sae-Won Han

Last updated by author(s): Nov 8, 2023

Reporting Summary

Nature Portfolio wishes to improve the reproducibility of the work that we publish. This form provides structure for consistency and transparency in reporting. For further information on Nature Portfolio policies, see our Editorial Policies and the Editorial Policy Checklist.

Statistics

For all statistical analyses, confirm that the following items are present in the figure legend, table legend, main text, or Methods section.

n/a	Confirmed	
☐	☒	The exact sample size (*n*) for each experimental group/condition, given as a discrete number and unit of measurement
☐	☒	A statement on whether measurements were taken from distinct samples or whether the same sample was measured repeatedly
☒	☐	The statistical test(s) used AND whether they are one- or two-sided<br>*Only common tests should be described solely by name; describe more complex techniques in the Methods section.*
☒	☐	A description of all covariates tested
☒	☐	A description of any assumptions or corrections, such as tests of normality and adjustment for multiple comparisons
☐	☒	A full description of the statistical parameters including central tendency (e.g. means) or other basic estimates (e.g. regression coefficient) AND variation (e.g. standard deviation) or associated estimates of uncertainty (e.g. confidence intervals)
☒	☐	For null hypothesis testing, the test statistic (e.g. *F*, *t*, *r*) with confidence intervals, effect sizes, degrees of freedom and *P* value noted<br>*Give P values as exact values whenever suitable.*
☒	☐	For Bayesian analysis, information on the choice of priors and Markov chain Monte Carlo settings
☒	☐	For hierarchical and complex designs, identification of the appropriate level for tests and full reporting of outcomes
☒	☐	Estimates of effect sizes (e.g. Cohen's *d*, Pearson's *r*), indicating how they were calculated

Our web collection on statistics for biologists contains articles on many of the points above.

Software and code

Policy information about availability of computer code

Data collection	iMedidata RAVE EDC
Data analysis	SAS version 9.4 to perform all data analyses. Figures 2-4 were produced using R v4.2 (https://www.R-project.org/)

For manuscripts utilizing custom algorithms or software that are central to the research but not yet described in published literature, software must be made available to editors and reviewers. We strongly encourage code deposition in a community repository (e.g. GitHub). See the Nature Portfolio guidelines for submitting code & software for further information.

Data

Policy information about availability of data

All manuscripts must include a data availability statement. This statement should provide the following information, where applicable:

- Accession codes, unique identifiers, or web links for publicly available datasets
- A description of any restrictions on data availability
- For clinical datasets or third party data, please ensure that the statement adheres to our policy

For eligible studies qualified researchers may request access to individual patient level clinical data through a data request platform. At the time of writing this request platform is Vivli: https://vivli.org/ourmember/roche/. Requests for access to Roche data are made through the Vivli process and supported by a research proposal that is assessed by an Independent Review Panel. The panel considers the scientific merit of each application. On average it takes a few months to access data in the Vivli platform, but the timeline will vary depending on the number of data contributors, the number of studies, and your availability to respond to

comments. Analyses performed on the data must be in line with the purpose outlined in the research proposal and be approved by the Independent Review Panel.

For up to date details on Roche's Global Policy on the Sharing of Clinical Information and how to request access to related clinical study documents, see here: https://www.roche.com/innovation/process/clinical-trials/data-sharing. Anonymized records for individual patients across more than one data source external to Roche can not, and should not, be linked due to a potential increase in risk of patient re-identification.

Human research participants

Policy information about studies involving human research participants and Sex and Gender in Research.

Reporting on sex and gender	Sex is reported per protocol
Population characteristics	Reported in Table 1
Recruitment	Patients with colorectal cancer were recruited from clinics at participating institutions from 17 sites in 10 countries. These sites were selected based on factors such as patient population availability and expertise. Patients were recruited, screened, and enrolled at the discretion of the investigator. All patients had local or central blood-based genomic screening that informed eligibility. Attempts to limit bias through study design and site selection were made to create a representative population of patients with CRC, but are limited by this study's small sample size.
Ethics oversight	This study was conducted in full conformance with the ICH E6 guideline for Good Clinical Practice and the Declaration of Helsinki, or the applicable laws and regulations of the country in which the research is conducted, whichever affords the greater protection to the individual. The protocol was approved by the institutional review boards at City of Hope Comprehensive Cancer Center, Princess Margaret Cancer Center, Peter MacCallum Cancer Center, Jewish General Hospital, Universitair Medisch Centrum Utrecht, Biokinetica Przychodnia Jozefow, Istituto Scientifico Romagnolo per lo Studio y la Cura dei Tumori, Asst Grande Ospedale Metropolitano Niguarda, UZ Antwerpen, Hospital Universitario Virgen Del Rocio, Hospital Universitari Vall D'hebron, Seoul National University Hospital, Seoul National University Bundang Hospital, Hospital Clinico Universitario De Valencia, Hospital Universitario 12 De Octubre, Sheba Medical Center, and Abramson Cancer Center. Patients provided signed informed consent prior to enrollment.

Note that full information on the approval of the study protocol must also be provided in the manuscript.

Field-specific reporting

Please select the one below that is the best fit for your research. If you are not sure, read the appropriate sections before making your selection.

☒ Life sciences ☐ Behavioural & social sciences ☐ Ecological, evolutionary & environmental sciences

For a reference copy of the document with all sections, see nature.com/documents/nr-reporting-summary-flat.pdf

Life sciences study design

All studies must disclose on these points even when the disclosure is negative.

Sample size	The study planned to enroll approximately 29 patients in the cetuximab combination cohorts. GO42144 was intended to obtain preliminary safety, PK and activity information and the sample sizes do not reflect any power and type I considerations. However, the expansion sample size is sufficient to provide a meaningful likelihood of observing adverse events occurring at appreciable frequency (5% or higher) and to assess preliminary anti-tumor activity. Details regarding the probability of safety-signal detection with an expansion cohort of 20 patients can be found in Section 6.1.2 of the protocol.
Data exclusions	This analysis included all patients who received at least one dose of divarasib and cetuximab; response rates were reported for patients with measurable disease at baseline
Replication	Replication is not applicable to a clinical trial, as this study was conducted with human participants.
Randomization	This was a non-randomized Phase 1 study where patients were enrolled directly into the divarasib plus cetuximab treatment arm.
Blinding	This was an open-label single-arm study

Reporting for specific materials, systems and methods

We require information from authors about some types of materials, experimental systems and methods used in many studies. Here, indicate whether each material, system or method listed is relevant to your study. If you are not sure if a list item applies to your research, read the appropriate section before selecting a response.

Materials & experimental systems

n/a	Involved in the study
☒	☐ Antibodies
☒	☐ Eukaryotic cell lines
☒	☐ Palaeontology and archaeology
☒	☐ Animals and other organisms
☐	☒ Clinical data
☒	☐ Dual use research of concern

Methods

n/a	Involved in the study
☒	☐ ChIP-seq
☒	☐ Flow cytometry
☒	☐ MRI-based neuroimaging

Clinical data

Policy information about clinical studies

All manuscripts should comply with the ICMJE guidelines for publication of clinical research and a completed CONSORT checklist must be included with all submissions.

Clinical trial registration: ClinicalTrials.gov: NCT04449874

Study protocol: Included in the Supplementary Information.

Data collection: A total of 29 patients were enrolled into Arm C from 17 sites in 10 countries between 28 July 2021 and 07 October 2022. Health care professionals at the clinical trial sites collected samples and recorded data into EDC systems.

Outcomes: The primary objective of this study was to evaluate safety; secondary objectives included characterization of preliminary antitumor activity and the pharmacokinetic profile, and exploratory objectives include the characterization of biomarkers of response and resistance.

Safety was assessed through the evaluation of adverse events (NCI CTCAE v5.0), changes in laboratory test results, and changes in vital signs and ECGs, and included all patients who received at least one dose of both study drugs. Preliminary antitumor activity was determined by the investigator according to RECIST v1.1. Confirmed ORR was defined as the proportion of patients with measurable disease at baseline with CR or PR on two consecutive tumor assessments at least 4 weeks apart, while the best response did not require a confirmatory assessment. PFS was defined as the time from first treatment to the first occurrence of disease progression or death from any cause during the study (whichever occurred first). Pharmacokinetic parameters were estimated using non-compartmental analysis of concentration-time data. Retrospective ctDNA profiling was conducted with longitudinal plasma samples collected at baseline, on-treatment and end-of-treatment visits. To explore the mechanisms of potential acquired resistance, we conducted ctDNA profiling from paired baseline and disease progression/end-of-treatment plasma samples.
